# Increased weight-load improves body composition by reducing fat mass and waist circumference, and by increasing lean mass in participants with obesity: a single-centre randomised controlled trial

**DOI:** 10.1186/s12916-025-04143-6

**Published:** 2025-05-30

**Authors:** Jakob Bellman, Klaas Westerterp, Loek Wouters, Marit Johannesson, Niklas Lundqvist, Joel Kullberg, Christel Larsson, Mikael Gustafsson, Stefan Pettersson, Jonatan Fridolfsson, Daniel Arvidsson, Mats Börjesson, Dan Curiac, John-Olov Jansson, Per-Anders Jansson, Claes Ohlsson

**Affiliations:** 1 Department of Physiology, Institute of Neuroscience and Physiology, Sahlgrenska Academy, University of Gothenburg, Medicinaregatan 11, SE-41390 Gothenburg, Sweden; 2 Department of Nutrition and Movement Sciences, NUTRIM School of Nutrition and Translational Research in Metabolism, Maastricht University Medical Centre, NL-6200 MD Maastricht, The Netherlands; 3 Department of Radiology, Region Västra Götaland, Sahlgrenska University Hospital, SE-41345 Gothenburg, Sweden; 4 Radiology, Department of Surgical Sciences, Uppsala University, SE-75185 Uppsala, Sweden; 5 Antaros Medical, SE-43153 Mölndal, Sweden; 6 Department of Food and Nutrition, and Sport Science, Faculty of Education, University of Gothenburg, SE-40530 Gothenburg, Sweden; 7 Department of Medicine, Geriatric and Acute Medicine, Region Västra Götaland, Sahlgrenska University Hospital, SE-41685 Gothenburg, Sweden; 8 Gothia Forum, Region Västra Götaland, Sahlgrenska University Hospital, SE-41346 Gothenburg, Sweden Gothia Forum,; 9 Wallenberg Laboratory, Department of Molecular and Clinical Medicine, Institute of Medicine, Sahlgrenska Academy, University of Gothenburg, SE-41345 Gothenburg, Sweden; 10 Sahlgrenska Osteoporosis Centre, Centre for Bone and Arthritis Research, Institute of Medicine, Sahlgrenska Academy, University of Gothenburg, SE-41345 Gothenburg, Sweden; 11 Department of Drug Treatment, Region Västra Götaland, Sahlgrenska University Hospital, SE-41345 Gothenburg, Sweden

**Keywords:** Obesity, Weight-bearing, Weight-loading, Standing position, Body composition, Fat mass distribution, Energy balance

## Abstract

**Background:**

To investigate the effects of increased weight-loading on body weight, body composition, fat mass distribution, physical activity and energy balance in individuals with obesity.

**Methods:**

This single-centre non-blinded randomised controlled trial was conducted from August 1, 2021, through February 28, 2022. Adults with obesity class 1 (body mass index, BMI 30–35 kg/m^2^) were assigned to wear either a heavy (high load; 11% of body weight, *n* = 28) or light (low load; 1% of body weight, *n* = 30) weight vest for 8 h per day over 5 weeks.

**Results:**

High-load treatment reduced fat mass (mean difference − 2.60%; 95% CI − 3.79, − 1.41) and increased lean mass (mean difference 1.40%; 95% CI 0.37, 2.42), with no significant effect on body weight. Fat mass reductions were primarily observed in weight-loaded regions but not in the non-weight-bearing regions such as the arms. Waist circumference decreased (mean difference − 2.26%; 95% CI − 3.81, − 0.71) in the high-load group compared to the low-load group. Despite these beneficial changes, sedentary time was higher in the high-load group (mean difference 4.69%; 95% CI 0.98, 8.39) compared to the low-load group, while energy expenditure and energy intake remained unchanged.

**Conclusions:**

Increased weight-loading reduced fat mass and increased lean mass, resulting in a healthier body composition. These effects were achieved despite no increase in physical activity. The fat mass-reducing effect was primarily seen in weight-loaded regions, implying local adaptation to the increased loading.

**Trial registration:**

Registered at ClinicalTrials.gov (NCT04697238) in 2021.

**Supplementary Information:**

The online version contains supplementary material available at 10.1186/s12916-025-04143-6.

## Background

Obesity is a serious public health issue that increases the risk of various chronic diseases and lowers quality of life [1–4]. In recent years, there have been significant advances of incretin-based pharmacotherapies showing promising results as anti-obesity treatments. However, while they effectively reduce fat mass, they are often associated with an undesirable loss of muscle mass [5, 6]. Such a loss of muscle mass could be especially problematic in patients with sarcopenic obesity [7–9]. The regulation of fat mass still remains elusive [10]. Fat mass is determined by the long-term balance between energy intake and expenditure, which is regulated by genetic, environmental, behavioural and hormonal factors [11]. The distribution of fat, rather than fat mass alone, plays a critical role in metabolic health. Ectopic fat accumulation, such as intermuscular adipose tissue (IMAT), visceral adipose tissue (VAT), and liver fat, is closely linked to metabolic disorders, while subcutaneous fat is considered less harmful [12–17]. However, it remains unclear how lifestyle interventions, such as increased standing or weight-loading, influence fat mass and its distribution in humans.

An emerging field of research explores how health outcomes are influenced by exercise and posture allocation. It has been demonstrated that increased time spent standing is associated with improved health including reduced waist circumference, improved insulin sensitivity as well as reduced risk of developing obesity [18–22]. Standing modestly increases energy expenditure and may also raise cardiac workload due to the upright posture, potentially triggering systemic cardiovascular adaptations [23, 24]. However, the metabolic benefits of standing may partly derive from the light physical activity often accompanying it, making it difficult to separate the effects of standing itself from those of increased movement. While some evidence suggests that standing contributes to metabolic health, it is generally agreed that increased physical activity plays a more critical role [25, 26]. Any causal relationships between standing time and fat mass or its distribution are yet to be elucidated, and the evidence regarding the effect of time spent standing on metabolic health is inconclusive [27].

Standing increases weight-bearing on the back and lower limbs, which could have physiological effects beyond posture. Furthermore, increased weight-loading has been reported to reduce fat mass without affecting lean mass in rodents [28, 29]. Mechanistic studies suggest that osteocytes in weight-bearing bones can sense weight-loading and influence fat mass regulation [28, 29]. In addition, data from a number of mouse models, targeting certain genes in osteoblast-lineage cells, have suggested that the skeleton may exert effects on fat mass and energy metabolism [30, 31].

In a previous short-term clinical study, we observed that increased weight-loading, by wearing weight vests for 3 weeks, reduced fat mass without any significant effect on non-fat mass [32]. However, the specific types of adipose tissue influenced by increased weight-loading, and the underlying mechanisms driving these changes remain unclear. The aims of the present 5-week study were to determine the effects of increased weight-loading on body weight, body composition, fat mass distribution, physical activity and energy balance in individuals with obesity. We hypothesised that increased weight-loading would lead to reductions in body weight, fat mass and waist circumference, along with an increase in lean mass.

## Methods

### Study design

**Fig. 1:**
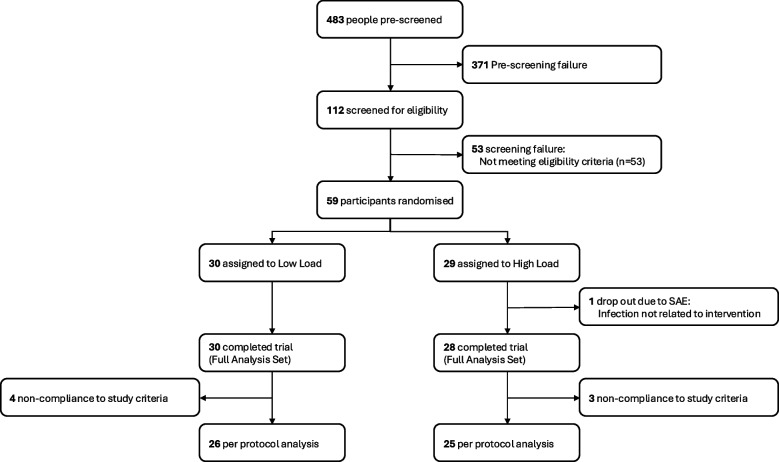
CONSORT Diagram describing enrolment and study flow. The participants with screening failure did not meet all the inclusion criteria and/or did meet at least one of the exclusion criteria. SAE, severe adverse event

This was a single-centre clinical randomised controlled trial (RCT) investigating the effect of increased weight-loading, through application of weight vests, on body weight in individuals with obesity. The trial was conducted at Gothia Forum, Sahlgrenska University Hospital in Gothenburg, Sweden. This trial adheres to the CONSORT (Consolidated Standards of Reporting Trials) guidelines. A CONSORT flow diagram is presented in Fig. 1, and a CONSORT checklist is provided in Additional file 1.

The trial was approved by the Swedish Ethical Review Authority (registration number 2021–00095) and was conducted in accordance with the Declaration of Helsinki and the International Conference on Harmonization Guidelines (ICH) for Good Clinical Practice (GCP). The trial is registered at ClinicalTrials.gov (Anti-obesity Treatment by Loading in Adult Subjects (ATLAS), NCT04697238). An independent monitor oversaw the trial according to ICH-GCP guidelines. The monitor had access to all the data and performed monitoring visits on site before, during and after data collection.

### Participants

Eligible participants were adults (aged 18–65 years) with obesity defined as body mass index (BMI) of 30–35 kg/m^2^. Exclusion criteria included chronic disease that could interfere with study participation such as diabetes (type 1 or type 2) or cardiovascular disease, previous bariatric-metabolic surgery, reduced mobility and chronic pain. Full lists of the inclusion and exclusion criteria are provided in the study protocol (Additional file 2). Data regarding gender (male, female or other) were self-reported at screening. Written informed consent was obtained for all participants before enrolment in the trial.

### Randomisation and masking

A total of 483 people were pre-screened in a phone interview, of which 112 were screened for eligibility. Fifty-nine participants were eligible according to the eligibility criteria and randomised in a 1:1 ratio to 5 weeks treatment with either a heavy weight vest (= high-load group, 11% of participant’s body weight) or a light weight vest (= low-load group, 1% of participant’s body weight). Randomisation was performed through permuted blocks (stratified by age 18–50 years, age 51–65 years and gender) using a built-in function in the electronic case report form (eCRF) software (MediCase eCRF version 5, MediCase AB, Gothenburg, Sweden). The study was not blinded to the participants or study personnel because of the difficulty to conceal differences in weight between treatments (heavy and light weight vest).

### Procedures

**Fig. 2:**
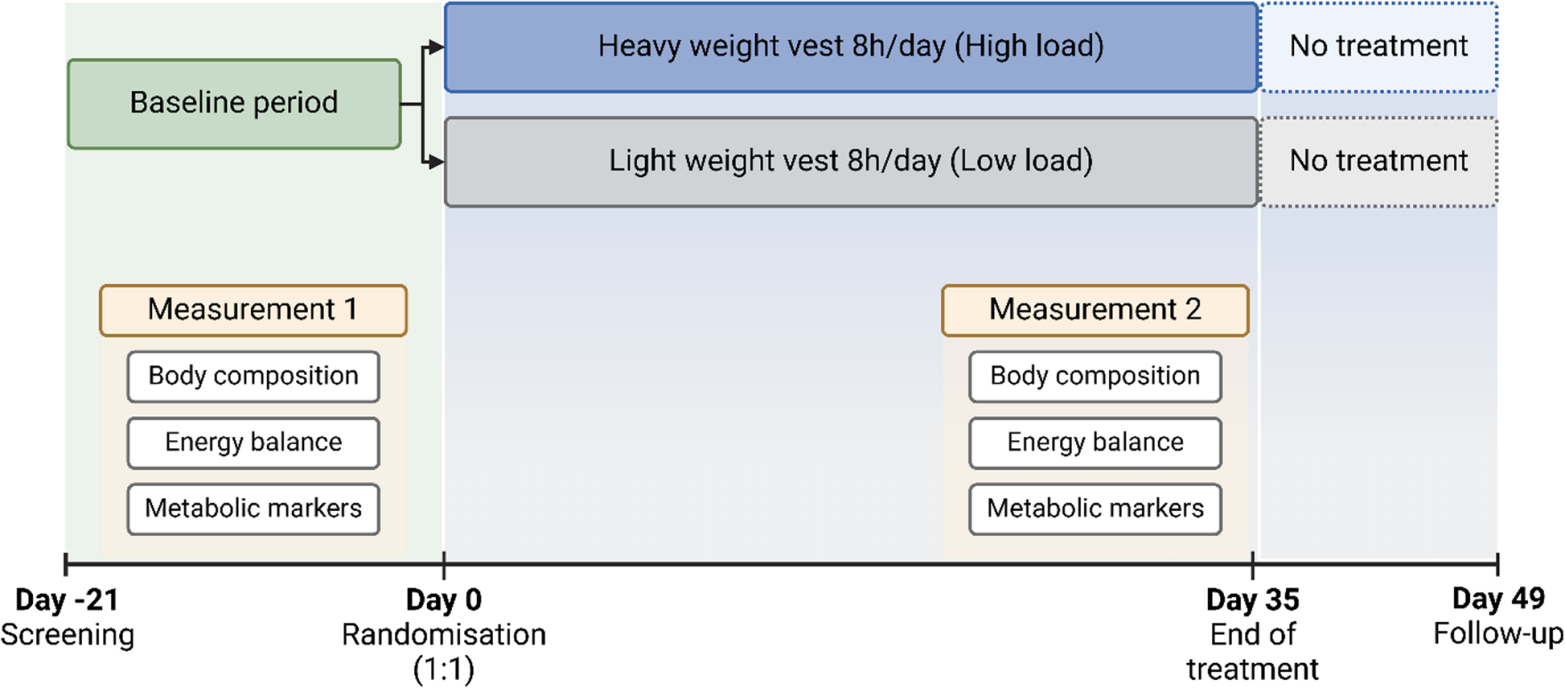
Schematic timeline showing key moments of the trial. Screening performed on day -21. Baseline measurements obtained between day -21 and day 0 (Measurement 1). Measurement 1 included 14-day DLW measurement (day -14), 7-day accelerometer measurement (day -13), DXA and CT scans along with SDQ on day 0. Randomisation on day 0 and treatment with either low load or high load between day 0 and day 35. Second measurement period (Measurement 2) performed between day 14 and day 35. Measurement 2 included 14-day DLW measurement (day 14), 7-day accelerometer measurement (day 15), SDQ (day 28) along with DXA and CT scans on day 35. Follow-up visit on day 49, 2 weeks after the end of treatment. Figure created in BioRender; Bellman, J. (2025) https://BioRender.com/y79d008. CT, computed tomography; DLW, doubly labelled water; DXA, dual-energy X-ray absorptiometry; SDQ, Short Dietary Questionnaire

The trial design is illustrated in Fig. 2. Each participant participated in the trial for approximately 10 weeks, and it involved 13 visits. Overall, the trial consisted of three phases: (1) Weeks 1–3—Baseline measurements, (2) Weeks 4–8—Randomisation and intervention, (3) Weeks 9–10—Follow-up post-treatment. Details about each study visit and its procedures are shown in the study protocol (Additional file 2). Adverse events were recorded at all study visits and when spontaneously reported by the participant, from the time of signing the informed consent form until completion of the study. The main endpoints were evaluated before (day 0) and after intervention (day 35), but some mechanistic endpoints (e.g. physical activity, energy expenditure and energy intake) were measured during the intervention (Fig. 2; for further detail see Additional file 2).

#### Intervention

Eligible participants were randomised at study visit 6 (day 0) into 5-week treatment with low or heavy weight-loading. The heavy loading (high load) consisted of a weight vest with a weight corresponding to 11% of the subject’s body weight (PRF Weight vest, Casall, Norrköping, Sweden) and the low loading (low load) consisted of a weight vest (PRF Weight vest, Casall, Norrköping, Sweden) of identical appearance, but with a weight corresponding to 1% of the subject’s body weight. Each vest was individually loaded with small weight bags (400–600 g each; Casall, Norrköping, Sweden) to reach approximately 1% or 11% of the participant’s body weight. The final weight of each vest was verified using a calibrated scale (seca 704; seca GmbH, Hamburg, Germany) before distribution, with a maximum error margin of ± 0.2 kg.

The 11% and 1% of body weight were chosen to create a 10% difference between the treatment groups, based on a protocol used in a previous 3-week clinical study [32]. Participants were instructed to carry the weight vests for 8 h per day for 5 weeks and to be in the standing position as much as possible during the carrying hours, but at a minimum 2 h per day. Outside of these instructions related to vest use and time spent standing, participants were instructed to maintain their usual lifestyle and daily routines. The participant recorded daily the time using the weight vest and the time using the vest standing. Compliance to wearing the weight vest was evaluated using the participant’s written recordings.

#### Measurements

##### Body composition parameters (Fig. 2)

Body weight (kilograms [kg]) was measured using a calibrated body weight scale (seca scales 704, seca, Hamburg, Germany). Dual-energy X-ray absorptiometry (DXA; Lunar iDXA, enCORE version 16, SP 1, GE Healthcare, Illinois, USA) was used to determine fat mass (kg), lean mass (kg) and bone mineral content (BMC; kg). Computed tomography (CT) scans (Somatom Force, Siemens Healthcare GmbH, Munich, Germany) of the liver and abdomen were conducted to evaluate liver fat (Hounsfield Units [HU]), visceral adipose tissue (VAT; cm^3^) and abdominal subcutaneous adipose tissue (SAT; cm^3^) according to a specific study protocol (Additional file 3: Supplementary methods) [33, 34]. DXA and CT scans were performed on day 0 and day 35. Waist circumference (cm) was measured with a measuring tape.

##### Energy balance (Fig. 2)

Energy expenditure (EE; joules per day) was measured using the Doubly Labelled Water (DLW) method over two separate 14-day measurement periods on days -14 and 14. The measurements was conducted following the standard operating procedure from Maastricht University (Maastricht, Netherlands; Additional file 3: Supplementary methods) [35, 36]. Daily energy intake (joules per day) was estimated using the validated food questionnaire “*Short Dietary Questionnaire*” (SDQ) [37], which participants completed on days 0 and 28. The SDQ estimated the energy intake for the previous 2 weeks at each time point.

##### Physical activity (Fig. 2)

Physical activity (PA) was measured on two 7-day periods starting on days -13 and 15. Participants were instructed to wear the tri-axial accelerometers (Axivity AX3, Axivity Ltd., Newcastle upon Tyne, UK) over the hip for seven consecutive days during the whole day (24 h). Physical activity was categorised into different intensity levels based on metabolic equivalent of tasks (METs) thresholds: sedentary (< 1.5 METs), light PA (LPA; 1.5–2.9), moderate PA (MPA; 3.0–5.9), vigorous PA (VPA; 6.0–8.9) and very vigorous PA (VVPA; ≥ 9.0). Moderate-to-vigorous PA (MVPA) was calculated as the sum of MPA, VPA and VVPA. The method is described in more detail in Additional file 3: Supplementary methods [38–40].

##### Metabolic blood parameters (Fig. 2)

Blood samples were taken to analyse metabolic markers using defined routines and certified assays or at the certified Clinical Chemistry lab at the Sahlgrenska University Hospital (Gothenburg, Sweden) using standardised procedures with the Alinity analysis platform (Abbott, Illinois, USA). The supplementary methods provide more information about all the measurements and methods (Additional file 3).

### Outcomes

The primary endpoint was the percentage change in body weight from randomisation (day 0) to the end of the intervention (day 35) 5 weeks later (Fig. 2). Key secondary endpoints included percentage change from baseline to the end of intervention in waist circumference, body fat percentage, body fat mass, body lean mass, regional fat mass, abdominal adipose tissue (VAT and SAT), liver fat, energy expenditure, energy intake and physical activity levels. All the secondary endpoints were also analysed as absolute changes. All endpoints in this study were compared between the high-load group and the low-load group. Details on all measurements can be found in the provided study protocol (Additional file 2).

### Statistical analysis

Power calculations determined that 25 evaluable participants per group were required to detect a significant effect size of 1.6% difference in relative body weight change between groups from baseline to 5 weeks (primary endpoint), with a power of 80%. The effect size for the sample size calculation was based on results from a previous clinical study that estimated the effect of a 3-week treatment with weight vest on body weight (Additional file 3: Supplementary methods) [32]. The difference between the treatment groups for all parameters were tested by analysis of covariance (ANCOVA) with relative change from baseline to 5 weeks as the dependent variable, treatment group as fixed effect and age, sex, baseline BMI, vest exposure (h/day) and standing% when using the weight vest (= vest time standing/total vest time × 100) as covariates. From these ANCOVA models adjusted for covariates, estimated marginal means with 95% confidence intervals (CI) are presented. The *p*-values given for the within-group comparison (5 weeks vs baseline) of the different parameters were calculated using Wilcoxon signed-rank test. The statistical analyses were performed according to a statistical analysis plan developed before study start, and data analyses were performed using SPSS Statistics 29 (IBM Corp., Armonk, NY, USA) or GraphPad Prism 10 (GraphPad Software, Boston, MA, USA). A *P*-value < 0.05 was considered statistically significant.

### Role of the funding source

The funders of the study had no role in study design, data collection, data analysis, data interpretation, or writing of the report.

## Results

### Study participants

A total of 59 participants were randomised to treatment with low-load (*n* = 30) or high-load (*n* = 29) vests, of which all 30 participants in the low-load group and 28/29 participants in the high-load group completed the trial (= full analysis set [FAS]; Fig. 1). The drop out participant in the high-load group was due to a severe adverse event (infection not related to treatment; Additional file 3: Table S6). Seven participants were excluded from the per-protocol (PP) analysis due to not following the study protocol with regard to vest exposure or not making any lifestyle changes, resulting in 26 participants in the low-load group and 25 participants in the high-load group (Fig. 1). In general, the findings from the full analysis set (presented in Additional file 3: Tables S1 and S2) and the per-protocol analysis (presented in main tables) were very similar. Characteristics of participants at baseline were similar in the two treatment groups (Table 1).

**Table 1:** Baseline characteristics and treatment exposure

**Characteristics**	**Low load** (*n* = 30)	**High load** (*n* = 28)	***P*-value**
Age (years)	42.7 ± 10.9	42.6 ± 10.0	NS
Sex, females (%)	15 (50%)	15 (54%)	NS
Height (cm)	176.0 ± 10.0	172.7 ± 8.4	NS
Weight (kg)	100.8 ± 11.5	98.4 ± 10.1	NS
BMI (kg/m^2^)	32.5 ± 1.7	32.9 ± 1.2	NS
Waist circumference (cm)	110.0 ± 7.6	110.0 ± 6.9	NS
DXA scan			
Fat percent (%)	39.2 ± 5.7	40.3 ± 5.8	NS
Fat mass (kg)	39.1 ± 5.3	39.4 ± 5.7	NS
Lean mass (kg)	58.5 ± 10.6	55.9 ± 9.2	NS
BMC (kg)	3.0 ± 0.5	3.0 ± 0.4	NS
CT scan			
Abdominal visceral fat (cm^3^)	68.8 ± 37.7	63.6 ± 24.8	NS
Abdominal Subcutaneous fat (cm^3^)	187.9 ± 43.7	200.3 ± 39.9	NS
Liver fat (HU)	61.4 ± 10.1	58.2 ± 11.0	NS
Blood pressure			
Systolic (mmHg)	123.8 ± 10.3	122.7 ± 10.3	NS
Diastolic (mmHg)	73.9 ± 8.6	71.5 ± 9.6	NS
Plasma/serum markers			
Total cholesterol (mmol/L)	5.1 ± 1.2	4.9 ± 1.2	NS
LDL cholesterol (mmol/L)	3.5 ± 1.1	3.4 ± 1.1	NS
HDL cholesterol (mmol/L)	1.3 ± 0.4	1.2 ± 0.4	NS
Triglycerides (mmol/L)	1.3 ± 0.5	1.4 ± 0.7	NS
Glycerol (μmol/L)	64.4 ± 27.1	65.3 ± 49.5	NS
Fasting Glucose (mmol/L)	5.6 ± 0.5	5.7 ± 0.9	NS
Insulin (mIU/L)	11.1 ± 8.2	10.5 ± 4.7	NS
HOMA-IR	2.8 ± 2.2	2.7 ± 1.4	NS
Leptin (ng/ml)	33.2 ± 21.7	32.2 ± 16.9	NS
Physical activity			
Mean daily activity (mg)	24.7 ± 8.3	23.9 ± 6.4	NS
Sedentary time (minutes/day)	728.7 ± 59.1	725.4 ± 61.1	NS
LPA (minutes/day)	101.1 ± 29.1	106.0 ± 25.3	NS
MVPA (minutes/day)	68.8 ± 25.3	67.3 ± 23.1	NS
Energy balance			
Energy expenditure (MJ/day)	12.8 ± 1.9	12.2 ± 2.0	NS
Energy intake (MJ/day)	9.1 ± 4.0	7.4 ± 1.9	NS
Treatment exposure			
Vest exposure (hrs/day)	9.7 ± 1.0	9.7 ± 1.1	NS
Standing time (hrs/day)	4.7 ± 1.9	4.5 ± 1.4	NS
Standing (%)	48.7 ± 1.8	46.7 ± 1.6	NS

Values are given as mean ± SD or *n* (%) for all randomised subjects. For comparisons between groups, Fisher’s exact test was used for dichotomous variables, *t*-test was used for normally distributed continuous parameters, and Mann–Whitney *U* test was used for non-normally distributed parameters

*BMC* bone mineral content, *BMI* body mass index, *CT* computed tomography, *DXA* dual-energy X-ray absorptiometry, *HDL* high-density lipoprotein, *HOMA-IR* homeostatic model assessment for insulin resistance, *HU* Hounsfield unit, *LDL* low-density lipoprotein, *LPA* light physical activity, *Mg* milli-g (acceleration), *MJ* megajoules, *MVPA* moderate-vigorous physical activity, *NS* non-significant, *Standing (%)*, proportion of hours standing out of the hours with vest exposure

### Effects of high-load treatment on body composition

**Fig. 3:**
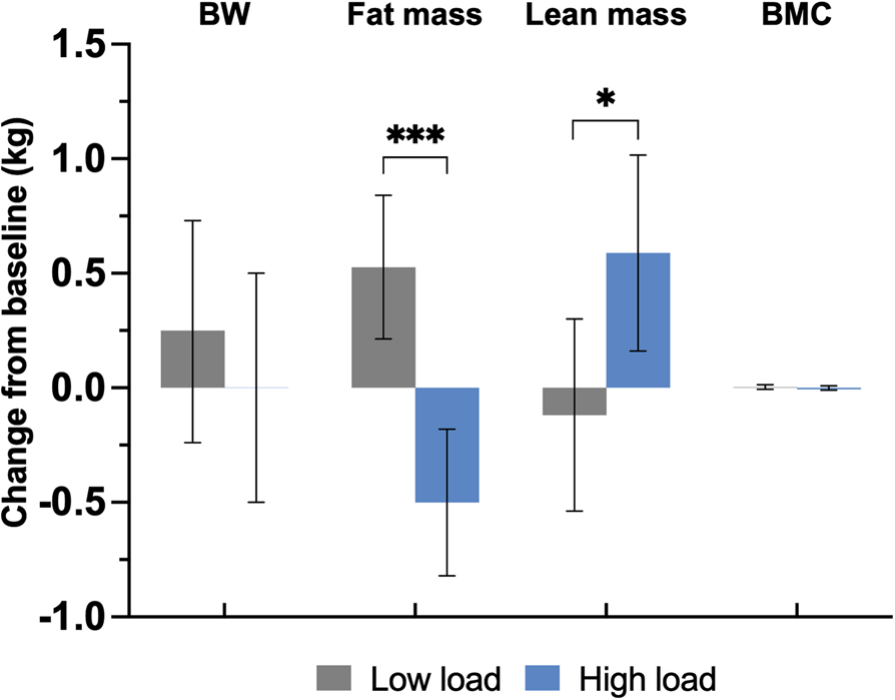
Change in body weight (BW), fat mass, lean mass and bone mineral content (BMC) at 5 weeks vs. baseline for all participants who completed the trial according to protocol. Participants treated with light weight vest (low load; *n* = 26) or heavy weight vest (high load, *n* = 25). Body composition and BMC measured with dual-energy X-ray absorptiometry (DXA). Results are presented as estimated marginal means with error bars showing 95% confidence intervals. The between-group *P*-values (high load vs low load) are calculated using analysis of covariance (ANCOVA) adjusted for age, sex, baseline body mass index (BMI), vest exposure (h) and standing % with vest. *P < 0.05, ***P < 0.001

High-load treatment for 5 weeks reduced fat mass (mean difference − 2.60%; 95% CI − 3.79, − 1.41) and increased lean mass (mean difference 1.40%; 95% CI 0.37, 2.42) compared to the low-load group, with no significant effect on body weight (mean difference − 0.21%; 95% CI − 0.92, 0.50; Fig. 3, Additional file 3: Table S3). Analysis of the absolute changes in body composition demonstrated that fat mass was 1.03 kg lower (95% CI − 1.48, − 0.58) and lean mass was 0.71 kg higher (95% CI 0.10, 1.31) in the high-load group compared to the low-load group (Table 2). No effect was observed on total body bone mineral content (Table 2).

**Table 2:** Analyses of the absolute changes in the main secondary endpoints for all participants eligible for per-protocol analysis

	**Low load** **(*n* = 26)**	**High load** **(*n* = 25)**	**Difference between groups**	***P*-value (ANCOVA)**
Anthropometrics				
Body weight (kg)	0.25 (− 0.24, 0.73)	− 0.0005 (− 0.50, 0.50)	− 0.25 (− 0.95, 0.46)	0.482
Waist circumference (cm)	0.4 (− 0.8, 1.6)	− 2.0 (− 3.2, − 0.8) **	− 2.4 (− 4.2, − 0.7)	**0.006**
DXA scan				
Fat percent (%)	0.35 (0.12, 0.59) *	− 0.66 (− 0.81, − 0.33) ***	− 0.9 (− 1.3, − 0.6)	**2.3E-6**
Fat mass (g)	526.8 (213.4, 840.2) **	− 500.8 (− 820.5, − 181.1) **	− 1027.6 (− 1479.9, − 575.3)	**3.8E-5**
Lean mass (g)	− 119.4 (− 538.3, 299.5)	588.8 (161.4, 1016.1) **	708.2 (103.7, 1312.7)	**0.023**
BMC (g)	3.3 (− 5.9, 12.5)	− 0.6 (− 10.0, 8.8)	− 3.9 (− 17.1, 9.4)	0.562
CT scan				
VAT (cm^3^)	− 2.84 (− 5.50, − 0.19)	0.04 (− 2.67, 2.75)	2.88 (− 0.95, 6.72)	0.136
SAT (cm^3^)	− 0.84 (− 4.19, 2.50)	− 3.67 (− 7.09, − 0.26)	− 2.83 (− 7.66, 1.99)	0.243
Liver fat (HU)	0.16 (− 1.23, 1.54)	1.51 (0.09, 2.92) *	1.35 (− 0.65, 3.35)	0.180
Physical activity				
Mean daily activity (mg)	0.76 (− 1.91, 3.43)	− 1.81 (− 4.54, 0.91)	− 2.57 (− 6.43, 1.28)	0.185
Sedentary time (minutes/day)	− 25.77 (− 44.34, − 7.20) **	6.76 (− 12.18, 25.71)	32.53 (5.74, 59.33)	**0.018**
LPA (minutes/day)	10.37 (1.26, 19.48) **	5.05 (− 4.24, 14.34)	− 5.32 (− 18.46, 7.83)	0.419
MVPA (minutes/day)	1.71 (− 7.64, 11.06)	− 7.31 (− 16.85, 2.23)	− 9.02 (− 22.52, 4.47)	0.185
Energy balance				
Energy expenditure (MJ/day)	− 0.09 (− 0.47, 0.29)	− 0.23 (− 0.61, 0.16)	− 0.14 (− 0.68, 0.41)	0.615
Energy intake (MJ/day)	0.10 (− 0.81, 1.00)	− 0.18 (− 1.10, 0.74)	− 0.28 (− 1.58, 1.03)	0.672

Results are presented as estimated marginal means with 95% confidence intervals for all participants. The between-group *p*-values (high load vs low load) given within the table are calculated using analysis of covariance (ANCOVA) adjusted for age, sex, baseline BMI, vest exposure (h) and standing % with vest. Within-group comparisons (5 weeks vs baseline) were made using Wilcoxon signed-rank test. *P < 0.05, **P < 0.01, ***P < 0.001. Statistically significant differences are highlighted in bold. *BMC* bone mineral content, *BMI* body mass index, *CT* computed tomography, *DXA* dual-energy X-ray absorptiometry, *HU* Hounsfield unit, *Liver fat* estimated as liver attenuation, *LPA* light physical activity, *Mg* milli-g (acceleration), *MJ* megajoules, *MVPA* moderate to vigorous physical activity, *SAT* subcutaneous adipose tissue in the abdominal region, *VAT* visceral adipose tissue in the abdominal region

**Fig. 4:**
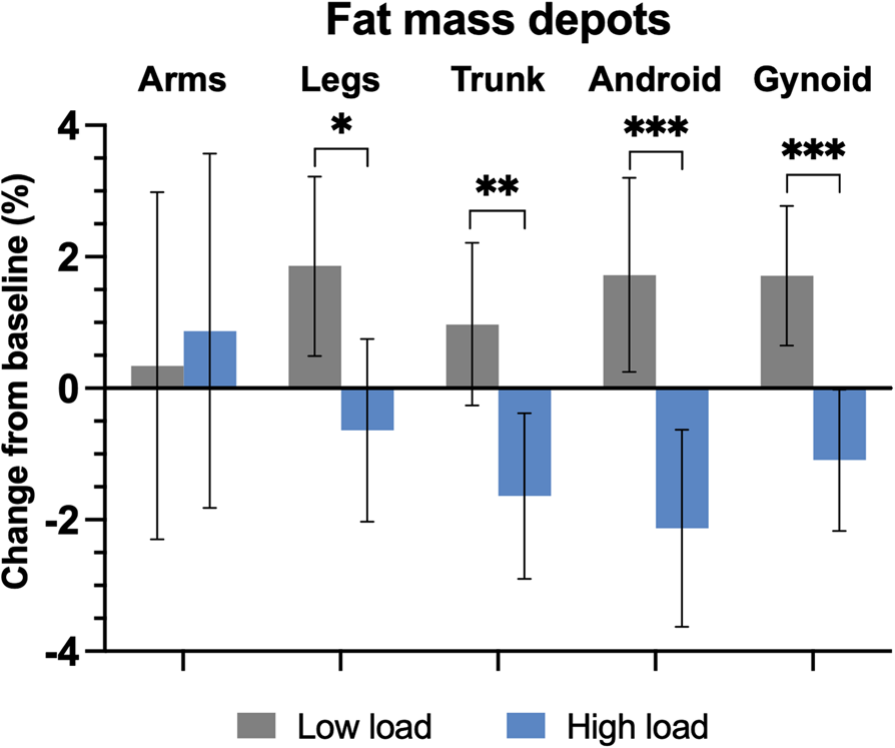
Percentage change in different regions fat mass at 5 weeks vs. baseline for all participants who completed the trial according to protocol. Participants treated with light weight vest (low load; *n* = 26) or heavy weight vest (high load, *n* = 25). Body composition measured with dual-energy X-ray absorptiometry (DXA). Results are presented as estimated marginal means with error bars showing 95% confidence intervals. The between-group *P*-values (high load vs low load) are calculated using analysis of covariance (ANCOVA) adjusted for age, sex, baseline body mass index (BMI), vest exposure (h) and standing % with vest. *P < 0.05, **P < 0.01, ***P < 0.001

Analysis of fat mass distribution revealed significant reductions in fat mass in weight-bearing regions, including the legs, trunk, android and the gynoid regions, in the high-load group compared to the low-load group; but no change was observed in the non-weight-bearing regions of the arms (Fig. 4; Additional file 3: Table S4). In addition, waist circumference was reduced by 2.3% (95% CI − 3.81, − 0.71) or 2.44 cm (95% CI − 4.16, − 0.72) in the high-load group compared to the low-load group (Table 2; Additional file 3: Table S3).

Statistical analyses demonstrated that there was no interaction effect from ANCOVA regarding gender for increased loading on body weight, fat mass, lean mass or waist circumference (*P* > 0.05 for the gender interaction terms).

Abdominal CT analysis did not identify any statistically significant between-treatment group differences for abdominal subcutaneous fat, visceral fat or liver fat. Within-group analyses revealed that the high-load treatment, but not low-load treatment, increased liver attenuation with 1.5 Hounsfield units (95% CI 0.09, 2.92; Table 2) indicating less liver steatosis.

### Effects of high-load treatment on physical activity, energy balance and metabolic markers

Mechanistic analyses using tri-axial accelerometers revealed that the high-load group slightly increased their sedentary time during the treatment (non-significant), while the low-load group decreased theirs. This resulted in a significant difference of 4.7% (95% CI 0.98, 8.39; *P* = 0.014) or 32.5 min per day (95% CI 5.7, 59.3; *P* = 0.018) between groups (Table 2; Additional file 3: Table S3). No significant differences between the treatment groups were observed for energy expenditure, as measured using doubly labelled water, or energy intake (Table 2; Additional file 3: Table S3). Exploratory analyses of metabolic markers in serum and plasma did not reveal any treatment-related differences in total cholesterol, HDL cholesterol, LDL cholesterol, triglycerides, glycerol, glucose, insulin, leptin or calculated HOMA-IR index (Additional file 3: Table S5).

### Adverse events and safety

No treatment-related serious adverse event was reported during the study. The high-load group had in total 29 treatment-related adverse events compared to 6 in the low-load group (*P* < 0.001; Additional file 3: Table S6). Most treatment-related adverse events were musculoskeletal of which none led to discontinuation of any participant from the study. Additionally, the frequency of acute upper respiratory infections was higher in the high-load group compared to the low-load group (Additional file 3: Table S6). All adverse events, medical history and concomitant medications are presented in the supplementary materials (Additional file 3: Table S6, Table S7, Table S8).

## Discussion

Herein, we demonstrate that 5 weeks of treatment with increased weight-loading, applied by weight vests, reduced fat mass and waist circumference while increasing lean mass, this without significant effect on body weight. The high-load treatment led to a healthier body composition with reduced fat mass and an increase in total body lean mass. These beneficial effects occurred despite an increase in sedentary time in the high-load group compared to the low-load group, and in the absence of significant effects on total body energy balance.

An important finding in the present study is that the high-load treatment reduced abdominal fat mass, as demonstrated by the decreased fat mass in the trunk region, determined by DXA, and supported by the associated 2.4 cm reduction in waist circumference. There is compelling evidence that reductions of the truncal fat mass and waist circumference are associated with lower morbidity and mortality rates [16, 41], strongly suggesting that the high-load treatment in the present trial improved metabolic health. The abdominal CT analyses revealed that the high-load group, but not the low-load group, showed increased liver attenuation, suggesting that weight-loading may reduce liver steatosis. However, this observation was not accompanied by a statistically significant between-group difference for liver fat, highlighting the need for confirmation in independent studies. Similarly, abdominal CT analyses did not identify any statistically significant between-group differences for VAT or SAT volumes. The discrepancy between the lack of effect on abdominal adipose tissue volumes as determined by CT and the reductions observed in truncal fat by DXA and waist circumference may be due to differences in the anatomical regions assessed by these methods. The relatively small volumes measured with CT at a single abdominal level (L3–L4) may increase the likelihood of missing significant changes in overall abdominal adipose tissue. In contrast, DXA provides a more comprehensive assessment of total trunk fat mass, offering a broader view of regional fat changes. Furthermore, CT scans measured only VAT and SAT, whereas DXA assessed total trunk fat mass without differentiating between fat types [42]. While the reduction in waist circumference likely reflects a loss of subcutaneous fat (albeit not significantly different between groups), it is possible that changes in smaller fat depots not captured by CT, such as IMAT, may also have contributed to the differences observed between DXA and CT. However, no definitive explanation can be provided based on the available data, and this warrants further investigation.

In the present study, the physical activity and sedentary time were determined using thorough and validated tri-axial accelerometers [38, 43, 44]. Sedentary time increased by 32.5 min per day in the high-load treatment group compared to the low-load group, primarily due to a within-group reduction in sedentary time and an increase in light physical activity in the low-load group. The increased sedentary time observed between groups may be related to discomfort from the heavier vest and a higher incidence of musculoskeletal side effects in the high-load group. All participants were instructed to increase their time spent standing, which is typically associated with increased physical activity. However, no between-group differences in standing time were observed based on participants’ daily records. It is possible that the high-load group adhered to the standing instructions to a lesser degree than the low-load group, which may have contributed to the observed differences in physical activity. These differences in physical activity levels could have attenuated some of the beneficial effects of increased weight-loading on fat mass and its distribution. Despite these challenges, it is noteworthy that the high-load treatment still demonstrated improvements in body composition, including reductions in fat mass and waist circumference, even in the absence of increased physical activity. It should be noted that accelerometer-based assessments of physical activity levels have limitations, including potential misclassification of activity intensity and an inability to fully capture certain types of movement such as cycling or resistance training. Additionally, the high-load group reported a higher incidence of upper respiratory tract infections compared to the low-load group which may have affected overall activity levels during the intervention. While the cause for this is unclear, it is possible that carrying a heavy vest induced physiological or psychological stress, which may have influenced immune function. Previous studies have linked stress and elevated cortisol to increased susceptibility to infections [45, 46]. This potential connection between weight-loading, stress and susceptibility to infections warrants further investigation. Despite these challenges, our findings underscore that increased weight-loading reduced fat mass and generated a healthier body composition, independent of physical activity changes and despite higher incidence of respiratory infections.

Despite the beneficial changes in body composition, there was no difference in the daily total energy expenditure (TEE) or reported energy intake in the present study. TEE is strongly related to fat-free mass, and the two largest components of TEE are basal metabolic rate (BMR) and activity-induced physical activity [47, 48]. It has previously been demonstrated that increased physical activity does not affect energy balance due to a compensatory increase in energy intake [49]. Furthermore, it has been shown that when individuals exercise more, TEE does not increase steadily and that individuals tend to adapt metabolically to increased physical activity by a compensatory reduction of other daily physical activities [47, 50]. These findings agree with what we observed in the present study with increased sedentary time in the high-load group compared to the low-load group that may in part explain why we did not observe an increased TEE in the high-load treatment group. We did not observe any significant effect of increased loading on reported food intake. As the variations are large and the reliability of self-reported data is low for food intake, we believe that the present negative findings on food intake should be further evaluated in larger studies using objectively determined food intake data.

In this study, the fat mass-reducing effect of increased loading was observed primarily in weight-bearing regions, such as the legs and trunk but not in the arms. This may reflect a higher muscular workload in these regions to support posture and movement during vest use. The increased workload in the high-load group may also explain the observed increases in lean mass in the abdominal region since exercise is known to induce muscle hypertrophy [51, 52]. Interestingly, no between-group differences were observed in leg lean mass, despite the expectation that high-load treatment would increase workload in the legs. This discrepancy could be attributed to the increased sedentary time in the high-load group, as trunk muscles remain active even while sitting, whereas leg muscles are primarily activated during movement. The mechanism behind the tissue-specific effect of exercise training on fat mass is unclear. While some studies suggest that exercise-induced fat loss may be either systemic or regional [53–55], evidence for regional effects on fat mass and lean mass from standing alone remains inconclusive [56, 57]. It is possible that the effects shown in the present study, particularly the increase in lean mass, are mainly caused by increased muscular workload. However, increased weight-loading may also elevate regional energy consumption in load-bearing tissues, independent of muscle activity, suggesting a potential loading-dependent mechanism affecting localised fat loss [58].

It has been proposed that the human body may sense changes in mass or gravitational forces in order to regulate fat mass and maintain a body weight that is optimal for functioning within Earth’s gravitational field. This regulation may occur independently of leptin signaling [28, 29, 59, 60]. The mechanisms underlying this proposed homeostatic regulation of body weight and fat mass remain to be determined but may involve gravitational sensing and neuronal signaling pathways [28, 61]. In the present human study with a duration of 5 weeks, fat mass was reduced by increased loading, while body weight was not significantly reduced as the effect on body weight was partly counteracted by an increased lean mass. This finding indicates that the balance between fat mass and lean mass is regulated by increased loading in humans. Obesity treatment has advanced significantly with the introduction of long-acting GLP-1 receptor agonists [62, 63], which have led to substantial reductions in both body weight and fat mass. However, these pharmacological treatments are also associated with a loss of lean mass, which may be particularly problematic in individuals with sarcopenic obesity [7, 9, 64]. Based on our present findings, we propose that weight-loading may be useful as an addition to GLP-1-based therapies in patients with sarcopenic obesity—not only to enhance fat loss, but also to preserve or even increase lean mass. That said, although the current loading protocol improved body composition, it will require refinement to reduce the risk of musculoskeletal adverse events. A more gradual, progressive increase in loading and/or duration of wear time may help reduce physical strain and support long-term adherence.

The present study has several strengths, such as the randomised design with a predefined analysis plan and rigorous design following IHC guidelines. The main outcomes were measured using gold standard techniques such as DXA and abdominal CT of fat mass distribution, doubly labelled water of total energy expenditure and tri-axial accelerometers of physical activity and sedentary time. The control (low-load) group also wore an identical weight vest but with less weight added compared to the weight vest in the high-load group. However, a limitation is that blinding was not possible because both the investigators and the participants could feel the weight of the vests. Another limitation is that the daily time of using the weight vest and the time using the weight vest standing were self-reported and relatively short, especially the time with the vest in standing position. Additionally, a limitation is the timing of the DXA-scans which were performed in the evenings following a 3-h fasting protocol, instead of in the morning after an overnight fast [65]. The method used to collect and process the accelerometer data could underestimate physical activity outcomes and should therefore be interpreted with caution. Finally, the present study only evaluated the effect of increased weight-loading in participants with mild obesity (BMI 30–34.9 kg/m^2^). Therefore, further studies should also evaluate individuals with severe obesity (BMI ≥ 35 kg/m^2^), overweight (BMI 25–29.9 kg/m^2^) and normal weight (BMI ≤ 24.9 kg/m^2^) separately.

## Conclusions

In conclusion, increased weight-loading reduces fat mass and increases lean mass in individuals with obesity, providing a healthier body composition with reduced waist circumference. These beneficial effects were achieved despite increased sedentary time, a higher incidence of musculoskeletal side effects and a higher incidence of respiratory tract infections in the high-load group compared to the low-load group. The effect on fat mass was most pronounced in tissues exposed to increased loading, implying regional fat loss possibly due to locally increased energy consumption. Similarly, increases in lean mass were localised to regions exposed to weight-loading. Future clinical studies should investigate the mechanism underlying these localised effects, including possible local effects on energy expenditure in tissues exposed to loading. Additionally, exploring how increased weight-loading treatment interacts with other lifestyle changes, such as increased exercise or dietary modifications, could help identify new treatments strategies to reduce fat mass, improve body composition and promote cardiometabolic health.

## Supplementary Information

Additional file 1. CONSORT checklist.

Additional file 2. Study protocol.

Additional file 3: Supplementary information. Supplementary methods. Body weight and body composition. Abdominal adipose tissue. Energy expenditure. Physical activity. Blood sampling. Statistical analysis. Supplementary tables. Table S1 – Results: relative changes. Table S2 – Results: absolute changes. Table S3 – Results: relative changes. Table S4 – Results: absolute regional body composition changes. Table S5 – Results: serum/plasma markers. Table S6 – Reported adverse events and serious adverse events. Table S7 – Medical history at baseline for all randomised participants. Table S8 – Concomitant medications.

## Data Availability

Restrictions apply to the availability of the data generated or analysed during this study to preserve patient confidentiality. The corresponding author will on request detail the restrictions and any conditions under which access to some data may be provided. Study protocol and statistical analysis plan will be available with this publication.

## Abbreviations

ANCOVA: Analysis of covariance
ATLAS: Anti-obesity Treatment by Loading in Adult Subjects
BMC: Bone mineral content
BMI: Body mass index
BMR: Basal metabolic rate
CI: Confidence interval
CONSORT: Consolidated Standards of Reporting Trials
CT: Computed tomography
DLW: Doubly labelled water
DXA: Dual-energy X-ray absorptiometry
ECG: Electrocardiography
EE: Energy expenditure
eCRF: Electronic case report form
FAS: Full analysis set
GCP: Good clinical practice
HDL: High-density lipoprotein
HOMA-IR: Homeostatic model assessment for insulin resistance
HU: Hounsfield Units
ICH: International Conference on Harmonization Guidelines
IMAT: Intermuscular adipose tissue
LDL: Low-density lipoprotein
LPA: Light physical activity
MET: Metabolic equivalents of task
Mg: Millig-unit (acceleration)
MJ: Megajoule
MPA: Moderate physical activity
MVPA: Moderate-to-vigorous physical activity
PA: Physical activity
PP: Per protocol
RCT: Randomised controlled trial
SAE: Severe adverse event
SAT: Subcutaneous adipose tissue
SD: Standard deviation
SDQ: Short Dietary Questionnaire
SED: Sedentary time
TEE: Total energy expenditure
VAT: Visceral adipose tissue
VPA: Vigorous physical activity
VVPA: Very vigorous physical activity
