# Supplementary material for: Increased weight-load improves body composition by reducing fat mass and waist circumference, and by increasing lean mass in participants with obesity: a single-centre randomised controlled trial
Source: BMC Med. 2025 May 30;23:317. doi: 10.1186/s12916-025-04143-6 (PMC12123769; doi:10.1186/s12916-025-04143-6)
Supplement: Supplementary file 2 — Additional file 2. Study protocol. [file 12916_2025_4143_MOESM2_ESM.pdf]

CLINICAL STUDY PROTOCOL

Effect of artificial loading, through application of weight vests, on body weight in obese subjects; a randomized controlled trial

**Official Title:**	Effect of artificial loading, through application of weight vests, on body weight in obese subjects; a randomized controlled trial
**Study Code:**	Anti-obesity Treatment by Loading in Adult Subjects (ATLAS)
**Study Acronym:**	ATLAS
**ClinicalTrials.gov Identifier (NCT):**	NCT04697238
**Version Number:**	14.9
**Date:**	2021-02-01
**Sponsor:**	Claes Ohlsson, MD, PhD, Professor and Senior Consultant<br>Sahlgrenska University Hospital, Gothenburg, Västra Götalandsregionen
**Principal investigator:**	Dan Curiac, MD<br>Senior Physician<br>Clinical Trial Centre,<br>Principle Investigator
**Investigators**	Per-Anders Jansson, MD, PhD, Professor and Senior Consultant,<br>Wallenberg Laboratory, Dep of Molecular and Clinical Medicine, Institute of Medicine, University of Gothenburg<br><br>John-Olov Jansson, MD, PhD, Professor,<br>Sahlgrenska Academy, University of Gothenburg<br><br>Jakob Bellman, MD<br>Sahlgrenska Academy, University of Gothenburg
**Monitor**	Gothia Forum, Sahlgrenska University Hospital

Table of Contents

SIGNATURE PAGE 5
CONTACT INFORMATION 6
LIST OF USED ACRONYMS AND ABBREVIATIONS 7
1. SYNOPSIS 9
2. INTRODUCTION 17
2.1. Background 17
2.2. Rationale for conducting this study 18
2.3. Risk/Benefit evaluation 19
3. STUDY OBJECTIVES AND ENDPOINTS 20
3.1. Primary and secondary objective(s) 20
3.2. Primary and secondary endpoint(s) 20
4. STUDY DESIGN AND PROCEDURES 24
4.1. Overall study design 24
4.2. Procedures and flow chart 28
4.3. Rationale for study design 29
4.4. Study visits 29
4.4.1. Visit 0: Pre-screening 29
4.4.2. Visit 1: Day -21 – Screening 29
4.4.3. Visit 2: Day -17 – Distribution of DLW material 29
4.4.4. Visit 3: Day -14 – First DLW measurements 30
4.4.5. Visit 4: Day -13 – Baseline measurements 30
4.4.6. Visit 5: Day -7 – Phone visit 31
4.4.7. Visit 6: Day 0 – Randomization and start of intervention 32
4.4.8. Visit 7: Day 7 – Phone visit 33
4.4.9. Visit 8: Day 14 – Second DLW measurements 33
4.4.10. Visit 9: Day 15 – Second DLW measurements continued 33
4.4.11. Visit 10: Day 21 – Phone visit 35
4.4.12. Visit 11: Day 28 – End of second DLW measurement 35
4.4.13. Visit 12: Day 35 – End of intervention 36
4.4.14. Visit 13: Day 49 – Follow up visit 36
4.5. Biological sampling procedures 37
4.5.1. Handling, storage and destruction of biological samples 37
4.5.2. Total volume of blood per subject 38
4.5.3. Total volume of urine per subject 38
4.5.4. Biobank 38
4.6. End of study 38
5. STUDY POPULATION 39
5.1. Inclusion criteria 39
5.2. Exclusion criteria 39
5.3. Restrictions 40
5.4. Subject enrolment and randomization 41
5.5. Withdrawal criteria 41
5.5.1. Premature termination of the study 42
5.6. Re-screening 42
6. STUDY TREATMENTS 42

6.1. RANDOMIZATION ... 42
6.2. CONCOMITANT MEDICATIONS ... 42

7. METHODS FOR MEASUREMENTS OF ENDPOINTS ... 42

7.1. METHODS FOR MEASUREMENT OF ENDPOINTS FOR CLINICAL EFFICACY ... 42
7.1.1. Methods for primary endpoint ... 42
7.1.2. Methods for secondary endpoint(s) ... 43
7.2. METHODS FOR MEASUREMENT OF ENDPOINTS FOR CLINICAL SAFETY ... 48

8. SAFETY ... 49

8.1. DEFINITIONS ADVERSE EVENTS ... 49
8.1.1. Adverse events (AE) ... 49
8.1.2. Serious Adverse events (SAE) ... 49
8.2. ASSESSMENT OF ADVERSE EVENTS ... 50
8.2.1. Assessment of casual relationship ... 50
8.2.2. Assessment of intensity ... 50
8.2.3. Assessment of seriousness ... 50
8.3. REPORTING AND REGISTRATION OF ADVERSE EVENTS ... 51
8.3.1. Reporting of Adverse Events (AE) ... 51
8.3.2. Reporting of Serious Adverse Events (SAE) ... 51
8.4. FOLLOW-UP OF ADVERSE EVENTS ... 51
8.5. PROCEDURES IN CASE OF EMERGENCIES OR PREGNANCY ... 51

9. STATISTICS ... 52

9.1. ANALYSIS POPULATION ... 52
9.2. STATISTICAL ANALYSES ... 52
9.2.1. Statistical methods ... 52
9.2.2. Drop-outs ... 53
9.3. SAMPLE SIZE CALCULATION ... 53

10. QUALITY CONTROL AND QUALITY ASSURANCE ... 54

10.1. QUALITY ASSURANCE AND OVERSIGHT ... 54
10.2. MONITORING ... 54
10.3. SOURCE DATA ... 54
10.4. DEVIATIONS OR SERIOUS BREACHES ... 55
10.5. AUDITS AND INSPECTIONS ... 55

11. ETHICS ... 55

11.1. COMPLIANCE TO THE PROTOCOL, GCP AND REGULATIONS ... 55
11.2. ETHICAL REVIEW OF THE STUDY ... 55
11.3. PROCEDURE FOR OBTAINING INFORMED CONSENT ... 55
11.4. SUBJECT DATA PROTECTION ... 56
11.5. INSURANCES ... 56

12. PROTOCOL DEVIATIONS AND AMENDMENTS ... 56

13. DATA MANAGEMENT ... 56

13.1. RECORDING OF DATA ... 56
13.1.1. Source data ... 57
13.2. DATA STORAGE AND MANAGEMENT ... 57
13.3. CASE REPORT FORM (FORSKNINGSPERSONSFORMULÄR) ... 57

14. NOTIFICATION OF STUDY COMPLETION, REPORTING AND PUBLICATION ... 57

15. STUDY TIMETABLE **58**
15.1. STUDY PERIOD 58
16. REFERENCES **58**
17. ATTACHMENTS **58**
17.1. SHORT DIETARY QUESTIONNAIRE (SDQ) 58
17.2. PHYSICAL ACTIVITY DIARY 59
17.3. PARTICIPANT INSTRUCTIONS DOUBLY LABELED WATER 59
17.4. WEIGHT VEST DIARY 59

SIGNATURE PAGE

Sponsor

I am responsible for ensuring that this protocol includes all essential information to be able to conduct this study. I will submit the protocol and all other important study-related information to the responsible investigator(s) so that they can conduct the study correctly.

____

Sponsor's signature Date

Claes Ohlsson

____

Printed name

Principal Investigator

I have read this protocol and agree that it includes all essential information to be able to conduct the study. By signing my name below, I agree to conduct the study in compliance with this protocol, the Declaration of Helsinki, ICH GCP (Good Clinical Practice) guidelines and the current national and international regulations governing the conduct of this clinical trial.

I will submit this protocol and all other important study-related information to the staff members and investigators who participate in this study, so that they can conduct the study correctly. I am aware of my responsibility to continuously keep the staff members and investigators who work with this study informed and trained.

I am aware that quality control of this study will be performed in the form of monitoring and possibly audit as well as inspection.

____

Principal Investigator's signature Date

Dan Curiac

____

Printed name

CONTACT INFORMATION

ROLE	NAME	INSTITUTION/SITE	ADRESS	PHONE	MAIL
**SPONSOR**	Claes Ohlsson	Sahlgrenska University Hospital, Institute of Medicine	Vita stråket 11, 413 45 Gothenburg	+46-31-342 2873	claes.ohlsson@medic.gu.se
**PRINCIPAL INVESTIGATOR**	Dan Curiac	Clinical Trial Center, Sahlgrenska University Hospital	Gröna stråket 12, 413 45 Gothenburg	+46700 - 82 33 51	dan.curiac@vgregion.se
**INVESTIGATOR**	Per-Anders Jansson	Sahlgrenska University Hospital, Molecular and Clinical Medicine	Bruna stråket 16, 413 45 Gothenburg	+46702 - 03 30 10	Per-Anders.Jansson@wlab.gu.se
**INVESTIGATOR**	John-Olov Jansson	University of Gothenburg, Department of Physiology	Medicinaregatan 11 41390 Göteborg	+46739-01 11 22	john-olov.jansson@medic.gu.se
**INVESTIGATOR**	Jakob Bellman	University of Gothenburg, Department of Physiology	Medicinaregatan 11 41390 Göteborg	+4673–0427473	jakob.bellman@gu.se
**MONITOR**	Katja Södergren	Gothia Forum	Guldhedsgatan 10C, 413 46 Gothenburg	+46722–016956	katja.sodergren@vgregion.se
**STUDY NURSE**	Helen Svanström	Clinical Trial Center, Sahlgrenska University Hospital	Gröna stråket 12, 413 45 Gothenburg	+4631 – 3428947	helen.svanstrom@vgregion.se

LIST OF USED ACRONYMS AND ABBREVIATIONS

**Abbreviation**	**Term/Explanation**
AE	Adverse Event = any untoward medical occurrence
ANOVA	Analysis of Variance
AR	Adverse Reaction = adverse event, that is each unfavorable and unexpected reaction to a study treatment, regardless of dose
B	Blood
BIA	Bioelectrical Impedance Analysis
BMI	Body Mass Index
CT	Computed Tomography
DSUR	Development Safety Update Report = annual safety report
DXA	Dual energy X-ray Absorptiometry
eCRF	Electronic Case Report Form
EPM	Etikprövningsmyndigheten (English: Swedish Ethical Review Authority)
GCP	Good Clinical Practice
HRV	Heart Rate Variability
HU	Hounsfield Units
ICH	International Council for Harmonization
ITT	Intention-to-treat = including data from all subjects who have been randomized and wore a weight vest at least 2.5 weeks
LVFS	Läkemedelsverkets författningssamling (English: Swedish Medical Products Agency’s statutes)
P	Plasma
PCR	Polymerase Chain Reaction
PI	Principal Investigator
PP	Per Protocol analysis = including only data from subjects who have completed the study completely in accordance with the protocol, with no deviations from the protocol
S	Serum
SAE	Serious Adverse Event = serious untoward medical occurrence
SDQ	Short Dietary Questionnaire
SPC or SmPC	Summary of Product Characteristics
SUSAR	Suspected Unexpected Serious Adverse Reaction
BFR	Bild och Funktionsregistret (imaging storage database)

LIST OF APPENDICES

17.1. Short Dietary Questionnaire (SDQ)
17.2. Physical Activity Diary
17.3. Participant instructions Doubly Labeled Water
17.4. Weight Vest Diary

1. SYNOPSIS

ClinicalTrials.gov Identifier (NCT): NCT04697238

Official Title: Effect of artificial loading, through application of weight vests, on body weight in obese subjects; a randomized controlled trial

Swedish Title: Effekt av tyngdbelastning, genom bärande av viktväst, på kroppsvikt hos överviktiga individer: en randomiserad kontrollerad studie

Study Code: Anti-obesity Treatment by Loading in Adult Subjects (ATLAS)

Study Acronym: ATLAS

Study site: The study will be performed at the Clinical Trial Center at Gothia Forum, Sahlgrenska University Hospital, Gothenburg, Sweden

Study period: March 2021 – December 2021 including administrative tasks

Investigators:
Dan Curiac, MD, Senior Physician, Sahlgrenska University hospital, Gothia Forum, Principal Investigator (PI).

Per-Anders Jansson, MD, PhD, Professor and Senior Consultant, Wallenberg Laboratory, Dep of Molecular and Clinical Medicine, Institute of Medicine, University of Gothenburg

John-Olov Jansson, MD, PhD, Professor at Institute of Neuroscience and Physiology, University of Gothenburg

Claes Ohlsson, MD, PhD, Professor at Institute of Medicine, Centre for Bone and Arthritis Research, Sahlgrenska University Hospital

Jakob Bellman, MD, Institute of Neuroscience and Physiology, University of Gothenburg

Rationale for conducting the study: Obesity related ailments, such as cardiovascular diseases (CVD) and metabolic disorders are major causes of death in the Western World. The proposed research may result in improved prevention, diagnosis and treatments of obesity and obesity-related disorders. Our group has discovered evidence for a previously unknown homeostatic body weight regulating system involving osteocytes of the weight-bearing bones, *the gravitostat*, operating in rodents. In a recently published clinical study we have shown that the gravitostat is also functioning in humans; subjects wearing a weight vest for 3 weeks decreased their body weight significantly compared with controls [1].

We aim to further investigate this homeostatic mechanism of body weight regulation in humans in a longer clinical study. We plan to let subjects with obesity carry weight vests and monitor their change in body weight, waist circumference, body composition, visceral fat mass, subcutaneous fat mass and degree of liver fat. We will examine changes in metabolic rate, physical activity and activity of the autonomic nervous system. We will also measure changes in triglycerides and cholesterol levels to further examine the potential beneficial effects of loading. Blood sampling will be performed to investigate the mechanism of action.

Objectives: The primary and secondary objectives are detailed in Table 1.

Table 1.

Nr.	Primary Objective:	Primary Variables/Measures:
1.	To determine if wearing a weight vest with 11 % of body weight for 8 hours/day for 5 weeks decreases body weight in obese subjects.	Change in body weight (in % of baseline body weight) between day 35 and baseline in the high load group compared with low load group.
	**Secondary Objectives:**	**Secondary Variables/Measures:**
2.	To determine if wearing a weight vest with 11 % of body weight for 8 hours/day for 5 weeks decreases absolute body weight in obese subjects.	Change in body weight in absolute values (g) between day 35 and baseline in the high load group compared with low load group.
3.	To determine if wearing a weight vest with 11 % of body weight for 8 hours/day for 15 days decreases body weight in obese subjects.	Change in body weight between day 15 and baseline. Change measured in both absolute values (g) and % of baseline body weight in the high load group compared with low load group.
4.	To determine if wearing a weight vest with 11 % of body weight for 8 hours/day for 4 weeks decreases body weight in obese subjects.	Change in body weight between day 28 and baseline. Change measured in both absolute values (g) and % of baseline body weight in the high load group compared with low load group.
5.	To determine if wearing a weight vest with 11% of body weight for 8 hours/day for 5 weeks affects levels of total abdominal fat in obese subjects.	Change in levels of total abdominal fat between day 35 and baseline. Change measured in both absolute values (cm^2) and % of baseline value from CT images of the abdomen. Changes in the high load group compared with low load group.
6.	To determine if wearing a weight vest with 11% of body weight for 8 hours/day for 5 weeks affects levels of subcutaneous fat in obese subjects.	Change in levels of subcutaneous fat between day 35 and baseline. Change measured in both absolute values (cm^2) and % of baseline value from CT images of the abdomen. Changes in the high load group compared with low load group.
7.	To determine if wearing a weight vest with 11% of body weight for 8 hours/day for 5 weeks affects levels of visceral fat in obese subjects.	Change in levels of visceral fat between day 35 and baseline. Change measured in both absolute values (cm^2) and % of baseline value from CT images of the abdomen. Changes in the high load group compared with low load group.
8.	To determine if wearing a weight vest with 11% of body weight for 8 hours/day for 5 weeks affects ratio of subcutaneous fat to visceral fat in obese subjects.	Change in levels of subcutaneous fat compared to changes in levels of visceral fat between day 35 and baseline. Change measured in both absolute values (cm^2) and % of baseline values from CT images of the abdomen. Ratio in the high load group compared with low load group.

9.	To determine if wearing a weight vest with 11% of body weight for 8 hours/day for 5 weeks affects levels of liver fat in obese subjects.	Change in levels of liver fat between day 35 and baseline. Change measured in both absolute values (Hounsfield Units (HU)) and % of baseline value on CT images of the abdomen. Changes in the high load group compared with low load group.
10.	To determine if wearing a weight vest with 11% of body weight for 8 hours/day for 5 weeks causes changes in topographic CT images.	CT image analysis performed to investigate any differences between day 35 and baseline, for example with respect to change in volume or intensity. Change measured in both absolute and relative values from CT images of the abdomen. Changes in the high load group compared with low load group.
11.	To determine if wearing a weight vest with 11 % of body weight for 8 hours/day for 5 weeks affects fat mass, fat free mass or water mass in obese subjects.	Change in levels of fat mass, fat free mass and water mass between day 35 and baseline. Changes measured in both absolute values (g) and % of baseline value with bioelectrical impedance analysis (BIA) and Dual-energy X-ray absorptiometry (DXA). Water mass only measured using BIA. Changes in the high load group compared with low load group.
12.	To determine if wearing a weight vest with 11 % of body weight for 8 hours/day for 15 days affects fat mass, fat free mass or water mass in obese subjects	Change in levels of fat mass, fat free mass and water mass between day 15 and baseline. Changes measured in both absolute values (g) and % of baseline value with bioelectrical impedance analysis (BIA). Changes in the high load group compared with low load group.
13.	To determine if wearing a weight vest with 11 % of body weight for 8 hours/day for 28 days affects fat mass, fat free mass or water mass in obese subjects	Change in levels of fat mass, fat free mass and water mass between day 28 and baseline. Changes measured in both absolute values (g) and % of baseline value with bioelectrical impedance analysis (BIA). Changes in the high load group compared with low load group.
14.	To determine if wearing a weight vest with 11% of body weight for 8 hours/day for 5 weeks affects waist circumference in obese subjects.	Change in waist circumference between day 35 and baseline. Change measured in both absolute value (cm) and % of baseline value using measuring tape. Change in the high load group compared with low load group.
15.	To determine if wearing a weight vest with 11% of body weight for 8 hours/day for 15 days affects waist circumference in obese subjects.	Change in waist circumference between day 15 and baseline. Change measured in both absolute value (cm) and % of baseline value using measuring tape. Change in the high load group compared with low load group.

16.	To determine if wearing a weight vest with 11% of body weight for 8 hours/day for 28 days affects waist circumference in obese subjects.	Change in waist circumference between day 28 and baseline. Change measured in both absolute value (cm) and % of baseline value using measuring tape. Change in the high load group compared with low load group.
17.	To determine if wearing a weight vest with 11 % of body weight for 8 hours/day for 5 weeks affects serum concentrations of circulating proteins, metabolites or electrolytes.	Change in serum concentration of:<br>glucose, HbA1c, insulin, HDL, LDL, TAG, Free Fatty Acids, Glycerol, FGF21, osteocalcin, FGF23, FGF15, sclerostin, lipocalin2, leptin, ghrelin, GLP-1, GLP-2, CCK, (3-36) PYY, glucagon, renin, testosterone, estrogens, CRP, methoxynorepinephrine, Na, K, Cl, Ca or creatinine between day 35 and baseline. Change measured in both absolute values and % of baseline values. Exploratory analysis in serum of other circulating proteins, metabolites or electrolytes may be performed. Analysis in form of proteomics or metabolomics may also be executed. Change in the high load group compared with low load group.
18.	To determine if wearing a weight vest with 11 % of body weight for 8 hours/day for 15 days affects serum concentrations<br>of circulating proteins, metabolites or electrolytes.	Change in serum concentration of for example:<br>glucose, HbA1c, insulin, HDL, LDL, TAG, Free Fatty Acids, Glycerol, FGF21, osteocalcin, FGF23, FGF15, sclerostin, lipocalin2, leptin, ghrelin, GLP-1, GLP-2, CCK, (3-36) PYY, glucagon, renin, testosterone, estrogens, CRP, methoxynorepinephrine, Na, K, Cl, Ca or creatinine between day 15 and baseline. Change measured in both absolute values and % of baseline values. Exploratory analysis in serum of other circulating proteins, metabolites or electrolytes may be performed. Analysis in form of proteomics or metabolomics may also be executed. Change in the high load group compared with low load group.
19.	To determine if wearing a weight vest with 11% of body weight for 8 hours/day for 3 weeks affects physical activity in obese subjects.	Change in physical activity between day 21 and baseline. Measured as time distribution in a spectrum of physical activity intensities assessed by accelerometer worn for 24 hours a day for one week. Change measured in both absolute value and % of baseline value. Changes compared between measurements during intervention (day 15 to 21) and baseline (day -13 to -7). Change in the high load group compared with low load group.

20.	To determine if wearing a weight vest with 11% of body weight for 8 hours/day for 4 weeks affects energy expenditure in obese subjects.	Change in energy expenditure between day 28 and baseline. Change measured in both absolute value (Joules per day) and % of baseline value using the doubly labeled water method. Changes compared between measurement during intervention (day 14 to 28) and baseline (day -14 to 0). Change in the high load group compared with low load group.
21.	To determine if wearing a weight vest with 11% of body weight for 8 hours/day for 4 weeks affects food intake in obese subjects.	Change in food intake between day 28 and baseline. Change measured in both absolute value (calories) and % of baseline value using the validated questionnaire Short Dietary Questionnaire (SDQ). Changes compared between measurement during intervention (day 14 to 28) and baseline (day -14 to 0). Change in the high load group compared with low load group.
22.	To determine if wearing a weight vest with 11 % of body weight for 8 hours/day for 2 weeks affects autonomic nervous system activity in obese subjects	Change in heart rate variability (HRV) between day 15 and baseline. Change measured in both absolute values and % of baseline values using 24-hour ECG monitoring. Changes measured at day 15 compared with baseline (day -13). Change in the high load group compared with the low load group.
23.	To determine if waist circumference in obese subjects has changed at follow up visit 2 weeks after the end of intervention.	Change in waist circumference between day 49 and day 35. Change measured in both absolute value (cm) and % of baseline value using measuring tape. Change in the high load group compared with low load group.
24.	To determine if body weight in obese subjects has changed at follow up visit 2 weeks after the end of intervention.	Change in body weight between day 49 and day 35. Change measured in both absolute values (g) and % of baseline body weight in the high load group compared with low load group.
25.	To determine if fat mass, fat free mass or water mass in obese subjects have changed at follow up visit 2 weeks after the end of intervention.	Change in levels of fat mass, fat free mass and water mass between day 49 and day 35. Changes measured in both absolute values (g) and % of baseline value with bioelectrical impedance analysis (BIA). Changes in the high load group compared with low load group.

26.	To determine if there is any change at follow up visit 2 weeks after the end of intervention in serum concentrations of circulating proteins, metabolites or electrolytes.	Change in serum concentration of:<br>glucose, HbA1c, insulin, HDL, LDL, TAG, Free Fatty Acids, Glycerol, FGF21, osteocalcin, FGF23, FGF15, sclerostin, lipocalin2, leptin, ghrelin, GLP-1, GLP-2, CCK, (3-36) PYY, glucagon, renin, testosterone, estrogens, CRP, methoxynorepinephrine, Na, K, Cl, Ca or creatinine between day 49 and day 35. Change measured in both absolute values and % of baseline values. Exploratory analysis in serum of other circulating proteins, metabolites or electrolytes may be performed. Analysis in form of proteomics or metabolomics may also be executed. Change in the high load group compared with low load group.

Study design: Eligible subjects are randomized to wear a heavy weight vest (11% of body weight at randomization) or randomized to wear a light weight vest (1% of body weight at randomization) until we have 25 participants in each group who completed visits at baseline (day 0) and at the end of intervention (day 35).

Study population: Healthy obese subjects with a BMI > 30 and ≤ 35 kg/m^2.

Number of subjects: 50 participants who complete the study, i.e. visits at baseline and day 35.

Inclusion criteria:
For inclusion in the study, subjects must fulfil all of the following criteria:

1. Signed informed consent to participate in the study.
2. Consent out of free will.
3. 18-65 years of age.
4. Obesity as defined by a BMI >30 and ≤35. Fat mass above 25 % [2].
5. Willingness to comply with the study protocol.
6. Normal or clinically non-significant screening of blood samples:
    a. Hemoglobin (Hb), White Blood Cell Count (WBC), thrombocyte count, sodium (Na), potassium (K), chloride (Cl), calcium (Ca), creatinine, aspartate transaminase (AST), alanine transaminase (ALT), alkaline phosphatase (ALP), total bilirubin, Prothrombin Time (PT-INR), Activated Partial Thromboplastin Time (APTT), human immunodeficiency viruses (HIV), Hepatitis B, Hepatitis C, glycated hemoglobin (HbA1c), C-reactive protein (CRP), free thyroxine (fT4), thyroid stimulating hormone (TSH).
    b. Normal or clinically non-significant aberrations of screening blood samples are defined as:
        i. Normal: Values within the reference interval supplied by the local lab at Sahlgrenska University Hospital.

    ii. Clinically non-significant aberration: as judged by investigator (Clinical significance judged by investigator).
7. Normal or non-clinically significant 12-lead electrocardiography (ECG) recording as judged by the investigator.

Exclusion criteria:
Subjects must not be included in the study if any of the following criteria are fulfilled:

1. Chronic disease that could interfere with the participation in the study as judged by the investigator. For example poorly regulated type 1 or type 2 diabetes, cardiovascular disease that effect daily life, pulmonary disease that affects daily life, anemia or malignancy.

2. Chronic pain such as pain that is constant and impairs quality of life as judged by the investigator; for example: significant back, hip and knee pain.

3. Regular consumption of medicine or natural supplements that affect weight, inhibit physical activity or increase the risk of adverse effects as judged by the investigator. The following drugs will not be accepted:
    a. β-blockers, GLP-1-agonists, DPP-IV-inhibitors, SGLT2-inhibitors, sulfonylureas, insulin, orlistat, anti-obesity drugs, antidepressants, bisphosphonates, β2-agonists, oral corticosteroids, diuretics, benzodiazepines, or central nervous system stimulating drugs such as methylphenidate or dextroamphetamine.
    b. Any illegal drugs according to local laws and regulation.

4. Gastric by-pass surgery or equivalent metabolic surgery in the gastrointestinal tract.

5. Reduced mobility.

6. Pregnancy. Females of childbearing potential must confirm to use reliable contraception (intrauterine device, oral contraceptives or condom) and not suspect to be pregnant. Pregnancy test will be taken on all female subjects of fertile age unless permanently sterile, as judged by the investigator. Permanently sterile women can be excluded from the pregnancy test. Permanent sterilization methods include hysterectomy, bilateral salpingectomy and bilateral oophorectomy.

7. Change in body weight of 5 kg or greater during the past 3 months or recently started a strict diet. Also, a greater change in body weight than 3 kg difference between day -21 and day 0 will not be accepted.

8. Use of any illegal drugs according to local regulations or consuming excessive amounts of alcohol, tobacco, nicotine.
    a. Excessive amounts of above-mentioned substances defined as:
        i. Consumption of more than 9 glasses of wine for women, 14 glasses of wine for men (15 cl/glass 11 % alcohol) or equivalent as judged by the investigator during an ordinary week will not be accepted.
        ii. Individuals with a consumption equal or higher than 10 cigarettes or half a packet of snuff per day.

9. Drastic change in lifestyle during the last 3 months; for example a significant change in physical activity, dietary habits, nicotine, alcohol or drug use as judged by the investigator.

10. Apparent risk of not being able to comply with the study protocol for any reason as judged by the investigator.

11. Having participated in a similar study during the last 6 months.

Reasons for exclusion of participants will be reported in the publication.

Investigational product, dosage and administration: The effect of wearing a heavy weight vest (11% of body weight at randomization) for a minimum of 8 hours a day for 5 weeks compared with wearing a light weight vest (1% of body weight at randomization).

Primary outcome variables and examinations: To measure difference in body weight in obese subjects after 5 weeks between a control group and an intervention group. The intervention group wearing a weight vest with 11% of body weight for 8 hours/day and the control group wearing a weight vest with 1 % of body weight for 8 hours/day. Body weight will be measured using a calibrated scale to make certain and reliable measurements.

2. INTRODUCTION

2.1. Background

According to the latest statistics published by the WHO, more than 1.9 billion adults are overweight, and at least 600 million of them are clinically obese. Worldwide obesity has more than doubled since 1980. Obesity related ailments, such as cardiovascular diseases (CVD) and metabolic disorders are major causes of death in the Western World. The proposed research may result in improved prevention, diagnosis and treatments of obesity and obesity-related disorders. [3]

Epidemiologic studies demonstrate that subjects spending much time sitting have increased risk of obesity, diabetes, and cardiovascular diseases. There is even epidemiologic evidence for an association between sitting time and overall mortality. [4] The mechanism for the anti-obesity effect of standing is essentially unknown. Our group has previously published data showing that if a weight is carried by a rodent the animal will lose body weight and get improved glucose metabolism [5]. In a recent clinical study, we showed that the same mechanism for body weight compensatory downregulation, as response to artificial loading, is also present in humans. [1]

A fundamental principle of biology is that internal body functions can be kept relatively constant by feedback regulation [6]. Before our publication [5], there only existed one known mediator of crucial information to the brain about the energy content of fat depots: leptin, discovered by Friedman almost twenty-five years ago. [7, 8] The importance of leptin as an afferent mediator is illustrated by the profound increase in body fat in animals and humans lacking endogenous leptin, and the reversal of this effect by leptin replacement. [9, 10]

These studies demonstrate that leptin is necessary for fat mass homeostasis. However, most patients with obesity have high endogenous leptin levels and do not respond to leptin treatment, indicating that leptin under these circumstances is not sufficient to suppress body fat mass. This has been referred to as leptin resistance. Contrary to leptin, the down-regulation of body weight as a response to loading, through the homeostatic system known as the “gravitostat”, seem to be present in obese subjects; it even seems to be more effective in obese subjects. [11] Additionally, the gravitostat involves a weight sensing mechanism in the osteocytes. It implies that obesity could be viewed as a disturbance in the homeostatic regulation of body weight. The possibility to target the gravitostatic system pharmacologically, after the signalling pathways being further investigated, is a completely new approach to treating obesity (**Figure 1**). [5]

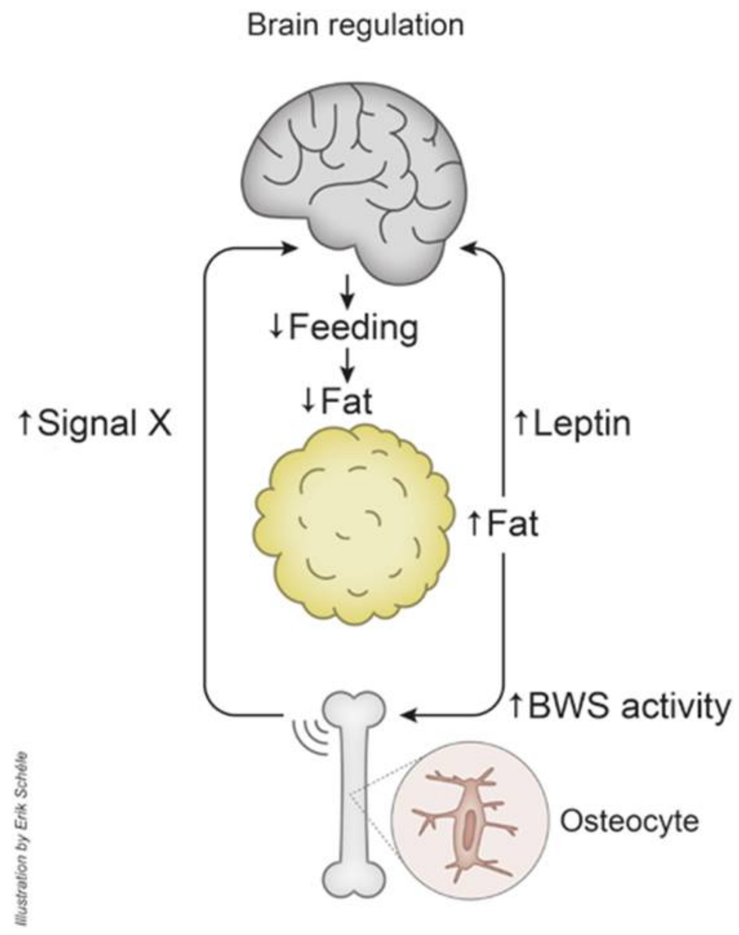

Figure 1. This figure illustrates the two known homeostatic mechanisms of fat regulation: the leptin system and the "gravitostat". Leptin is produced in relation to amount of fat mass and decreases appetite via effects on the brain, possibly the hypothalamus. Leptin is ineffective in obese individuals. An unknown afferent signal (Signal X) from the osteocytes decreases food intake as a response to increased weight. The increased weight is sensed by the osteocytes and increases a activity in a body weight scales (BWS). This recently discovered system is known as the "gravitostat" and it is present in both normal weight and obese rodents. The appetite decreasing signal that is released from the osteocytes is not yet fully elucidated.

2.2. Rationale for conducting this study

We hypothesized that there is a homeostatic regulation of body weight. Results from experiments based on this hypothesis demonstrate that increased loading in rodents reversibly decreases the biological body weight via reduced food intake. The same mechanism has also been shown to be functioning in humans suffering from obesity in a short-term proof of concept clinical study [1]. This novel homeostat for body weight regulates body fat mass independently of fat-derived leptin, revealing two independent negative feed-back systems for fat mass regulation. In rodents we have shown that loading improves glucose tolerance. [5] We now want to investigate if a longer clinical study will result in a more pronounced decrease in body weight and fat mass, and also investigate more specifically if there is a decrease in visceral fat mass. We also want to investigate if loading

for a 5 week period has an effect on metabolic parameters and cardiovascular risk factors in humans.

2.3. Risk/Benefit evaluation

In general, this is a study with few risks. The weight vests used in this experiment are daily used by thousands of people for exercise. During previous experiments on 72 subjects for three weeks no serious adverse events were reported. In the high load group, there was a higher reporting of musculoskeletal adverse events (1 person experienced arthralgia, 2 myalgia, 2 pain in lower extremity, 2 swelling of ankle and/or foot). This is contrasted to 1 person experiencing musculoskeletal adverse events in the low load group (1 myalgia). However, only one subject from the high load group and one subject from the low load group discontinued the study due to musculoskeletal adverse events. It is probable that the musculoskeletal adverse events are due to increased axial loading. However, it should be noted that the risks of wearing a weight vest for several weeks in a row are largely unknown. No serious adverse event was reported in any of the treatment groups. [1]

To minimize the risks we have decided to include only healthy obese subjects consuming no or few medications. The trial will include availability of medically trained personnel, such as physicians and experienced nurses. The trial is to be performed at the Sahlgrenska University Hospital. Study subjects are allowed to withdraw their participation from the study at any time.

Blood sampling can be experienced as somewhat uncomfortable by a few individuals. However, it is generally free from complications. In some subjects, there may be small local bruising or inflammation. We may fail to get venous blood from 5-10% of these obese subjects which may cause some additional suffering and increase the risk of complications.

We plan to perform CT-scans and Dual-energy X-ray absorptiometry (DXA) on two separate occasions for measurement of visceral fat, liver fat and body composition at baseline and after 5 weeks. These procedures will entail exposure to a small dose of radiation. Possible unexpected abnormal findings during the CT- or DXA-scans will be investigated and followed up according to the treatment recommendations at the Sahlgrenska University Hospital.

Energy expenditure will be measured with doubly labeled water which is considered completely safe. [12] The isotopes that are used are deuterium (hydrogen-2) and oxygen-18. Both isotopes are stable, meaning that there is no radiation from the molecules and no risk of decay. Both the hydrogen and oxygen isotope are present in lower concentration in normal drinking water.

24-hour ECG registration will be performed on two occasions during the study. This is a safe and non-invasive method. The ECG will be analyzed by an experienced biomedical analysist and if needed looked on by a cardiologist.

Appropriate measures are taken to minimize risk for Covid-19 infection transmission to participants and personnel at Clinical Trial Center at Gothia Forum.

In summary there are no direct benefits to the study subjects. However, we believe the risks to be very low compared to the potential of exploring a new physiological mechanism; a mechanism with potentially important applications such as new obesity treatments which there is a great need for.

3. STUDY OBJECTIVES AND ENDPOINTS

3.1. Primary and secondary objective(s)

Are described in table 2 under section 3.2.

3.2. Primary and secondary endpoint(s)

Are described below in table 2.

Table 2.

Nr.	**Primary Objective:**	**Primary Variables/Measures:**
1.	To determine if wearing a weight vest with 11 % of body weight for 8 hours/day for 5 weeks decreases body weight in obese subjects.	Change in body weight (in % of baseline body weight) between day 35 and baseline in the high load group compared with low load group.
	**Secondary Objectives:**	**Secondary Variables/Measures:**
2.	To determine if wearing a weight vest with 11 % of body weight for 8 hours/day for 5 weeks decreases absolute body weight in obese subjects.	Change in body weight in absolute values (g) between day 35 and baseline in the high load group compared with low load group.
3.	To determine if wearing a weight vest with 11 % of body weight for 8 hours/day for 15 days decreases body weight in obese subjects.	Change in body weight between day 15 and baseline. Change measured in both absolute values (g) and % of baseline body weight in the high load group compared with low load group.
4.	To determine if wearing a weight vest with 11 % of body weight for 8 hours/day for 4 weeks decreases body weight in obese subjects.	Change in body weight between day 28 and baseline. Change measured in both absolute values (g) and % of baseline body weight in the high load group compared with low load group.
5.	To determine if wearing a weight vest with 11% of body weight for 8 hours/day for 5 weeks affects levels of total abdominal fat in obese subjects.	Change in levels of total abdominal fat between day 35 and baseline. Change measured in both absolute values (cm^2) and % of baseline value from CT images of the abdomen. Changes in the high load group compared with low load group.
6.	To determine if wearing a weight vest with 11% of body weight for 8 hours/day for 5 weeks affects levels of subcutaneous fat in obese subjects.	Change in levels of subcutaneous fat between day 35 and baseline. Change measured in both absolute values (cm^2) and % of baseline value from CT images of the abdomen. Changes in the high load group compared with low load group.
7.	To determine if wearing a weight vest with 11% of body weight for 8 hours/day for 5 weeks affects levels of visceral fat in obese subjects.	Change in levels of visceral fat between day 35 and baseline. Change measured in both absolute values (cm^2) and % of baseline value from CT images of the abdomen. Changes in the high load group compared with low load group.

8.	To determine if wearing a weight vest with 11% of body weight for 8 hours/day for 5 weeks affects ratio of subcutaneous fat to visceral fat in obese subjects.	Change in levels of subcutaneous fat compared to changes in levels of visceral fat between day 35 and baseline. Change measured in both absolute values (cm^2) and % of baseline values from CT images of the abdomen. Ratio in the high load group compared with low load group.
9.	To determine if wearing a weight vest with 11% of body weight for 8 hours/day for 5 weeks affects levels of liver fat in obese subjects.	Change in levels of liver fat between day 35 and baseline. Change measured in both absolute values (Hounsfield Units (HU)) and % of baseline value on CT images of the abdomen. Changes in the high load group compared with low load group.
10.	To determine if wearing a weight vest with 11% of body weight for 8 hours/day for 5 weeks causes changes in topographic CT images.	CT image analysis performed to investigate any differences between day 35 and baseline, for example with respect to change in volume or intensity. Change measured in both absolute and relative values from CT images of the abdomen. Changes in the high load group compared with low load group.
11.	To determine if wearing a weight vest with 11 % of body weight for 8 hours/day for 5 weeks affects fat mass, fat free mass or water mass in obese subjects.	Change in levels of fat mass, fat free mass and water mass between day 35 and baseline. Changes measured in both absolute values (g) and % of baseline value with bioelectrical impedance analysis (BIA) and Dual-energy X-ray absorptiometry (DXA). Water mass only measured using BIA. Changes in the high load group compared with low load group.
12.	To determine if wearing a weight vest with 11 % of body weight for 8 hours/day for 15 days affects fat mass, fat free mass or water mass in obese subjects	Change in levels of fat mass, fat free mass and water mass between day 15 and baseline. Changes measured in both absolute values (g) and % of baseline value with bioelectrical impedance analysis (BIA). Changes in the high load group compared with low load group.
13.	To determine if wearing a weight vest with 11 % of body weight for 8 hours/day for 28 days affects fat mass, fat free mass or water mass in obese subjects	Change in levels of fat mass, fat free mass and water mass between day 28 and baseline. Changes measured in both absolute values (g) and % of baseline value with bioelectrical impedance analysis (BIA). Changes in the high load group compared with low load group.
14.	To determine if wearing a weight vest with 11% of body weight for 8 hours/day for 5 weeks affects waist circumference in obese subjects.	Change in waist circumference between day 35 and baseline. Change measured in both absolute value (cm) and % of baseline value using measuring tape. Change in the high load group compared with low load group.

15.	To determine if wearing a weight vest with 11% of body weight for 8 hours/day for 15 days affects waist circumference in obese subjects.	Change in waist circumference between day 15 and baseline. Change measured in both absolute value (cm) and % of baseline value using measuring tape. Change in the high load group compared with low load group.
16.	To determine if wearing a weight vest with 11% of body weight for 8 hours/day for 28 days affects waist circumference in obese subjects.	Change in waist circumference between day 28 and baseline. Change measured in both absolute value (cm) and % of baseline value using measuring tape. Change in the high load group compared with low load group.
17.	To determine if wearing a weight vest with 11 % of body weight for 8 hours/day for 5 weeks affects serum concentrations of circulating proteins, metabolites or electrolytes.	Change in serum concentration of:<br>glucose, HbA1c, insulin, HDL, LDL, TAG, Free Fatty Acids, Glycerol, FGF21, osteocalcin, FGF23, FGF15, sclerostin, lipocalin2, leptin, ghrelin, GLP-1, GLP-2, CCK, (3-36) PYY, glucagon, renin, testosterone, estrogens, CRP, methoxynorepinephrine, Na, K, Cl, Ca or creatinine between day 35 and baseline. Change measured in both absolute values and % of baseline values. Exploratory analysis in serum of other circulating proteins, metabolites or electrolytes may be performed. Analysis in form of proteomics or metabolomics may also be executed. Change in the high load group compared with low load group.
18.	To determine if wearing a weight vest with 11 % of body weight for 8 hours/day for 15 days affects serum concentrations<br>of circulating proteins, metabolites or electrolytes.	Change in serum concentration of for example:<br>glucose, HbA1c, insulin, HDL, LDL, TAG, Free Fatty Acids, Glycerol, FGF21, osteocalcin, FGF23, FGF15, sclerostin, lipocalin2, leptin, ghrelin, GLP-1, GLP-2, CCK, (3-36) PYY, glucagon, renin, testosterone, estrogens, CRP, methoxynorepinephrine, Na, K, Cl, Ca or creatinine between day 15 and baseline. Change measured in both absolute values and % of baseline values. Exploratory analysis in serum of other circulating proteins, metabolites or electrolytes may be performed. Analysis in form of proteomics or metabolomics may also be executed. Change in the high load group compared with low load group.

19.	To determine if wearing a weight vest with 11% of body weight for 8 hours/day for 3 weeks affects physical activity in obese subjects.	Change in physical activity between day 21 and baseline. Measured as time distribution in a spectrum of physical activity intensities assessed by accelerometer worn for 24 hours a day for one week. Change measured in both absolute value and % of baseline value. Changes compared between measurements during intervention (day 15 to 21) and baseline (day -13 to -7). Change in the high load group compared with low load group.
20.	To determine if wearing a weight vest with 11% of body weight for 8 hours/day for 4 weeks affects energy expenditure in obese subjects.	Change in energy expenditure between day 28 and baseline. Change measured in both absolute value (Joules per day) and % of baseline value using the doubly labeled water method. Changes compared between measurement during intervention (day 14 to 28) and baseline (day -14 to 0). Change in the high load group compared with low load group.
21.	To determine if wearing a weight vest with 11% of body weight for 8 hours/day for 4 weeks affects food intake in obese subjects.	Change in food intake between day 28 and baseline. Change measured in both absolute value (calories) and % of baseline value using the validated questionnaire Short Dietary Questionnaire (SDQ). Changes compared between measurement during intervention (day 14 to 28) and baseline (day -14 to 0). Change in the high load group compared with low load group.
22.	To determine if wearing a weight vest with 11 % of body weight for 8 hours/day for 2 weeks affects autonomic nervous system activity in obese subjects	Change in heart rate variability (HRV) between day 15 and baseline. Change measured in both absolute values and % of baseline values using 24-hour ECG monitoring. Changes measured at day 15 compared with baseline (day -13). Change in the high load group compared with the low load group.
23.	To determine if waist circumference in obese subjects has changed at follow up visit 2 weeks after the end of intervention.	Change in waist circumference between day 49 and day 35. Change measured in both absolute value (cm) and % of baseline value using measuring tape. Change in the high load group compared with low load group.
24.	To determine if body weight in obese subjects has changed at follow up visit 2 weeks after the end of intervention.	Change in body weight between day 49 and day 35. Change measured in both absolute values (g) and % of baseline body weight in the high load group compared with low load group.

25.	To determine if fat mass, fat free mass or water mass in obese subjects have changed at follow up visit 2 weeks after the end of intervention.	Change in levels of fat mass, fat free mass and water mass between day 49 and day 35. Changes measured in both absolute values (g) and % of baseline value with bioelectrical impedance analysis (BIA). Changes in the high load group compared with low load group.
26.	To determine if there is any change at follow up visit 2 weeks after the end of intervention in serum concentrations of circulating proteins, metabolites or electrolytes.	Change in serum concentration of: glucose, HbA1c, insulin, HDL, LDL, TAG, Free Fatty Acids, Glycerol, FGF21, osteocalcin, FGF23, FGF15, sclerostin, lipocalin2, leptin, ghrelin, GLP-1, GLP-2, CCK, (3-36) PYY, glucagon, renin, testosterone, estrogens, CRP, methoxynorepinephrine, Na, K, Cl, Ca or creatinine between day 49 and day 35. Change measured in both absolute values and % of baseline values. Exploratory analysis in serum of other circulating proteins, metabolites or electrolytes may be performed. Analysis in form of proteomics or metabolomics may also be executed. Change in the high load group compared with low load group.

4. STUDY DESIGN AND PROCEDURES

4.1. Overall study design

The principles of the study are shown in **Figure 3**. The aim of the study is to investigate the effect of adding artificial loading to participants with obesity. The weights will be added via a weight vest consisting of 11 % (intervention or high load group) or 1 % (control or low load group) of the participants body weight at randomization; the vest will be worn for 5 weeks and 8 hours per day. Participants will be encouraged to be in a standing position for as many hours as possible of the vest wearing time and are required to be in a standing position at least 2 hours per day while wearing the weight vest. 25 participants will wear a heavy weight vest weighing 11 % of the participants body weight and 25 participants will wear a light weight vest weighing 1 % of the participants body weight. All participants will continue with their normal lifestyle.

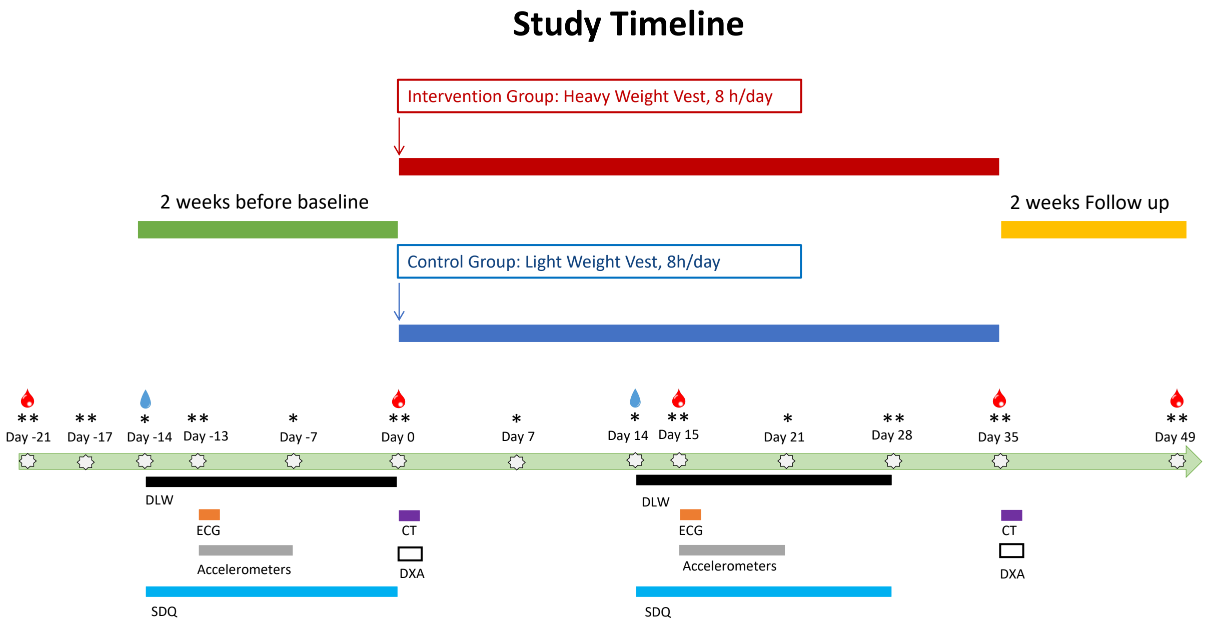

Figure 3. Timeline for study. This figure shows the principles of the study. The dark green line illustrates two weeks before baseline including different measurements. The yellow line represents 2 weeks follow up period. The red line illustrates the intervention group, this group will wear a heavy weight vest with 11 % of body weight for 8 hours a day for 5 weeks. The dark blue line illustrates the control group, this group will wear a light weight vest with 1 % of body weight for 8 hours a day for 5 weeks. Both groups will be instructed to continue with their normal lifestyle during the intervention. The black line represents energy expenditure measurement with doubly labeled water (DLW) which will be performed on day -14 to 0 and on day 14 to 28. For detailed timeline of measurement with doubly labeled water see figure 4. The grey line represents registration of physical activity with accelerometers and a corresponding diary which will be performed on day -13 to -7 and on day 15 to 21. The orange line represents heart rate variability assessment from electrocardiography (ECG) registrations using 24-hour Holter monitoring which will be performed on day -13 and day 15. The purple line represents CT-scan of the abdomen performed on day 0 and day 35. The white line represents DXA-scan performed on day 0 and day 35. The light blue line represents Short Dietary Questionnaire (SDQ) to be registered before baseline and during intervention for evaluation of food intake the last fourteen days.

* Telephone visit for participants. This to prevent mistakes, to document adverse advents and to minimize the loss to follow-up. Visits also to be performed in the evenings on the starting day of the doubly labeled water measurements to ensure compliance to protocol.
** Physical visit for participants at study center.

Blood drop symbol represents blood sampling. Performed on day -21, 0, 15, 35 and 49.

Water drop symbol represents intake of doubly labeled water. Performed in day -14 and 14.

Week -3 – Day -21 (Visit 1): Study center visit. Screening of potential participants. Information to the subject and collecting of written informed consent. Screening blood sampling. Measurements of body weight, height, blood pressure, and body composition.

Week -3 – Day -17 (Visit 2): Study center visit. Enrolled participants will receive information and material for the first day of the first DLW measurement.

Week -2 – Day -14 (Visit 3): Telephone visit. Participants will collect the first urine sample for the first DLW measurement at home and thereafter drink the first dose of DLW. This after an evening telephone visit from study personnel repeating the procedure protocol and explaining any uncertainties.

Week -2 – Day -13 (Visit 4): Study center visit. Enrolled participants will receive equipment and information needed for first measurements with ECG and accelerometers. Collection of the second urine sample for the first DLW measurement. Obtainment of equipment and information needed for the first measurements with DLW. Measurements of body weight and body composition.

Week -1 – Day -7 (Visit 5): Telephone visit.

Week 0 – Day 0 (Visit 6): Study center visit. Fasting blood sampling, CT abdomen, DXA. Measurement of body weight, blood pressure, body composition. Collection of the last urine sample for the first DLW measurement. SDQ registration. Randomization and adding of weight vest. Obtainment of material and information for the first day of the second DLW measurement.

Week 1 – Day 7 (Visit 7): Telephone visit.

Week 2 – Day 14 (Visit 8): Telephone visit. Participants will collect the first urine sample for the second DLW measurement at home and thereafter drink the second dose of DLW. This after an evening telephone visit from study personnel repeating the procedure protocol and explaining any uncertainties.

Week 3 – Day 15 (Visit 9): Study center visit. Fasting blood sampling. Participants will receive equipment and information needed for second measurements with ECG and accelerometers. Collection of the second urine sample for the second DLW measurement. Obtainment of equipment and information needed for the second measurements with DLW. Measurements of body weight, body composition and waist circumference.

Week 3 – Day 21 (Visit 10): Telephone visit.

Week 4 – Day 28 (Visit 11): Study center visit. Collection of the last urine sample for the second DLW measurement. Measurements of body weight, waist circumference and body composition. SDQ registration.

Week 5 – Day 35 (Visit 12): Study center visit. Fasting blood sampling, CT abdomen, DXA. Measurements of body weight, waist circumference, blood pressure and body composition. Removal of weight west.

Week 7 – Day 49 (Visit 13): Study center visit. Fasting blood sampling. Measurements of body weight, blood pressure, body composition and waist circumference.

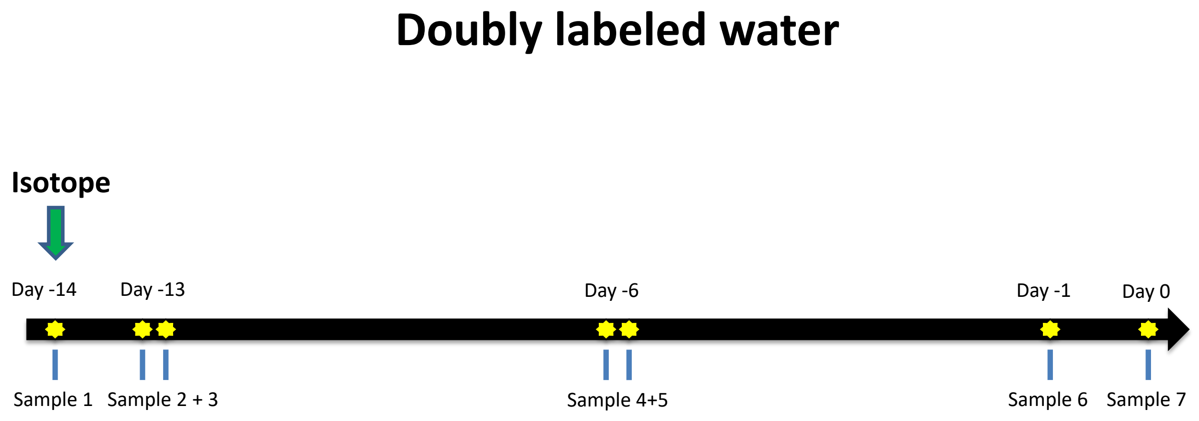

Figure 4. Timeline for measurement with doubly labeled water. On day -14 a urinary sample will be collected as baseline measurement (sample 1). Thereafter the subject will consume a calculated dose of the isotope. Urinary samples will be collected in the morning and evening on day -13 (sample 2 and 3), morning and evening on day -6 (sample 4 and 5), evening of day -1 (sample 6) and morning of day 0 (sample 7). Sample 1, 2 and 7 will be collected at study center or at home.

Body weight, fat mass, water mass and fat free mass will be measured before intervention (day -21, day -13, day 0), during the intervention (day 15, day 28) at the end of intervention (day 35) and at follow up (day 49) to determine the effect of added artificial weights on subjects with obesity.

Fasting blood sampling will be performed at the start of intervention (day 0), during the intervention (day 15), at the end of intervention (day 35) and at follow-up visit (day 49) to further determine the effects of adding artificial weights to obese individuals but also to elucidate the mechanism behind the “gravitostat”. Blood glucose levels will be measured immediately. Blood samples, except blood glucose, will be analyzed either continuous or after all study subjects have been enrolled depending on human resources.

The study participants will report food intake before (day 0) and during (day 28) the intervention. This will be done using the validated questionnaire Short Dietary Questionnaire (SDQ) which will evaluate the food intake the last 2 weeks [13, 14].

Study subjects will be recruited by advertising in the local newspaper, on bulletin boards, social media, websites and with flyers. Potential study subjects will be informed about the hypothesis that people carrying a weight vest may lose body weight.

During the two weeks before baseline, measurements with doubly labeled water, ECG and accelerometers will be performed. The data collected during these two weeks will be used as baseline measurements.

4.2. Procedures and flow chart

Table 2 Study plan detailing the procedures each day

Table 2. This table summarizes all the procedures conducted within the trial.

										Intervention starts											Intervention ends		
**Visit**	**1**		**2**		**3**	**4**		**5**		**6**		**7**		**8**	**9**		**10**		**11**		**12**		**13**
Time point (day)	-21	-20 – -16	-17	-16 – -15	-14	-13	-14 – -7	-7	-7 – 0	0	0 – 7	7	8 – 14	14	15	15 – 21	21	21 – 28	28	28 – 35	35	35 – 49	49
Visiting Window			+/- 3 days		+/- 3 days	See protocol		+/- 3 days		Study center: +/- 3h CT/DXA: +/- 3 days		+/- 3 days		See protocol	See protocol		+/- 3 days		+/- 3 h		+/- 2 days CT/DXA: +/- 3 days		+/- 3 days
Telephone visit					X			X				X		X			X						
Study center visit	X		X			X				X					X				X		X		X
**Study Procedures**																							
Update Adverse events					X	X		X		X		X		X	X		X		X		X		X
Informed Consent	X																						
Information Study Procedures	X		X		X	X		X		X		X		X	X		X		X		X		
Physical examination	X									X					X						X		X
Height for BMI	X																						
Waist circumference										X					X				X		X		X
Body weight	X					X				X					X				X		X		X
Impedance	X									X					X				X		X		X
Fasting Blood Sample										X					X						X		X
Fasting Blood Glucose										X					X						X		X
Screening ECG	X																						
Screening Blood Sample	X																						
Blood pressure	X									X					X						X		X
DXA										X											X		
CT abdomen										X											X		
Reporting Food Intake (SDQ)										X									X				
Reporting Physical Activity						X	X	X							X	X	X						
Measurement with accelerometer						X	X	X							X	X	X						
DLW measurment					X	X	X	X	X	X				X	X	X	X	X	X				
Distribute DLW materials			X			X				X					X								
Urine Sample at study center						X				X					X				X				
Urine Sample at home					X				X					X				X					
24 hour ECG						X									X								

4.3. Rationale for study design

To avoid the risk of carry-over effects, we have decided to not use a crossover design. This could have decreased the variation of the measurement values and increased the power of the study. However, we believe there could be a risk of a carry-over effect with a cross over design. As this is a completely new effect in clinical studies, we have no information about the wash-out time needed to avoid this problem.

4.4. Study visits

4.4.1. Visit 0: Pre-screening

Potential and willing subjects that contact study investigators will in turn be contacted for a pre-screening via a phone call. A brief introduction to the study will be given and information about the inclusion and exclusion criteria. Potential research subjects that are still interested in participating in the study will be booked for a physical screening visit with one of the study investigators.

4.4.2. Visit 1: Day -21 – Screening

Subjects that pass the pre-screening will be booked to a screening visit at the study center. Potential research subjects will receive both oral and written information about the study plan during the visit. They will have time to ask questions and discuss concerns with an investigator. A written informed consent will be obtained from subjects who want to participate in the study. To get enrolled the potential research subject need to meet all the inclusion criteria and must not meet any of the exclusion criteria. Potential subjects will receive an enrolment number at the screening visit obtained from the electronic Case Report Form. During the visit the subject will receive information about all upcoming visits and study procedures in oral and in written form. All subjects will receive a folder containing all study information needed. Visit 2 will be planned and scheduled.

Visit 2, 3 and 4 will be planned and scheduled.

During the visit the following examinations will be performed:

- Physical examination according to section *7.2.1.1 Physical examination.*
- Blood pressure
- Body weight
- Fat mass, water mass and fat free mass will be measured using BIA
- Height measurement for BMI
- Screening blood sample to screen for common diseases that may distort the results and increase the risk of adverse effects. The substances will be measured according to section *7.1.2.6 Blood Samples*.
- In fertile female participants human chorionic gonadotropin (hCG) will be measured according to the inclusion and exclusion criteria. hCG will be measured in urine according to section *7.2.1.3. Pregnancy*.

All above examinations and samples need to be within normal range (or not clinically significant as judged by PI), according to inclusion and exclusion criteria, for a research subject to be included in the study. Subject will be reminded of the following study visit.

4.4.3. Visit 2: Day -17 – Distribution of DLW material

Visiting window: +/- 3 days.

Study center visit. All willing subjects that are eligible according to the inclusion and exclusion criteria will be enrolled in the study. Any questions or uncertainties regarding study procedures will be clarified by study personnel. Any adverse advents will be documented.

Each subject will be thoroughly informed about the protocol for the doubly labeled water (DLW) measurements by study personnel. Subjects will then receive one container for collection of the first urine sample at home and a bottle containing a calculated dose of doubly labeled water. Further detailed written instructions on how to consume the prearranged dose of DLW and how to collect and store the urine sample will also be given.

Visit 3 and 4 will be planned and scheduled. The day before visit 3 subjects will receive a text message to their mobile phone with a reminder regarding visit 3 and the DLW measurements.

4.4.4. Visit 3: Day -14 – First DLW measurements

Visiting window: +/- 3 days.

Telephone visit that shall be performed in the afternoon or evening between 17:00 – 22:00 hours. Study personnel will call subjects and repeat the DLW procedure to be performed by the subjects later the same evening. It is crucial that subjects follow the protocol and they shall first be informed on how to collect the first urine sample (sample 1). Secondly, they shall be informed on how to consume the calculated dose of doubly labeled water for the first DLW measurement. The doubly labeled water should be consumed by the subject as the last consumption of the day. The subject will be reminded of visit 3 at the study center in the morning the following day.

4.4.5. Visit 4: Day -13 – Baseline measurements

Visiting window: Morning visit the day after visit 3.

Study center visit planned in the morning the day after visit 3. The second voiding of the day should be collected at the study center (sample 2) the morning after drinking the doubly labeled water dose. It is important that the first voiding of the day is not collected as sample 2. However, it is not as important if it is the second or third voiding of the day that is collected as sample 2. After sample 2 has been collected body weight shall be measured. Subjects will receive material for collection of urine sample 3-6 at home. Each subject will receive four containers for collection of urine samples and detailed oral and written instructions on how to collect and store the urine samples.

Subjects will receive an accelerometer for measurement of physical activity that will be worn on day -13 to -7. The accelerometer will be attached using a specially designed waist belt and the subject will be instructed how to reattach the device if so to be needed. The subject will be instructed to wear the accelerometer 24 hours per day for seven days. During the meeting a study nurse will attach a portable ECG monitor for a first 24-hour ECG recording. After attachment of the ECG monitor the subject is required to lay down for 1 hour to get a standardized registration, this shall be done at the study center. The subject will then carry the ECG for the following 23-hours during his/her usual activities. The subject will receive two pre-paid envelopes in which the ECG monitor and the accelerometer are to be returned after each registration is completed. Any changes to or new adverse advents will be documented.

During the visit the following examinations will be performed:

- Body weight

- Fat mass, water mass and fat free mass will be measured using BIA

During the visit on day -13 all future visits and study procedures will be planned and scheduled. The participant will receive dates and times for all upcoming visits in oral and written form.

First measurement with DLW and first ECG registration

Urinary samples will be stored by subject in a home freezer after collection. The containers for collection of urine should be dry and have an airtight lock. They should not be rinsed with water before collection of urine since this can disturb the analysis.

Day -14

- On the evening of day -14, before going to bed (usually between 22:00 and 24:00) the subject collects a voiding as a background sample (sample 1) at home.
- Afterwards the subject drinks the bottle with the calculated dose doubly labelled water as the last consumption of the day. The water is consumed straight from the bottle and afterward the bottle is filled with ~50 mL of tap water, closed with a screw cap and shaken, and subsequently the rinsing water is consumed as well.
- Procedures during this day are to be performed after a telephone visit from study personnel informing about the protocol

Day -13

- In the morning the second voiding of this day is collected (sample 2) and the body weight is thereafter measured. This step is to be performed at the study center.
- In the morning an ECG monitor will be attached and subjects are required to lay down to get a standardized registration. This shall be done at the study center.
- In the evening of this day another voiding is collected (sample 3) at home.

Day -6

- An early morning voiding is collected on day -6 (sample 4) at home.
- An evening voiding is collected on day -6 (sample 5) at home.

Day -1

- An evening voiding is collected on day -1 (sample 6) at home.

Day 0

- An early morning voiding is collected on day 0 (sample 7) at the study center.

4.4.6. Visit 5: Day -7 – Phone visit

Visiting window: +/- 3 days.

Visit will be performed as a remote telephone visit by an investigator or a study nurse. Any problems with compliance or other questions regarding the protocol will be discussed.

At this telephone visit the subject will be reminded to detach the accelerometer for measurement of physical activity after a total of one-week registration. Subjects will be reminded to return the accelerometer and ECG using the two pre-paid envelopes previously obtained. Follow up regarding process and collections of urine samples for DLW measurements will be done.

Any changes to or new adverse advents will be documented. Subject will be reminded of the following study visit.

The day before visit 6 subjects will receive a text message to their mobile phone with a reminder regarding fasting blood samples and DLW measurements on visit 6.

4.4.7. Visit 6: Day 0 – Randomization and start of intervention

Visiting window: +/- 3 hours compared to study visit on day -13. CT- and DXA-scans to be performed +/- 3 days from visit 6 on day 0.

Study center visit where subjects will meet with an investigator and a study nurse. At this visit randomization will take place and intervention will start.

The last urine sample (sample 7) will be collected at the study center in the morning. The subject will return the 4 urinary samples collected from the doubly labeled water measurements at home. The accelerometer used for the basal measurement of physical activity and the ECG equipment is also to be returned if not already sent via prepaid envelope. The subject is required to have had an overnight fast before this visit, this due to the blood sampling. Subjects are to answer the first Dietary Short Questionnaire (SDQ) digitally either using their own mobile phone or a specified computer at the study center.

Any changes to or new adverse advents will be documented.

During the visit the following examinations will be performed:

- Physical examination
- Blood pressure
- Body weight
- Fat mass, water mass and fat free mass will be measured using BIA
- Waist circumference
- Fasting venous blood sampling according to section *7.1.2.6. Blood sampling*
- Fasting capillary blood sample for measurement of glucose level according to section *7.1.2.6. Blood sampling*

Subjects are included as they are found to be eligible for inclusion in the study. After the baseline period at day 0 subjects will be randomized in a 1:1 ratio into either the intervention group (heavy weight vest) or the control group (light weight vest). Randomization will be through permuted blocks, stratified by age 18-50 years, age 51-65 years and gender performed in the electronic Case Report Form (eCRF) using a built-in function for the purpose. If a subject discontinues their study participation, their randomization code will not be reused, and the subject will not be allowed to reenter the study again.

Study intervention will start directly after blood sampling and randomization. Subjects will get a randomized three-character code as a randomization number to be used throughout the study. The randomization number will be linked to the subject's enrolment number received at screening. Study subjects will be randomized either to the control or intervention group. The intervention group will obtain a weight vest with 11 % of the subject's body weight (heavy weight vest) and the control group will obtain a weight vest with 1 % of the subject's body weight (light weight vest). The vest is to be worn 8 hours each day for the following 5 weeks. The vest is only to be worn during waking hours. Subjects are also required to be in a standing position for at least 2 hours per day while wearing the vest for the following 5 weeks. Subjects are to be instructed not to wear the vest during upcoming study visits.

Each subject will be informed about the protocol for the second measurement of doubly labeled water (DLW). For the second DLW measurement subjects will then receive one container for collection of the first urine sample at home and a bottle containing a calculated dose of doubly labeled water. Further detailed written instructions on how to consume the prearranged dose of DLW and how to collect and store the urine sample will also be given.

Close to this visit (+/- 3 days) participants will go through a CT- and DXA-scan. This to get baseline values of fat depots, liver fat and body composition. Procedures will be performed by experienced personnel operating the CT- and DXA-scan. Subject will be reminded of the following study visit.

4.4.8. Visit 7: Day 7 – Phone visit

Visiting window: +/- 3 days.

Telephone visit by an investigator or a study nurse. Any problems with compliance will be discussed. At this meeting the subject will also be questioned regarding possible adverse events.

The day before visit 8 subjects will receive a text message to their mobile phone with a reminder regarding visit 8 and the DLW measurements.

Study subjects will continue to wear the vests for 8 hours a day. Subject will be reminded of the following study visit.

4.4.9. Visit 8: Day 14 – Second DLW measurements

Visiting window: Telephone call between 17:00 – 22:00 hours. Must be executed exactly on Day 14 compared to Day 0.

Telephone visit that shall be performed in the afternoon or evening. Study personnel will call subjects and repeat the DLW procedure to be performed by the subjects later the same evening. It is crucial that subjects follow the protocol and they shall first be informed on how to collect the first urine sample (sample 8). Secondly, they shall be informed on how to consume the calculated dose of doubly labeled water for the second DLW measurement. The doubly labeled water should be consumed by the subject as the last consumption of the day. The subject will be reminded of visit 9 at the study center in the morning the following day.

Any changes to or new adverse advents will be documented. Study subjects will continue to wear the vests for 8 hours a day. Subject will be reminded of the following study visit.

The day before visit 9 subjects will receive a text message to their mobile phone with a reminder regarding the DLW measurements and fasting blood samples on visit 9.

4.4.10. Visit 9: Day 15 – Second DLW measurements continued

Visiting window: Morning visit the day after visit 8.

Study center visit planned in the morning the day after visit 8. The second voiding of the day should be collected at the study center (sample 9) the morning after drinking the doubly labeled water dose. It is important that the first voiding of the day is not collected as sample 9. However, it is not as important if it is the second or third voiding of the day that is collected as sample 9. Subjects will receive material for collection of urine sample 10-13 at home. Each subject will

receive four containers for collection of urine samples and detailed oral and written instructions on how to collect and store the urine samples.

Subjects will receive an accelerometer for the second measurement of physical activity that will be worn on day 15 to 21. The accelerometer will be attached by study personnel and the subject will be instructed how to reattach the device if so to be needed. The research subject will be instructed to wear the accelerometer 24 hours per day for seven days. During the visit a study nurse will attach a portable ECG monitor for a second 24-hour ECG recording. After attachment of the ECG the subject is required to lay down for 1 hour to get a standardized registration, this shall be done at the study center. The subject will receive two pre-paid envelopes in which the ECG monitor and the accelerometer are to be returned after each registration is completed. The subject is required to have had an overnight fast before this visit, this due to the blood sampling. Any changes to or new adverse advents will be documented.

The following examinations will be performed:

- Physical examination
- Blood pressure
- Body weight
- Fat mass, water mass and fat free mass will be measured using BIA
- Waist circumference
- Fasting venous blood sampling
- Fasting capillary blood sample for measurement of glucose level

Study subjects will continue to wear the vests for 8 hours each day. Subject will be reminded of the following study visit.

Second measurement with DLW and second ECG registration

Urinary samples will be stored by subject in a home freezer after collection. The containers for collection of urine should be dry and have an airtight lock. They should not be rinsed with water before collection of urine since this can disturb the analysis.

Day 14
- On the evening of day 7, before going to bed (usually between 22:00 and 24:00) the subject collects a voiding as a background sample (sample 8) at home
- Afterwards the subject drinks the bottle with the calculated dose doubly labelled water as the last consumption of the day. The water is consumed straight from the bottle and afterward the bottle is filled with ~50 mL of tap water, closed with a screw cap and shaken, and subsequently the rinsing water is consumed as well.
- Procedures during this day are to be performed after a telephone visit from study personnel informing about the protocol

Day 15
- In the morning the second voiding of this day is collected (sample 9) and the body weight is thereafter measured. This step is to be performed at the study center.
- In the morning an ECG monitor will be attached and subjects are required to lay down to get a standardized registration. This shall be done at the study center.
- In the evening of this day another voiding is collected (sample 10) at home.

Day 22
- An early morning voiding is collected on day -6 (sample 11) at home.
- An evening voiding is collected on day -6 (sample 12) at home.

Day 27
- An evening voiding is collected on day -1 (sample 13) at home.

Day 28
- An early morning voiding is collected on day 0 (sample 14) at the study center.

4.4.11. Visit 10: Day 21 – Phone visit

Visiting window: +/- 3 days.

Visit will be performed as a remote telephone visit by an investigator or a study nurse. Any problems with compliance or other questions regarding the protocol will be discussed.

At this telephone visit the subject will be reminded to detach the accelerometer for measurement of physical activity after a total of one-week registration. Subjects will be reminded to return the accelerometer and ECG using the two pre-paid envelopes previously obtained. Follow up regarding process and collections of urine samples for DLW measurements will be done. Any changes to or new adverse advents will be documented.

Study subjects will continue to wear the vests for 8 hours each day. Subject will be reminded of the following study visit.

4.4.12. Visit 11: Day 28 – End of second DLW measurement

Visiting window: +/- 3 hours. Compared to visit 9 on day 15.

Visit at the study center. The last urine sample (sample 14) will be collected at the study center in the morning. The subject will return the 4 urinary samples collected from the doubly labeled water measurements at home. The accelerometer used for the basal measurement of physical activity and ECG are also to be returned if not already sent via prepaid envelope. Any changes to or new adverse advents will be documented. Any problems with compliance will be discussed. Subjects are to answer the second Dietary Short Questionnaire (SDQ) digitally either using their own mobile phone or a specified computer at the study center.

During the visit the following examinations will be performed:

- Body weight
- Fat mass, water mass and fat free mass will be measured using BIA
- Waist circumference

Study subjects will continue to wear the vests for 8 hours each day. Subject will be reminded of the following study visit. The day before visit 12 subjects will receive a text message to their mobile phone with a reminder regarding the fasting blood samples on visit 12.

4.4.13. Visit 12: Day 35 – End of intervention

Visiting window: +/- 2 days compared to day 0. CT- and DXA-scans to be performed +/- 3 days from visit 12 on day 35.

Subjects will be entitled to another visit with an investigator and a study nurse 5 weeks after the start of intervention. The subject is required to have had an overnight fast before this visit and not have eaten anything before the study visit, this due to the blood sampling. Study subjects will have worn the vests for 8 hours the day before, but they will not wear it during the visit the morning of day 35.

The research subject will be questioned regarding possible adverse events.

The following examinations will be performed:

- Physical examination
- Blood pressure
- Body weight
- Fat mass, water mass and fat free mass will be measured using BIA
- Waist circumference
- Fasting venous blood sampling
- Fasting capillary blood sample for measurement of glucose level

Close to this visit (+/- 3 days) participants will go through a CT- and DXA-scan. Procedures will be performed by experienced personnel operating the CT- and DXA-scan.

The day before visit 13 subjects will receive a text message to their mobile phone with a reminder regarding the fasting blood samples on visit 13.

4.4.14. Visit 13: Day 49 – Follow up visit

Visiting window: +/- 3 days.

Subjects are invited to a follow up visit at the study center 2 weeks after the end of intervention. The subject is required to have had an overnight fast before this visit and not have eaten anything before the study visit, this due to the blood sampling.

During the meeting on study day 49 the following examinations will be performed:
- Physical examination
- Blood pressure
- Body weight
- Fat mass, water mass and fat free mass will be measured using BIA
- Waist circumference
- Fasting venous blood sampling
- Fasting capillary blood sample for measurement of glucose level

At this meeting the subject will also be questioned regarding regression of possible adverse events.

4.5. Biological sampling procedures

4.5.1. Handling, storage and destruction of biological samples

To determine the mechanism of action and elucidate the beneficial effects of body weight loading we will analyze blood samples. Some tubes may be used for analyzes by Sahlgrenska University Hospital local lab and the rest of the tubes will be used for analyzes by the research group at the Institute of Neurophysiology and Physiology, Sahlgrenska Academy, University of Gothenburg. All samples will be pseudonymized and labeled according to a separate log. Measurements will be performed as quickly as possible and samples will then be destroyed. At any time, a study subject can request destruction of his or her blood samples.

Each venous blood drawing consists of approximately 35 ml divided into approximately between 4-10 tubes. The tubes used for blood drawings may contain ethylenediaminetetraacetic acid (EDTA) or a SST-gel for the purpose of manipulating coagulation.

Analysis performed by local lab at Sahlgrenska University Hospital: serum concentration of Hemoglobin (Hb), White Blood Cell Count (WBC), thrombocyte count, sodium (Na), potassium (K), chloride (Cl), calcium (Ca), creatinine, aspartate transaminase (AST), alanine transaminase (ALT), alkaline phosphatase (ALP), total bilirubin, Prothrombin Time (PT-INR), Activated Partial Thromboplastin Time (APTT), human immunodeficiency viruses (HIV), Hepatitis B, Hepatitis C, glycated hemoglobin (HbA1c), C-reactive protein (CRP), free thyroxine (fT4), thyroid stimulating hormone (TSH), insulin, High-density lipoprotein (HDL), Low-density lipoprotein (LDL) and triglycerides.

Fasting blood glucose levels will be measured directly at the study center in capillary blood by using Hemocue® glucose system.

Analysis performed at the Sahlgrenska Academy, University of Gothenburg: Fibroblast Growth Factor 21 (FGF21), FGF23, FGF15, osteocalcin, sclerostin, lipocalin2, leptin, ghrelin, Glucagon-like peptide-1 (GLP-1), GLP-2, CCK, (3-36) PYY, glucagon, testosterone, estrogens, renin, glycerol, free fatty acids (FFA) and methoxynorepinephrine.

Exploratory analysis in serum of circulating proteins, metabolites or electrolytes may be performed. Serum analysis in form of proteomics or metabolomics may also be executed.

Urine samples from the doubly labeled water measurements will be sent to a cooperative research group at Maastricht University in the Netherlands for analysis. All samples will be pseudonymized during the full process and labeled according to a separate log. All samples will be returned to Sahlgrenska University Hospital or destroyed after analysis. Handling and shipment of samples will follow all applying regulations.

All samples will be stored and handled according to the biobank agreement with Biobank Väst (reg. number 890). This has been reported to the Swedish “Health and Social Care Inspectorate” according to regulation 2002:297.

If we experience technical difficulties measuring any of the above mentioned parameters some samples may be sent to another EU/EES country or to other countries internationally for measurements. The samples will then be pseudonymized during the full process. The analysis may take place at a cooperative research group, the industry or drug manufacturer. The samples will be returned or destroyed upon completed analysis. Sending and handling of samples will be according to all applied laws and regulations.

4.5.2. Total volume of blood per subject

Each blood sample will consist of approximately 35 ml blood divided into 4-10 tubes. The total volume of blood taken from each subject during the study is approximately 175 ml. In comparison, a blood donation consists of up to 450 ml.

4.5.3. Total volume of urine per subject

Subjects will collect a total of 14 urine samples divided equally over two doubly labeled water measurements. For each sample the whole voiding will be collected in a plastic container with a total volume of 500-1000 ml. A total of 14 containers will be collected from each subject and the urine volume in each container will depend on the individual voiding volume. At the study center 2 ml of urine will be transferred from each plastic container into two separate glass vials. One of these vials with 2 ml urine will then be sent to Maastricht University, The Netherlands, for analysis. The other vial will be stored according to the biobank agreement. Thus, a total of 56 ml urine will be collected from each subject for analysis. The remaining urine in the plastic containers will be destroyed.

4.5.4. Biobank

All biological samples taken in this study are registered in a biobank according to an agreement with Biobank Väst (registration nr: 890) and handled according to the current biobank laws and regulations (Biobankslagen SFS 2002:297) concerning biobanks in health service and care. The law regulates the way samples can be stored and used as well as regulation about quality and safety for biobanks. The samples will be stored prior to analysis and coded/pseudonymized to protect the identification of the subjects. All samples and the identification/code list are stored securely and separately to prevent unauthorized persons from having access to them. Eventual sending of samples will be done according to current laws and regulations and applied material transfer agreements.

4.6. End of study

The study ends when the last subject has completed the last follow-up, defined as the last visit of the last subject (LVLS). The study may be prematurely terminated if it appears that the treatment involved many serious adverse events (SAE) or if recruitment of subjects cannot be met within

reasonable time limits. If the study is prematurely terminated or suspended, the investigator should immediately inform the subjects about this and ensure appropriate treatment and follow-up. The regulatory authority should be informed as soon as possible, but no later than within 15 days, by the PI, investigator or sponsor.

Decisions on premature termination are taken by the sponsor.

The sponsor, PI or investigator will notify the concerned Ethics Committee and the Competent Authorities at the end of the study within a period of 90 days.

5. STUDY POPULATION

5.1. Inclusion criteria

For inclusion in the study, subjects must fulfil all of the following criteria:

1. Signed informed consent to participate in the study.
2. Consent out of free will.
3. 18-65 years of age.
4. Obesity as defined by a BMI >30 and ≤35. Fat mass above 25 % [2].
5. Willingness to comply with the study protocol.
6. Normal or clinically non-significant screening of blood samples:
    a. Hemoglobin (Hb), White Blood Cell Count (WBC), thrombocyte count, sodium (Na), potassium (K), chloride (Cl), calcium (Ca), creatinine, aspartate transaminase (AST), alanine transaminase (ALT), alkaline phosphatase (ALP), total bilirubin, Prothrombin Time (PT-INR), Activated Partial Thromboplastin Time (APTT), human immunodeficiency viruses (HIV), Hepatitis B, Hepatitis C, glycated hemoglobin (HbA1c), C-reactive protein (CRP), free thyroxine (fT4), thyroid stimulating hormone (TSH).
    b. Normal or clinically non-significant aberrations of screening blood samples are defined as:
        i. Normal: Values within the reference interval supplied by the local lab at Sahlgrenska University Hospital
        ii. Clinically non-significant aberration: as judged by investigator (Clinical significance judged by investigator)
7. Normal or non-clinically significant 12-lead electrocardiography (ECG) recording as judged by the investigator.

5.2. Exclusion criteria

Subjects must not be included in the study if any of the following criteria are fulfilled:

1. Chronic disease that could interfere with the participation in the study as judged by the investigator. For example poorly regulated type 1 or type 2 diabetes, cardiovascular disease that effect daily life, pulmonary disease that affects daily life, anemia or malignancy.

2. Chronic pain such as pain that is constant and impairs quality of life as judged by the investigator; for example: significant back, hip and knee pain.

3. Regular consumption of medicine or natural supplements that affect weight, inhibit physical activity or increase the risk of adverse effects as judged by the investigator. The following drugs will not be accepted:
    a. β-blockers, GLP-1-agonists, DPP-IV-inhibitors, SGLT2-inhibitors, sulfonylureas, insulin, orlistat, anti-obesity drugs, antidepressants, bisphosphonates, β2-agonists, oral corticosteroids, diuretics, benzodiazepines, or central nervous system stimulating drugs such as methylphenidate or dextroamphetamine.
    b. Any illegal drugs according to local laws and regulation

4. Gastric by-pass surgery or equivalent metabolic surgery in the gastrointestinal tract.

5. Reduced mobility.

6. Pregnancy. Females of childbearing potential must confirm to use reliable contraception (intrauterine device, oral contraceptives or condom) and not suspect to be pregnant. Pregnancy test will be taken on all female subjects of fertile age unless permanently sterile, as judged by the investigator. Permanently sterile women can be excluded from the pregnancy test. Permanent sterilization methods include hysterectomy, bilateral salpingectomy and bilateral oophorectomy.

7. Change in body weight of 5 kg or greater during the past 3 months or recently started a strict diet. Also, a greater change in body weight than 3 kg difference between day -21 and day 0 will not be accepted.

8. Use of any illegal drugs according to local regulations or consuming excessive amounts of alcohol, tobacco, nicotine.
    a. Excessive amounts of above-mentioned substances defined as:
        i. Consumption of more than 9 glasses of wine for women, 14 glasses of wine for men (15 cl/glass 11 % alcohol) or equivalent as judged by the investigator during an ordinary week will not be accepted.
        ii. Individuals with a consumption equal or higher than 10 cigarettes or half a packet of snuff per day.

9. Drastic change in lifestyle during the last 3 months; for example a significant change in physical activity, dietary habits, nicotine, alcohol or drug use as judged by the investigator.

10. Apparent risk of not being able to comply with the study protocol for any reason as judged by the investigator.

11. Having participated in a similar study during the last 6 months.

Reasons for exclusion of participants will be reported in the publication.

5.3. Restrictions

The study subjects will be required to wear the weight vests for 8 hours per day. It is allowed to wear the vest in intervals as long as a total wearing time of 8 hours per day is achieved. Subjects who deviate more than 20 % from this (i.e. use the vest for less than 6.4 hours during the daily 8-

hour vest wearing period) will be excluded from the study. Deviation from vest wearing time will be calculated as a mean of the total vest wearing time for all 5 intervention weeks, this at the end of the intervention (day 35).

The study subjects are required to be in a standing position a minimum of 2 hours per day while wearing the weight vest. Subjects who deviate more than 20 % from this (i.e. standing less than 96 minutes during the daily 8-hour vest wearing period) will be excluded from the study. Deviation from standing time will be calculated as a mean of the total standing time for all 5 intervention weeks, this at the end of the intervention (day 35). Subjects will also be encouraged to be in a standing position as many hours possible of the vest wearing time.

The study subjects are only allowed to consume a limited amount of alcohol and other drugs during the study. This in line with the inclusion- and exclusion criteria.

5.4. Subject enrolment and randomization

Healthy subjects will be recruited by advertising in a local newspaper, on social media, on websites, on bulletin boards or with flyers. Only healthy subjects will be enrolled. Each study subject will be given an enrollment number (E-number) at screening. Subjects eligible for randomization will be randomized either to wear heavy weight vest (intervention/high load group) or to wear a light weight vest (control/low load group). Subjects will at randomization receive a randomization number linked to the enrollment number. We will aim to recruit participants until 25 participants in the heavy weight vest group and 25 participants in the light weight vest group have completed the study, i.e. performed visits at day 0 and day 35 and are eligible for analysis of the primary endpoint. In the end, this gives a total of 50 participants who will complete the study according to full analysis set for analysis of the primary endpoint.

Only the study nurse, investigator and the PI will be able to connect each study subject with their enrolment number or randomization number. Identification of individual study subjects will only be performed under special circumstances, for example, if a study subject requests removal of his or her study data.

Study subject eligibility will be established before enrolment and randomization; to be included in the study, the study subject must meet all inclusion and no exclusion criteria. Study subjects will be randomized as study subjects are eligible for randomization. If a study subject discontinues from the study, the study enrolment number or randomization number will not be reused, and the study subject will not be allowed to re-enter the study.

5.5. Withdrawal criteria

Subjects can discontinue their participation in the study at any time without any consequence to further treatment. The subjects may be withdrawn from the study at the discretion of the investigator due to safety concerns or if judged non-compliant with study procedures. If the subject discontinues the study, follow-up of this subject will be performed according to the clinic’s routine. In either case, serious adverse events will be followed up. Other reasons for discontinuing a subject are incorrect enrolment and subjects lost to follow-up.

All inclusion criteria must be fulfilled, and all exclusion criteria must not be fulfilled for all subjects throughout the whole study. If a subject is non-compliant with the protocol but still wishes to continue its participation in the study it is up to the investigator to judge the subjects possible continuation. This situation could for example occur if a subject temporarily is affected by illness

and therefore misses some of the study procedures. In case of a situation like this it is up to the investigator to judge further eligibility of the subject to continue the study.

5.5.1. Premature termination of the study

The sponsor may decide to stop the trial or part of the trial at any time. If a trial is prematurely terminated or suspended, the investigator should promptly inform the participants and ensure appropriate therapy and follow-up. Furthermore, the investigator should promptly inform the Ethics Committee and provide a detailed written explanation. The regulatory authority should be informed according to national regulations.

For subjects that terminate the study in advance economic compensation will be given according to percentage of completion based on the economic compensation received if completing the study. Percentage of completion based on number of study visits.

5.6. Re-screening

Subjects who fail to meet the inclusion and no exclusion criterions on the initial screening visit will be excluded from the study and will not be eligible for re-screening.

6. STUDY TREATMENTS

No drugs or medicinal treatments are used in this study. However, study subjects are obliged to wear a weight vest with 11 % or 1 % of the subject’s body weight. As discussed previously we judge that this is a study with few risks and only mild potential adverse effects.

6.1. Randomization

Subjects are included consecutively as they are found to be eligible for inclusion in the study. After the baseline period at day 0 subjects will be randomized in a 1:1 ratio into either the high load group (heavy weight vest) or the low load group (light weight vest). Randomization will be through permuted blocks, stratified by age 18-50 years, age 51-65 years and gender. It will be performed in the Case Report Form (eCRF) using a built-in function for the purpose. If a subject discontinues their study participation, their randomization code will not be reused and the subject will not be allowed to reenter the study again.

6.2. Concomitant medications

Medications that are considered necessary for the safety and well-being of the subject can be given at the discretion of the investigator, unless otherwise specified as an exclusion criterion. Concomitant medications should be reported in a specific log and the electronic eCRF.

7. METHODS FOR MEASUREMENTS OF ENDPOINTS

7.1. Methods for measurement of endpoints for clinical efficacy

7.1.1. Methods for primary endpoint

7.1.1.1. Body weight

Body weight will be recorded in kilograms and grams through a body weight scales (seca scales 704, seca, Germany) which will be performed by a study nurse. The scales are calibrated once a year to make certain reliable measurements. Body weight measurement shall be performed

approximately at the same time of the day (+/- 2 hours) on study visits day -21, -13, 0, 15, 28, 35 and 49. Subjects shall be weighed in undergarment and without shoes. Measurements will be performed by study personnel at Clinical Trial Center (CTC), Gothia Forum, Sahlgrenska University Hospital.

7.1.2. Methods for secondary endpoint(s)

7.1.2.1. Bioelectrical impedance analysis (BIA)

Fat mass, water mass and fat free mass will be recorded in absolute values (g) and as % of total body mass through bioelectrical impedance analysis (BIA) (MC-180MA, Tanita) which will be performed by a study nurse. Body weight measurement shall be performed approximately at the same time of the day (+/- 2 hours) on study visits day -21, 0, 15, 28, 35 and 49. Subjects shall be analyzed in undergarment and without shoes. Measurements will be performed by study personnel at Clinical Trial Center (CTC), Gothia Forum, Sahlgrenska University Hospital.

***7.1.2.2.** Dual-energy X-ray absorptiometry (DXA)*

Dual-energy X-ray absorptiometry (DXA) scans are to be performed at study day 0 (+/- 3 days) and day 35 (+/- 2 days). A DXA-scan of the model Lunar iDXA, encore version 16, SP 1, will be used and the procedure shall be carried out according to local routines to obtain a body composition representation which can differentiate between body fat and fat free mass. Measurements will provide absolute levels of in fat mass (g) and fat free mass (g). The total time for one scan is expected to take 15 minutes. Images will be stored locally on a computer connected to the DXA-scan and will not be accessible to unauthorized personnel. Images and data will be exported for further data analysis. DXA-scan will be executed at Center for Health and Performance at University of Gothenburg. Stefan Pettersson, PhD, Center for Health and Performance, University of Gothenburg, is responsible for the execution and interpretation of these measurements.

***7.1.2.3.** Computed Tomography (CT) scan*

A CT scan of the abdomen and the liver will be performed at day 0 (+/- 3 days) and day 35 (+/- 3 days). The CT scans will be performed at the department of radiology at the Sahlgrenska University Hospital, using a computer tomograph of the model Siemens Somatom Force. Research nurses Marit Johannesson and Niklas Lundqvist have contributed to the development of the protocol and are responsible for the execution of these examinations.

Each CT scan will provide images, from each image the area (cm^2) of visceral fat, subcutaneous fat and total abdominal fat (subcutaneous plus visceral fat) will be measured. An average of the areas from these images will be calculated for each fat depot. Change in average area of visceral fat, subcutaneous fat and total abdominal fat will then be analyzed. Liver fat will be measured in Hounsfield units (HU) on CT images. CT image analysis from topographic images may also be performed to investigate any differences between day 35 and baseline, for example with respect to change in volume or intensity. Changes will be analyzed in both absolute values and relative values (% of baseline value) from CT images of the abdomen.

The images will be stored in the radiology department imaging storage database named *Bild och Funktionsregistret* (BFR) at Sahlgrenska University Hospital. After the CT scans an experienced radiologist at Sahlgrenska University Hospital will examine all scans for accidental medical findings. The investigator will be informed of any accidental medical findings of relevance as judged by the radiologist. The investigator is then responsible to determine and handle further need of investigation of the finding.

Images from both CT scans will then be pseudonymized and transferred to the Uppsala University to be analyzed by researchers with expertise in automated image analysis. Images will be sent on a portable secure hard drive. Associate Professor Joel Kullberg, Uppsala University, is responsible for the interpretation of these measurements.

7.1.2.4. 24-hour ECG recording

Two 24-hour ECG recordings will be performed, first at day -13 and a second at day 15. All electrodes and other equipment needed to perform the measurement will be attached by a study nurse at the study center. Electrodes will be placed on the chest according to local standards. After the electrodes have been positioned and the recording started, the participant will rest in a supine position for one hour. This will provide a standardized recording at basal conditions with low risk for external noise for evaluation of the autonomic nervous activity on the heart assessed from the heart rate variability (HRV). HRV will be assessed both during the one-hour and the entire 24-hour periods. Time and frequency domain measures will be calculated using commercial software. The primary outcome measure will be the high- and low frequency components and their ratio in the power spectral analysis both as absolute values and normalized to the total power minus the very low frequency component.

The ECG recording will be used to calculate autonomic nervous system activity or tone between day -13 and day 15. HRV measures, together with blood pressure measurements on day -13 and 15, can be used as markers for parasympathetic and sympathetic nervous activities and their relation. The analysis will be performed by a Biomedical analysist at the department of Cardiology, Sahlgrenska University Hospital, using recorders and software for HRV analysis (Pathfinder SL, Spacelabs Healthcare) purchased from Infiniti Medical AB, Täby, Sweden. Responsible for interpretation of the HRV analysis and check for possible abnormalities in the ECG recording is senior professor Lennart Bergfeldt at Sahlgrenska University Hospital.

***7.1.2.5.** Height*

Height will be measured in centimeters with a stadiometer without shoes, according to local routines at the study center. This will be done at the screening visit at day -21. Height measurements will be used to calculate the participants Body Mass Index (BMI, kg/m^2). Measurement will be performed by study personnel at Clinical Trial Center (CTC), Gothia Forum, Sahlgrenska University Hospital.

***7.1.2.6.** Waist circumference*

Waist circumference (centimeters) will be measured with a measuring tape on study day 0, 15, 28, 35 and 49. Waist circumference should be measured with the subject in a standing position at the midpoint between the lower margin of the least palpable rib and the top of the iliac crest, using a stretch-resistant tape. The subject shall stand with feet close together, arms at the side and body weight evenly distributed, and should wear little clothing. The subject should be relaxed, and the measurements should be taken at the end of a normal expiration. Each measurement should be repeated twice; if the measurements are within 1 cm of one another, the average should be calculated. If the difference between the two measurements exceeds 1 cm, the two measurements should be repeated. Outcome measurements will be waist circumference (cm). Change in waist circumference (in % of baseline (day 0) waist circumference) will be compared between the high load group and the low load group for the different time points (day 15, 28, 35, 49). Measurements will be performed by study personnel at Clinical Trial Center (CTC), Gothia Forum, Sahlgrenska University Hospital.

7.1.2.7. Blood samples

- Procedure
  - All blood samples will be taken by an experienced and trained study nurse at Clinical Trial Center (CTC), Gothia Forum, Sahlgrenska University Hospital according to local protocols.
  - Capillary blood samples:
    - Finger puncture, and a glucometer (Hemocue® glucose system) will be used to instantly be able to measure plasma levels of glucose
  - Venous blood samples:
    - Will be used for other measurements than plasma glucose levels.
    - Each venous blood drawing consists of approximately of 35 ml. Every sample will then be divided in 4-10 tubes. The tubes used for blood drawings will contain ethylenediaminetetraacetic acid (EDTA) or a SST-gel for the purpose of manipulating coagulation.
  - Fasting blood samples (day 0, 15, 35 and 49)
    - Fasting blood samples are required to be obtained after an overnight fast (i.e. fasting for approximately 8 hours). No consumption of solid food is allowed during this time and consumption of liquid is constricted to a maximum of a couple of mouths of water. Participants can rinse their mouth with water if not swallowed. Intense physical activity is prohibited during the fasting period.
  - Samples to be analyzed in a lab at Sahlgrenska academy will be centrifuged to obtain serum or plasma samples at the study center. The samples will then be sent directly to the laboratory for analysis or be stored at - 80 degrees according to biobank agreement with Biobank Väst (registration nr: 890) and according to local laws and regulations.
  - Samples to be analyzed at local lab at Sahlgrenska University Hospital will be sent for analysis immediately after sampling.
- Screening blood samples on study day -21
  - Analysis of samples by local lab at Sahlgrenska University Hospital:
    - Concentration of Hemoglobin (Hb), White Blood Cell Count (WBC), thrombocyte count, sodium (Na), potassium (K), chloride (Cl), calcium (Ca), creatinine, aspartate transaminase (AST), alanine transaminase (ALT), alkaline phosphatase (ALP), total bilirubin, Prothrombin Time (PT-INR), Activated Partial Thromboplastin Time (APTT), human immunodeficiency viruses (HIV), Hepatitis B, Hepatitis C, glycated hemoglobin (HbA1c), C-reactive protein (CRP), free thyroxine (fT4), thyroid stimulating hormone (TSH).
- Blood samples on study day 0, 15, 35 and 49.
  - Analysis by the Sahlgrenska Academy, University of Gothenburg:
    - Concentration of Fibroblast Growth Factor 21 (FGF21), FGF23, FGF15, osteocalcin, sclerostin, lipocalin2, leptin, ghrelin, GLP-1, GLP-2, CCK, (3-36) PYY, glucagon, testosterone, estrogens, renin, glycerol, free fatty acids (FFA) and methoxynorepinephrine.
    - Exploratory analysis of circulating proteins, metabolites or electrolytes. Analysis in form of proteomics or metabolomics may also be performed.
  - Analysis by the local lab at Sahlgrenska University Hospital:
    - Concentration of sodium (Na), potassium (K), chloride (Cl), calcium (Ca), creatinine, C-reactive protein (CRP), insulin, High-density lipoprotein (HDL), Low-density lipoprotein (LDL) and triglycerides.

7.1.2.8. Physical activity

Measurement of physical activity will be done on two occasions: at baseline (day -13 to -7) and the second week of the intervention (day 15 to 21), using tri-axial accelerometers (Axivity AX3, Axivity Ltd, UK). Subjects will wear accelerometers 24 hours a day for seven consecutive days.

The accelerometer will be attached on the lateral aspect of the right iliac crest. The accelerometers are to be attached using a specially designed belt with adjustable length containing a small pocket for the accelerometer. The belt will be attached around the waist to ensure a correct position during the measurement period. Study personnel will show and instruct participants how to attach the accelerometer at the physical visits at the study center on day -13 and on day 15. The accelerometer and the belt are water resistant and can be exposed to water in for example a shower or in a swimming pool. The accelerometer does not handle extreme temperatures. The subjects are recommended to temporarily remove the accelerometer when possibly exposing it to high temperatures, for example in a sauna.

Participants will also be provided with a diary to keep a record of their leisure time, work hours, time in bed, and any periods of non-wear time during the measurement period. Participants will be asked to only remove the accelerometers in case of adverse skin reactions, discomfort or pain, or affected sleep and if going to a sauna. Participants will be asked to return the accelerometers at the study centre at the following physical visit or by using a pre-paid envelope.

The accelerometers are to be initialized, and raw data to be downloaded by study staff at Center for Health and Performance at University of Gothenburg using the manufacturer's software (OMGUI Configuration and Analysis Tool, Axivity Ltd, UK). These measurements will be used to calculate possible differences in physical activity. Outcome measurements will be time distribution in a spectrum of physical activity intensities [15]. Change in time distribution will then be compared in absolute and in relative values (in % of baseline physical activity) between measurements during intervention (day 15 to 21) and baseline (day -13 to -7). Change in the high load group will be compared with the low load group. Unauthorized personnel will not have access to the data from the accelerometers. Professor Mats Börjesson, University of Gothenburg and Sahlgrenska University Hospital, is responsible for the interpretation of these measurements.

7.1.2.9. Energy Expenditure

Energy Expenditure will be measured by using the method doubly labeled water (DLW) and will be performed at baseline (day -14 to 0) and during the second and third week of the intervention (day 14 to 28). Subjects will drink a calculated dose of DLW containing the stable isotopes deuterium (^{2}H) and oxygen-18 (^{18}O). Levels of these isotopes will be measured in urine samples collected for 2 weeks and will give a measure of carbon dioxide production from which energy expenditure can be calculated.

Subjects will receive the material needed for collection of the baseline urine sample (sample 1) and the calculated dose of DLW at the previous visit. They will also get detailed oral and written information about the protocol. On the first day of the DLW measurement the subjects will receive an evening phone call from study personnel repeating the protocol and answering questions. After this telephone visit subjects will collect a baseline urine sample (sample 1) and the subject will drink the dose of doubly labeled water. On the day after drinking the DLW the subject will visit the study center to provide the second urinary sample (sample 2) and for measurement of body weight. The subjects will receive all further materials and instructions needed, they will then by themselves collect urinary sample 3-6 at home according to a supplied protocol. On the last day of

measurement, the subjects will return to the study center to provide the last urinary sample (sample 7) at the study center.

A total of seven urine samples per measurement period will be collected for two weeks. All samples will be collected in seven provided dry plastic containers with airtight locks with a volume of 500-1000 ml. The containers will be marked with subject-ID, sample number, date and time. These containers must not be rinsed or mixed with any other fluids than the urine sample itself. Subjects should urinate directly into the designated containers. They shall be completely dry upon voiding and the airtight lock shall be closed directly after collecting the urine sample. Any spilling should be removed with a dry tissue. All samples collected at home are to be stored in a home freezer and shall be returned to the study center at the following physical visit or at another appointed time. Upon transportation of the urine samples to the study center it is tolerable for the samples to thaw but it is important that the containers are sealed properly to avoid loss of fluid or mixture with surrounding air.

At the study center 4 ml of urine will be transferred from each sample into two separate glass vials (2 ml urine in each vial). These vials will be stored in a -20°C freezer according to the biobank agreement with Biobank Väst. One of these vials will then be sent for analysis in a laboratory at the University of Maastricht, the Netherlands. Outcome measurements will be absolute (Joule per day) and relative (% change compared to baseline) change in energy expenditure expressed. Change in energy expenditure will be compared between measurement during intervention (day 14 to 28) and baseline (day -14 to 0). Change in the high load group will be compared with the low load group. Sending and storage of all samples will follow all necessary laws and regulations. Responsible for this protocol and interpretation of the results is Professor Klaas Westerterp, Maastricht University.

7.1.2.10. Food intake

To estimate food intake a validated food questionnaire called *Short Dietary Questionnarie* (SDQ) will be used. Participants will fill out the questionnaire at two time points, first on day 0 and secondly on day 28. This will be done digitally at the study center either using their own mobile phone or using a designated computer. At each time point the questionnaire will estimate the subject’s food intake during the previous 2 weeks. Outcome measurements will be absolute (calories) and relative (% compared with baseline) change in food intake. Changes compared between measurement during intervention (day 14 to 28) and baseline (day -14 to 0). Change in the high load group compared with the low load group. The full questionnaire can be found in attachments *17.1 Short Dietary Questionnarie*. Responsible for the protocol of the diet registration and its interpretation is Professor Christel Larsson, University of Gothenburg.

7.1.2.11. Weight vest diary

All subjects will at the start of intervention on day 0 be given a diary for daily registration of number of hours wearing the weight vest. In the diary the subject is to register time wearing the weight vest and number of hours standing during the day with the weight vest. This diary is to be recorded for each day during intervention (day 0 to day 35) and thereafter to be returned to the study personnel at the study center. Data from this diary will calculate deviation from the mandatory vest wearing time of 8 hours per day as a mean of the total vest wearing time for all 5 intervention weeks, this at the end of the intervention (day 35). It will likewise calculate a subject’s time in a standing position. Participants will be encouraged to use the vest standing > 2 hours per day. The full diary can be found in attachments 17.4.

7.2. Methods for measurement of endpoints for clinical safety

7.2.1.1. Physical examination

A physical examination will be performed at the study center on day -21, 0, 15, 35 and 49. The examination shall be performed by an investigator or the PI who also shall be a medical doctor. The examination is performed as a health screening to make sure participants are eligible to the study according to the inclusion and exclusion criteria. Abnormal findings shall be judged by PI/investigator if clinically significant. Clinically significant abnormal findings discovered at screening shall be documented as medical history and if found after screening these shall be documented as an adverse event.
The physical examination shall include general appearance, auscultation of heart and lungs, abdominal examination, neurological examination and examination of the oral cavity.

7.2.1.2. Blood pressure

Blood pressure will be taken at the study center on day -21, 0, 15, 35 and 49 by an experienced study nurse. It shall be measured as the first parameter during each visit. It should be measured in the left or right arm, in lying position after the subject have been lying down for between 5 to 10 minutes. Blood pressure shall be measured in the identical arm (left or right) at every measurement. Blood pressure is measured using the same kind of calibrated standard equipment for every measurement (Dinamap blood pressure machine). The examination is performed as a health screening to make sure participants are eligible to the study according to the inclusion and exclusion criteria.

7.2.1.3. Electrocardiography (ECG) recording

A 12-lead ECG recording will be performed at screening on day -21 at the study center. It should be measured while the subject is in a supine position. Electrodes are to be placed across the chest according to local routines. ECG is measured using the same kind of calibrated standard equipment for every subject. Each recording should be interpreted by an investigator to scan for eventual abnormalities. The examination is performed as a health screening to make sure participants are eligible to the study according to the inclusion and exclusion criteria.

7.2.1.4. Pregnancy

A pregnancy test will be performed at screening according to the inclusion criteria. If, throughout the study, there is any uncertainty regarding if a participant might be pregnant as judged by study personnel a pregnancy test must be taken. This to evaluate eligibility in the study according to inclusion and exclusion criteria. The pregnancy test will be taken using a high sensitivity urine pregnancy test strip to measure levels of human chorionic gonadotropin (hCG) in a urine sample. The urine sample will be collected and analyzed for urine levels of hCG at the study center. This to determine any eventual pregnancy.
Pregnancy at any time point during the study will lead to withdrawal from the study as soon as the information reaches the investigator, this even if the pregnancy is only temporary due to for example abortion or miscarriage. The subject and the pregnancy should be followed according to procedures described in the safety section in this document.

7.2.1.5. Illness or symptoms related to infectious diseases

All participants will be instructed only to attend physical visits at the study center if feeling healthy and if they are without any symptoms related to any infectious diseases. In case of illness or symptom/symptoms related to infectious diseases such as fever with a body temperature of > 37,5° C, shivering, sore throat, cough, headache or general muscle pain participants are instructed

to stay at home. Participants will be reminded to follow these rules upon all study visits. Body temperature is checked on every physical visit at Clinical Trial Center, Gothia Forum.

With regards to the current covid-19 pandemic study participants and study personnel are obliged to follow current national and local guidelines in Sweden and Västra Götalandsregionen, this also regarding to when it is appropriate to visit the study center. For example, these recommendations include to follow the recommended time-periods for how long a participant need to have been without symptoms before visiting the study center, how to manage if someone near the participant is ill or have been confirmed having covid-19. It is a risk for both the subject and the study personnel to get infected with covid-19 at physical visits. To minimize the risks of spreading the infection all study personnel are to follow local guidelines to use appropriate protection clothing which, if recommended, include a visor, gloves and a face mask.

If appropriate, a subject with symptoms of covid-19 may be offered an acute Polymerase Chain Reaction (PCR) test for covid-19 to conclude if the symptoms are caused by covid-19. Testing will then be according to local routines at Sahlgrenska University Hospital. This test may be offered if a negative result makes it possible to attend a physical visit at the study center within the visiting window. A positive PCR test for covid-19 will lead to exclusion of the subject from the study. Applicability of testing will be judged by the investigator. If no acute test is applicable, subjects will be encouraged to visit its own local primary care center for testing according to local guidelines.

8. SAFETY

8.1. Definitions Adverse Events

8.1.1. Adverse events (AE)

Adverse Event (AE): Any untoward medical occurrence in a clinical investigation subject administered a medicinal product and, which does not necessarily have a causal relationship with the treatment, can be an unfavorable and unintended sign (including an abnormal laboratory discovery), symptom or disease temporally associated with the use of the medicinal (investigational) product, in this trial being the weight vest. This whether or not the medical occurrence is related to the medicinal (investigational) product.

If an adverse event occurs the study subject can abort the study whenever he or she wants to. The medical personnel involved in the study will help the subject to the correct medical care giver practice if needs arise. Adverse events will be reported in the publication.

8.1.2. Serious Adverse events (SAE)

Serious adverse event (SAE): Any untoward medical occurrence that at any dose:

- results in death
- is life-threatening
- requires inpatient hospitalization or prolongation of existing hospitalization
- results in persistent or significant disability or incapacity
- results in a congenital anomaly/malformation

8.2. Assessment of Adverse Events

8.2.1. Assessment of casual relationship

The PI is responsible for determining whether there is a causal relationship between the AE/SAE and use of the investigational product, i.e., the weight vest.

Those AEs which are suspected of having a relationship to the investigational product will be followed up until the subject has recovered, is well taken care of and on their way to good recovery or until the state of the AE have become persistent (see also section 8.4, Follow-up of Adverse Events).

All AE will be categorized either as likely related, possibly related, or not related, in accordance with the definitions below:

Likely related: Clinical event, including abnormal results from laboratory analyses, occurring within a reasonable time after administration of the intervention/investigational product. It is unlikely that the event can be attributed to underlying disease or other medications but is most likely caused by the investigational product and its emergence is reasonable in relationship with use of the investigational product.

Possibly related: Clinical event, including abnormal results from laboratory analyses, occurring within a reasonable time after administration of the intervention/investigational product. The event could be explained by the investigational product and its emergence is reasonable in relationship with use of the investigational product, but there is insufficient information to determine the relationship. The event could be explained by an underlying disease or other medications.

Not related: Clinical event, including abnormal results from laboratory analyses, that is not reasonably related to the use of the intervention/investigational product. The event is unlikely related to the intervention/investigational product and can be explained by other medications or underlying disease.

8.2.2. Assessment of intensity

Each adverse event shall be classified by an investigator as mild, moderate or severe.

Mild: The adverse event is relatively tolerable and transient in nature but does not affect the subject’s normal life.

Moderate: The adverse event causes deterioration of function but does not affect health. The event can be sufficiently unpleasant and interferes with normal activities but does not completely obstruct them.

Severe: The adverse event causes deterioration of function or work ability or poses a health risk to the subject.

8.2.3. Assessment of seriousness

The investigator is responsible for assessing the seriousness (serious or non-serious). If the incident is considered serious, this should be reported as a serious adverse event (SAE) by the investigator to the PI. See also section 8.3.2, Reporting of Serious Adverse Events (SAE).

8.3. Reporting and registration of Adverse Events

At each study visit, adverse events (AE) are registered, starting after a signed informed consent form (ICF) up to and including 2 weeks after the subject has ended their treatment with the weight vest. All AE that occurs during the study and which are observed by the investigator/study nurse or reported by the subject will be registered in an AE log and in the CRF regardless of whether they are related to the weight vest or not. Assessment of causal relationship, severity, and whether the AE is an SAE or not will be done by the investigator in an AE log and in the CRF. At minimum, for each AE/SAE, a description of the event is recorded (diagnosis/symptom if diagnosis is missing), start and stop dates, causal relationship, severity, if the AE is an SAE or not, measures and outcome. Follow-up information should be provided as necessary.

It will be left to the Investigator's clinical judgment to decide whether an AE is of sufficient severity to require the participant's removal from treatment. A subject may also voluntarily withdraw from treatment due to what he or she perceives as an intolerable AE. If either of these occurs, the participant must undergo an end of trial assessment and be given appropriate care under medical supervision until symptoms cease, or the condition becomes stable.

8.3.1. Reporting of Adverse Events (AE)

All AE shall be registered in the CRF within 2 workdays as above (section 8.3, Reporting and registration of Adverse Events).

8.3.2. Reporting of Serious Adverse Events (SAE)

Serious adverse events (SAE) are reported to the sponsor on a special SAE form which will be sent via mail within 24 hours of the investigator being informed of the SAE.
Follow-up information describing the outcome and handling of the SAE is reported within 24 hours after this information is available. The original should be kept in the Investigator Site File (ISF). All SAE will be registered in an AE log and in the CRF.

8.4. Follow-up of Adverse Events

Adverse events will be monitored from screening to the last visit of the study. Follow-up will be done by study personnel at every study specific visit. Follow-up of the adverse event during the study will be done until the event is resolved, stable or have become persistent. Unacceptable adverse events, as judged by the investigator, can lead to withdrawal of the participant from the study. Any still ongoing adverse events at the end of the study will be reviewed by the investigator to determine appropriate follow-up.

8.5. Procedures in case of emergencies or pregnancy

The PI and investigator are obliged to immediately take the urgent safety measures necessary to protect the subjects from immediate danger. Examples of such measures are to temporarily suspend the clinical trial or to introduce supplementary monitoring measures. The PI shall inform Swedish Ethical Review Authority (Etikprövningsmyndigheten (EPM)) as soon as possible about the urgent safety measures taken by the investigator or PI.

Pregnancy is reported to the sponsor on a special pregnancy form which will be sent via mail within 24 hours of the investigator being informed of the pregnancy. Follow-up information describing the outcome (continuation of pregnancy, abortion or miscarriage) and handling of the pregnancy is reported within 24 hours after this information is available. The original should be kept in the Investigator Site File (ISF). Pregnancy will lead to withdrawal from the study due to safety reasons. Pregnancy will be registered in the CRF.

The carrying of a weight vest is judged to be associated with low and few risks for the subjects. As judged by the research group the risk for congenital malformation on an eventual fetus/child if a subject is to be pregnant is very low. Due to this low risk of congenital complications an eventual pregnancy will be followed until the end of the study and necessarily not until birth has taken place. Any still ongoing pregnancy at the end of the study will be reviewed by the investigator to determine need of any further follow-up.

9. STATISTICS

9.1. Analysis population

The aim of this trial is to further elucidate the effect increased artificial loading has on body weight. It is important to identify if there is any effect of increased loading on obese subjects under optimal conditions and if all subjects are fully compliant to the study protocol. Therefore all participants that complete the study with regards to the primary outcome will be included in the analysis, e.g. analyses applicable to Full Analysis Set (FAS). A secondary per protocol analysis may be performed with regards to the study endpoints.

9.2. Statistical analyses

9.2.1. Statistical methods

The primary endpoint of this randomized study will be the change in body weight at the end of the intervention (day 35) expressed as percent of original body weight at day 0, i.e. the day of the start of the intervention. The values will be calculated for all individuals participating in the study. The values will then be compared between the groups of individuals exposed of 11% and 1 % of artificial loading, using ANCOVA (assuming normality).

- An analysis of covariance (ANCOVA) using age, BMI at the start of the study, sex, time per day wearing the west (hours/day), and percent of the vest bearing time standing up as co-variates will be performed.
- Non-parametric analysis will be performed if data cannot be assumed normally distributed even after transformation.

The secondary endpoints of this study are found under section 3.2 in the study protocol. All the secondary endpoints are regarded as exploratory endpoints. A selection of the secondary endpoints includes change in:

- Body weight at day 15 and day 28 expressed as absolute (g) and relative (% of baseline value) change compared to baseline.
- Abdominal, visceral and subcutaneous fat at day 35 (end of intervention) expressed as absolute (g) and relative (% of baseline value) change compared to baseline.
- Fat free mass and fat mass at day 15, day 28 and day 35 expressed as absolute (g) and relative (% of baseline value) change compared to baseline.
- Energy expenditure during intervention (day 14-28) expressed as absolute (Joule per day) and relative (% of baseline value) change compared to baseline (day -14 to day 0).
- Physical activity during intervention (day 15-21) expressed as absolute (time distribution in a spectrum of physical activity intensities) and relative (% of baseline value) change compared to baseline (day -13 to -7).
- Waist circumference at day 15, day 28 and day 35 expressed as absolute (cm) and relative (% of baseline value) change compared to baseline (day 0).
- Heart rate variability (HRV) during intervention (day 15) expressed as absolute (frequency) and relative (% of baseline value) change compared to baseline.

- Serum concentration at day 15 and day 35 of circulating proteins, metabolites or electrolytes compared to baseline.
- Analysis at day 15 and day 35 of proteomics or metabolomics compared to baseline.

These will be analyzed using the same methods as described for the primary endpoint. We will also compare absolute changes between the groups of individuals exposed to 11% and 1 % artificial loading.

From the ANCOVA models adjusted for covariates, least square means (LSM) with 95 % confidence intervals will be calculated. P-values < 0.05 will be classified as significant. The p-values for within group comparison (5 weeks vs baseline) of the different parameters will be calculated using Wilcoxon signed rank-sum test.

9.2.2. Drop-outs

Subjects who have not completed study visit on day 0 (start of intervention) and on day 35 (end of intervention) will be excluded from the analysis due to not being eligible for analysis of the primary endpoint.

Subjects who have used the weight vest less than 80% of requested time will be excluded from a possible additional per protocol analysis but not from the primary FAS analysis. Wearing time will be calculated as a mean of the total wearing time for the 5 weeks of intervention. Wearing time of 8 hours per day gives a total wearing time of 280 hours and subjects are thus required to wear the vest for at least 224 hours during the intervention period. This will be calculated after the end of intervention (day 35) based on self-reporting

Subjects who have been in a standing position (while wearing the weight vest) less than 80% of requested time will be excluded from a possible additional per protocol analysis but not from the primary FAS analysis. Standing time will be calculated as a mean of the total standing time for the 5 weeks of intervention. Standing time of 2 hours per day gives a total wearing time of 70 hours and subjects are thus required to be in a standing position for at least 56 hours during the intervention period. This will be calculated after the end of intervention (day 35) based on self-reporting

9.3. Sample size calculation

It is anticipated that a total of approximately 130 subjects are required to be screened to identify a statically significant ($p<0.05$) effect of 1.6% difference in relative body weight change between 5 weeks and baseline, when comparing high load with the low load group with 80% power. These calculations will allow a screening failure of 50% and a dropout rate of 20% after screening. Calculations are based on a standard deviation of 2% for change in body weight comparing 5 weeks with baseline. Power calculations demonstrate that a total of 25 evaluable participants per group are needed to significantly detect above mentioned effect size with a power of 80%.

Subjects will be screened and included in the clinical trial consequently until 25 subjects in the heavy weight vest group (intervention/high load group) and 25 subjects in the light weight vest group (control/low load group) are certain to complete the trial and be eligible for Full Analysis Set (FAS). Certainty will occur when that number of subjects have finished the intervention on day 35. Eligibility to Full Analysis Set (FAS) is defined as having completed the study visits on randomization (day 0) and at the end of intervention (day 35) and thus being eligible for assessment of the primary outcome. Screening and inclusion will probably be executed according to above mentioned calculations but could be adjusted along the timeline of the trial.

10. QUALITY CONTROL AND QUALITY ASSURANCE

10.1. Quality Assurance and Oversight

The PI is responsible for assuring that all study personnel involved in the are properly trained before getting involved in any study specific procedures or meet any study participants. Training of study personnel will be carried out through reading of the latest version of the study protocol, meetings or other documents provided by the PI. The training can be received directly from the PI or other trained study personnel after delegation from the PI. Working manuals for study specific procedures will be provided to minimize errors or inconsistency at study visits.

The PI shall allow study-related monitoring, auditing, and regulatory inspections by providing direct access to the CRF, paper print out from medical record, as well as other source data and other study-specific documentation. This shall also be apparent to the subjects in the Subject Information and Informed Consent form.

The sponsor shall construct a study monitoring plan which will be based on the identified risks, as well as follow-up of risks during the study.

10.2. Monitoring

The study will be monitored by an independent monitor before the study begins, during the study conduct, and after the study has been completed, so as to ensure that the study is carried out according to the protocol and that data is collected, documented, and reported according to ICH-GCP and applicable ethical and regulatory requirements. Monitoring is performed as per the study's monitoring plan and is intended to ensure that the subject's rights, safety, and well-being are met as well as data in the CRF are complete, correct, and consistent with the source data.

A study monitor will be appointed by the sponsor. The monitor will be appropriately trained and informed about the nature of the study, subject written information, GCP and applicable regulatory requirements.

The monitor will have regular contacts with the clinic to verify informed consents of participating subjects, to confirm that facilities remain acceptable, that the investigational team is adhering to the protocol, that data are being accurately recorded in the CRFs, to verify inclusion/exclusion criteria, study main endpoints, check AE/SAE reporting and that therapy accountability is being carried out. The investigator should ensure that all persons assisting with the trial are adequately informed and trained about the protocol, the investigational products(s) and their trial related duties and factions. The monitor will check that training has been performed and that this is documented. The monitor will also ensure source data verification (comparison of the data in the CRF with the medical records and other source data). The monitor must have direct access to source data. The extent of monitoring will be defined in a monitoring plan.

10.3. Source Data

The investigator must keep source documents for each subject in the study. A document describing what has been classified as source data in the study, a source data log, should be included in the Investigator Site File (ISF). The investigator must ensure that all source documents are accessible for monitoring and other quality control activities. The CRF is defined as source data in this study.

10.4. Deviations or serious breaches

Serious breaches and deviations from the study protocol, GCP and other regulations that significantly and directly affects, or with high likelihood could affect, the subjects or the scientific value of the study, shall be immediately reported within 7 days (from knowledge) to the PI. It is the PI's responsibility to judge the consequences of deviations that have occurred, and thus also to decide whether any Swedish Authorities should be informed.

Minor deviations that do not affect subjects' integrity or safety, nor significantly affect the study's scientific value, are documented in the study documentation of the principal investigator and the PI.

10.5. Audits and inspections

Authorized representatives of the PI, a Competent Authority or an Ethics Committee may perform audits or inspection at the center, including source data verification. The investigator must ensure that all source documents are accessible for auditing and inspection. The purpose of an audit or inspection is to systematically and independently examine all study-related activities and documents, to determine whether these activities were conducted, and data were recorded, analyzed and accurately reported according to the protocol, Good Clinical Practice (GCP) and any applicable regulatory requirements.

11. ETHICS

11.1. Compliance to the protocol, GCP and regulations

The study will be performed in compliance with the study protocol, the Declaration of Helsinki, ICH-GCP (Good Clinical Practice) guidelines and current national and international regulations governing this clinical trial. This is to ensure the safety and integrity of the study subjects as well as the quality of the data collected.

11.2. Ethical review of the study

The final study protocol for clinical trials must be approved, as a part of the application for a permit for clinical trials, by the Swedish Ethical Review Authority (Etikprövningsmyndigheten, EPM) before the trial can be conducted. The final version of the informed consent form and other information provided to subjects, must be approved or given a written positive opinion by EPM. EPM must be informed of any changes in the study protocol in accordance with current requirements.

11.3. Procedure for obtaining informed consent

The principal investigator shall ensure that the subject is given full and adequate oral and written information about the study, its purpose, any risks and benefits as well as inclusion and exclusion criteria. Subjects must also be informed that they are free to discontinue their participation in the study at any time without having to provide a reason. Subjects should be given the opportunity to ask questions and be allowed time to consider the provided information.

If the person chooses to participate, both the subject and the investigator shall sign the informed consent form. A copy of the subject information as well as the informed consent form shall be provided to the subject. The subject's signed and dated informed consent must be obtained before performing any study-specific activity in the study. Each subject who participated in the study will be identified by a subject number on a subject identification list. The subject agrees that monitors, auditors, and inspectors may have access to their medical records and other source data. If new information is added to the study, the subject has the right to reconsider whether he/she will continue their participation.

11.4. Subject data protection

If any part of the data is handled by any other organization, inside or outside the EU, appropriate agreements and/or other documentation will be established, to ensure that the data processing is performed in accordance with the provisions of the General Data Protection Regulation (GDPR) and other relevant legislation, before any data transfer takes place.

The content of the informed consent form complies with relevant integrity and data protection legislation. In the subject information and the informed consent form, the subject will be given complete information about how collection, use and publication of their study data will take place. The subject information and the informed consent form will explain how study data are stored to maintain confidentiality in accordance with national data legislation in a computer database. All information processed by the PI will be pseudonymized and identified with a study code.

The informed consent form will also explain that for verification of the data, authorized representatives of the PI, as well as relevant authority, may require access to parts of medical records or study records that are relevant to the study, including the subject's medical history.

11.5. Insurances

The study subjects are covered by the Swedish Patient Injury Act.

12. PROTOCOL DEVIATIONS AND AMENDMENTS

Substantial changes to the signed study protocol are only possible through approved protocol amendments and by agreement from all responsible persons. Information on non-substantial changes should be clearly noted in the amended protocol.

In the event of substantial changes to the protocol (e.g., changing of the main objective, primary or secondary variables, method to measure the primary variable, changing of the investigational product or dosage) will be made during the course of the study, approval from the Swedish Ethical Review Authority (Etikprövningsmyndigheten, EPM) shall be obtained before any changes are implemented. A change that concerns a new site, new investigator or a new study patient information sheet shall also be approved by EPM.

Non-substantial changes will be recorded and later entered in documentation that is submitted, for example in any subsequent notifications of a substantial change or in connection with End of Trial reporting.

The Investigator must not implement any deviation from, or change to the protocol, without discussion with, and agreement by the PI and prior review and documented approval/favorable opinion of the amendment from the relevant ethics committee and competent authority, except where it is necessary to eliminate an immediate hazard to study subjects, or where the change(s) involves only logistical or administrative aspects of the study (e.g. change in monitor(s), change of telephone numbers).

13. DATA MANAGEMENT

13.1. Recording of data

Subjects who participate in the study are coded with a specific study identification number. All subjects are registered in a subject identification list (subject enrolment and identification list) that connects the subject's name and personal number with a study identification number. The

investigator will ensure that all data collected in the study are recorded in a timely manner according to any instructions provided.

13.1.1. Source data

The investigator must maintain source documents for each subject in the study. A source data verification log will be included in the Investigator Study File (ISF) defining source data documents. The investigator must ensure that all source documents are accessible for monitoring. The electronic CRF (eCRF) is defined as source data for some study data according to the source data log.

13.2. Data storage and management

All data will be registered, managed, and stored in a manner that enables correct reporting, interpretation, and verification. The complete Trial Master File, as well as source documents, will be archived for at least 10 years after the study is completed. Source data in the medical records system is stored and archived in accordance with Sahlgrenska University Hospital regulations.

Study personnel at the study center (Clinical Trial Center, Gothia Forum, Sahlgrenska University Hospital) will review the data entered in the CRFs for completeness and accuracy. Any required corrections or additions will be done by study personnel. Queries are issued electronically. An Investigator is required to respond to the query and confirm or correct the data.

At the conclusion of the study, the occurrence of any protocol deviations will be determined. After these actions have been completed and the database has been declared to be complete and accurate, it will be locked and available for data analysis. Data analysis will be done either at Sahlgrenska University Hospital, Faculty of Education at University of Gothenburg or at Sahlgrenska Academy at University of Gothenburg.

13.3. Case Report Form (Forskningspersonsformulär)

A Case Report Form (CRF) is used for data collection. In this study an electronic Case Report Form (eCRF) will be used. The PI must ensure that data is registered and any corrections in the CRF are made as stated in the study protocol and in accordance with the instructions. The PI must ensure that the registered data is correct, complete, and that reporting takes place according to the timelines that have been predefined and agreed. The PI signs the completed CRF. A copy of the completed CRF will be archived by the study site.

If an examination/test is not performed and data does not exist, ND (Not done) or NK (Not known) is marked. If the question is irrelevant NA (Not applicable) is written. Corrections in the paper CRF are done by striking out the incorrect information and adding the correct

14. NOTIFICATION OF STUDY COMPLETION, REPORTING AND PUBLICATION

Within one year after the study is completed, the results shall be analyzed, a clinical study report with individual data shall be prepared, and the study results shall also be reported in the ClinicalTrials.Gov database. In addition, upon study completion and finalization of the study report the results of this trial will be either submitted for publication and/or posted in a publicly accessible database of clinical trial results.

15. STUDY TIMETABLE

15.1. Study period

The study will take place during 2021 and possibly 2022. All analyzes shall be finished before 2022-12-31. Publications of the results are planned for 2022 or possibly 2023. All dates are preliminary and may change depending on for example the Covid-19 pandemic.

16. REFERENCES

1. Ohlsson, C., et al., *Increased weight loading reduces body weight and body fat in obese subjects - A proof of concept randomized clinical trial.* EClinicalMedicine, 2020. **22**: p. 100338.
2. Larsson, I., et al., *Body composition in the SOS (Swedish Obese Subjects) reference study.* Int J Obes Relat Metab Disord, 2004. **28**(10): p. 1317-24.
3. World Health Organization. *Obesity and overweight*. 2016 2020-04-01; Available from: https://www.who.int/news-room/fact-sheets/detail/obesity-and-overweight.
4. Katzmarzyk, P.T., et al., *Sitting time and mortality from all causes, cardiovascular disease, and cancer.* Med Sci Sports Exerc, 2009. **41**(5): p. 998-1005.
5. Jansson, J.O., et al., *Body weight homeostat that regulates fat mass independently of leptin in rats and mice.* Proc Natl Acad Sci U S A, 2018. **115**(2): p. 427-432.
6. Cannon, W.B., *The Wisdom of the Body*. Vol. Rev. and enl. ed. 1963, New York: WW Norton & Co. 340.
7. Zhang, Y., et al., *Positional cloning of the mouse obese gene and its human homologue.* Nature, 1994. **372**(6505): p. 425-32.
8. Friedman, J., *20 years of leptin: leptin at 20: an overview.* J Endocrinol, 2014. **223**(1): p. T1-8.
9. Frederich, R.C., et al., *Leptin levels reflect body lipid content in mice: evidence for diet-induced resistance to leptin action.* Nat Med, 1995. **1**(12): p. 1311-4.
10. Montague, C.T., et al., *Congenital leptin deficiency is associated with severe early-onset obesity in humans.* Nature, 1997. **387**(6636): p. 903-8.
11. Ohlsson, C., et al., *The Gravitostat Regulates Fat Mass in Obese Male Mice While Leptin Regulates Fat Mass in Lean Male Mice.* Endocrinology, 2018. **159**(7): p. 2676-2682.
12. Larsson, C.L., K.R. Westerterp, and G.K. Johansson, *Validity of reported energy expenditure and energy and protein intakes in Swedish adolescent vegans and omnivores.* Am J Clin Nutr, 2002. **75**(2): p. 268-74.
13. Christensen, S.E., et al., *Two new meal- and web-based interactive food frequency questionnaires: validation of energy and macronutrient intake.* J Med Internet Res, 2013. **15**(6): p. e109.
14. Svensson, A., et al., *Dietary intake assessment in women with different weight and pregnancy status using a short questionnaire.* Public Health Nutr, 2014. **17**(9): p. 1939-48.
15. Fridolfsson, J., et al., *Stronger Association between High Intensity Physical Activity and Cardiometabolic Health with Improved Assessment of the Full Intensity Range Using Accelerometry.* Sensors (Basel), 2020. **20**(4).

17. ATTACHMENTS

17.1. Short Dietary Questionnaire (SDQ)

See separate document (*Bilaga 06 Enkät – Födointag*).

17.2. Physical Activity Diary

See separate document (*Bilaga 06 Dagbok – Fysisk aktivitet*).

17.3. Participant Instructions Doubly Labeled Water

See separate document (*Bilaga 06 Formulär – dubbelmärkt vatten mätning 1 & 2*).

17.4. Weight Vest Diary

See separate document (*Bilaga 06 Dagbok – viktväst*).
