# Supplementary material for: Increased weight-load improves body composition by reducing fat mass and waist circumference, and by increasing lean mass in participants with obesity: a single-centre randomised controlled trial
Source: BMC Med. 2025 May 30;23:317. doi: 10.1186/s12916-025-04143-6 (PMC12123769; doi:10.1186/s12916-025-04143-6)
Supplement: Supplementary file 3 — Additional file 3: Supplementary information. Supplementary methods. Body weight and body composition. Abdominal adipose tissue. Energy expenditure. Physical activity. Blood sampling. Statistical analysis. Supplementary tables. Table S1 – Results: relative changes. Table S2 – Results: absolute changes. Table S3 – Results: relative changes. Table S4 – Results: absolute regional body composition changes. Table S5 – Results: serum/plasma markers. Table S6 – Reported adverse events and serious adverse events. Table S7 – Medical history at baseline for all randomised participants. Table S8 – Concomitant medications. [file 12916_2025_4143_MOESM3_ESM.pdf]

Supplementary information

Increased weight-load improves body composition by reducing fat mass and waist circumference, and by increasing lean mass in participants with obesity: a single-centre randomised controlled trial

Authors
Jakob Bellman, Klaas Westerterp, Loek Wouters, Marit Johannesson, Niklas Lundqvist, Joel Kullberg, Christel Larsson, Mikael Gustafsson, Stefan Pettersson, Jonatan Fridolfsson, Daniel Arvidsson, Mats Börjesson, Dan Curiac, John-Olov Jansson, Per-Anders Jansson, Claes Ohlsson

Contents
Supplementary methods 2
- Body weight and body composition 2
- Abdominal adipose tissue 2
- Energy expenditure 3
- Physical activity 3
- Blood sampling 4
- Statistical analysis 5

Supplementary tables 6
- Table S1 – Results: relative changes (Full Analysis Set [FAS]) 6
- Table S2 – Results: absolute changes (Full Analysis Set [FAS]) 7
- Table S3 – Results: relative changes (Per protocol analysis) 8
- Table S4 – Results: absolute regional body composition changes (Per protocol analysis) 9
- Table S5 – Results: serum/plasma markers (Per protocol analysis) 10
- Table S6 – Reported adverse events and serious adverse events 11
- Table S7 – Medical history at baseline for all randomised participants 13
- Table S8 – Concomitant medications 14

Supplementary methods

Body weight and body composition

Body weight (kilograms) was measured on days -21, -13, 0, 15, 28, 35, and 49 using calibrated scales (Seca 704, Seca, Hamburg, Germany). Body composition was assessed using bioelectrical impedance analysis (BIA; MC-180MA, Tanita) on days -21, 0, 15, 28, 35, and 49. Fat mass (grams), fat-free mass (grams), and lean mass (grams) were additionally evaluated using dual-energy X-ray absorptiometry (DXA) on days 0 and 35.

DXA scans (Lunar iDXA, enCORE version 16, SP1, GE Healthcare, Illinois, USA) were performed in the evening after a three-hour fasting period and after participants had emptied their bladders. The coefficient of variation (CV) for duplicate DXA measurements was 0.69% for body fat, 0.71 kg for fat mass, and 0.76 kg for lean mass. Numerical values from DXA images were obtained using the scanner's built-in software, and results were analysed for the whole body as well as specific regions (arms, legs, trunk [android and gynoid]).

All measurements were conducted by trained and independent study personnel who inspected the data for artifacts or misalignments. Note that BIA data were not reported due to technical malfunctions caused by incorrect scale settings.

Abdominal adipose tissue

Computed tomography (CT) scans (Somatom Force, Siemens Healthcare GmbH, Munich, Germany) were performed on days 0 and 35 to evaluate liver fat (Hounsfield Units [HU]), visceral fat volume (cm^3), and abdominal subcutaneous fat volume (cm^3) following a standardized protocol adapted from the SCAPIS study. Participants were scanned in the evenings in supine position after a three-hour fasting period. Liver images were obtained at the Th11–Th12 level, and abdominal images at the L3–L4 level during an exhaled breath hold.

CT scanning parameters included collimation of 48 x 1.2 mm, a field of view (FOV) of 500 mm, slice thickness of 5 mm, pitch factor 0, 120 kV, reference mAs of 40, and a

scan time of 0.5 seconds. For each section (liver [Th11-Th12] and abdomen [L3-L4]), five slices were acquired, yielding an approximate total thickness of 25 mm per section. CT images were analysed using an in-house developed, semi-automated segmentation software named Deep-Paint.

Waist circumference (cm) was measured on days 0, 15, 28, 35, and 49 using a stretch-resistant measuring tape. Measurements were taken with participants in a standing position, at the midpoint between the lower margin of the least palpable rib and the top of the iliac crest.

Energy expenditure

The Doubly Labelled Water (DLW) method was used to measure energy expenditure (EE; joules per day) during two separate 14-day measurement periods, starting on days -14 and 14. The standard operating procedure from Maastricht University (Maastricht, Netherlands) was followed to measure CO_2 production using doubly labelled water.

In short, a loading dose of water labelled with the stable isotopes ^2H (deuterium) and ^{18}O was administered on days -14 and 14. Doses were individually prepared based on participants' baseline body weight, height, age, and sex. The isotopes are eliminated from the body via different pathways: ^2H is excreted as water, while ^{18}O is eliminated as both water and carbon dioxide. The rate constants for the elimination of the two isotopes were determined using isotope ratio mass spectrometry (IRMS; Delta V Isotope Ratio Mass Spectrometer, Thermo Fisher Scientific, Waltham, MA, USA) from seven urine samples collected during each measurement period. The difference between the elimination rates of the isotopes was used to calculate CO_2 production, which in turn was used to estimate energy expenditure. A food quotient (FQ) of 0.85 was used to estimate the energy equivalent of CO_2 production, assuming a mixed diet typical of a Western population.

Physical activity

Physical activity (PA) was measured over two seven-day periods starting on days -13 and 15. Participants were instructed to wear the tri-axial accelerometers (Axivity AX3, Axivity Ltd., Newcastle upon Tyne, UK) over the hip for seven consecutive days, 24

hours per day, except during water-based activities or high-temperature activities (e.g. swimming or sauna). Raw triaxial data were processed using the 10 Hz frequency extended method (FEM), combined into vector magnitude and reduced into 3-second epochs to provide a measure of PA intensity each epoch (acceleration, milli-g-unit [mg]). Mean daily intensity (mg) was calculated from all epochs and named Mean Daily Activity. Further, cut-points based on metabolic equivalent of tasks (METs) were applied to each epoch to determine time spent in different intensity categories including:

- Sedentary (SED; <1.5 METs)
- Light PA (LPA; 1.5-<3.0 METs)
- Moderate PA (MPA; 3.0-<6.0 METs)
- Vigorous PA (VPA; 6.0-<9.0 METs)
- Very vigorous PA (VVPA; ≥9.0 METs)
- Moderate to vigorous PA (MVPA; sum of MPA, VPA and VVPA)

These MET cut-points were derived from validated thresholds derived in previous studies. Due to minimal time spent in VPA and VVPA, the data were pooled into the MVPA parameter. Wear time was defined as the sum of sedentary, LPA, MPA, VPA and VVPA. Non-wear time was defined as 60 minutes of consecutive zero values with allowance of up to 2 min of output up to the LPA cut-off. Wear time was confirmed using participant diaries, in which participants recorded any periods when the accelerometer was removed as well as their sleeping times. While participants completed wear-time diaries, these were used as a complement to the algorithmic approach rather than as the primary method for determining non-wear periods. A valid day of measurement was defined as at least 10 hours of wear time during wake hours (according to diary), and a valid measurement period was defined as at least five valid days.

Blood sampling

Serum samples were collected on days 0, 15, 35 and 49, immediately frozen and then stored at −80°C until analysis. Leptin levels were measured in fasting serum samples using commercial ELISA kit (R&D Systems; Cat. DLP00, intra-assay variability 1.8%; Minneapolis, MN, USA). Fasting serum glycerol levels were measured with enzymatic

colorimetric method using a CMA 600 Microdialysis analyser (intra-assay variability 7.6%; MDialysis AB, Stockholm, Sweden). Fasting plasma glucose levels were analysed directly using a glucometer (HemoCue Glucose 201 RT; Ängelholm, Sweden; intra-assay variability 2.3%). Remaining blood samples were analysed at Clinical Chemistry Laboratory at the Sahlgrenska University Hospital (Gothenburg, Sweden) using standardised procedures with the Alinity analysis platform (Abbott, Illinois, USA).

Statistical analysis

Power calculations were based on a standard deviation (SD) of 2% for the primary endpoint which was based on the variability in a previous similar study but with three-weeks duration. A screening failure of 50% was anticipated together with a dropout rate of 20% after screening.

The statistical analysis of the primary endpoint was conducted for all randomised subjects with body weight measurements available both at baseline and at five weeks according to full analysis set (58 participants: 30 low load and 28 high load). Aside from one participant who discontinued the study due to adverse events, there were no missing body weight data. For the secondary endpoints, only a small number of data points were missing, and no imputation of missing data for these parameters was performed. Supplementary analyses of primary and secondary endpoints were performed according to per-protocol analysis, excluding those who deviated from the protocol. To be included in the per-protocol analyses, participants should not deviate more than 20% (1.6 h) from the requirement of using the weight vest at least eight hours per day and they were required to follow the protocol criteria specified in the study protocol (Additional file 2).

For comparisons of adverse events between the treatment groups (Table S6), Fisher's exact test was used. Normality of the data was explored using Shapiro–Wilk test, Q-Q-plot, and visual inspection. Statistical analyses for secondary endpoints were not adjusted for multiplicity since they were exploratory.

Supplementary tables

Table S1 – Results: relative changes (Full Analysis Set [FAS])

	Low load (n=30)	High load (n=28)	Difference between groups	p-value (ANCOVA)
**Anthropometrics**				
Body Weight (%)	-0.01 (-0.5, 0.5)	0.04 (-0.5, 0.6)	0.1 (-0.7, 0.8)	0.884
Waist circumference (%)	0.3 (-0.7, 1.2)	**-1.8 (-2.8, -0.9) *** **	-2.1 (-3.5, 0.7)	**0.003**
**DXA scan**				
Fat percent (%)	0.6 (-0.2, 1.3)	**-1.1 (-1.8, -0.3) ** **	-1.6 (-2.7, -0.6)	**0.004**
Fat mass (%)	0.7 (-0.2, 1.6)	**-1.0 (-1.9, -0.03) * **	-1.7 (-3.0, -0.4)	**0.012**
Lean mass (%)	-0.2 (-0.9, 0.5)	**0.9 (0.3, 1.6) * **	1.1 (0.2, 2.1)	**0.022**
BMC (%)	0.04 (-0.3, 0.3)	-0.03 (-0.3, 0.3)	-0.1 (-0.5, 0.4)	0.745
**CT scan**				
VAT (%)	-2.3 (-5.7, 1.1)	-1.3 (-4.7, 2.2)	1.0 (-3.8, 5.9)	0.67
SAT (%)	-0.9 (-2.6, 0.7)	-1.7 (-3.4, -0.01)	-0.8 (-3.2, 1.6)	0.52
Liver fat (%)	0.8 (-1.7, 3.3)	2.2 (-0.4, 4.7)	1.4 (-2.2, 5.0)	0.44
**Physical activity**				
Mean Daily Activity (%)	4.8 (-7.4, 17.0)	-1.9 (-14.5, 10.8)	-6.7 (-24.4, 11.0)	0.451
Sedentary time (%)	**-2.3 (-4.9, 0.3) * **	0.8 (-2.0, 3.4)	3.0 (-0.7, 6.8)	0.112
LPA (%)	**10.3 (0.1, 20.5) * **	7.5 (-3.0, 18.1)	-2.8 (-17.6, 12.0)	0.705
MVPA (%)	4.1 (-9.2, 17.4)	-2.4 (-16.2, 11.4)	-6.5 (-25.8, 2.8)	0.502
**Energy balance**				
Energy expenditure (%)	-1.0 (-3.7, 1.7)	-0.9 (-3.7, 1.8)	0.1 (-3.8, 3.9)	0.968
Energy intake (%)	3.7 (-6.5, 13.9)	4.9 (-5.7, 15.4)	1.2 (-13.6, 15.9)	0.876

Table S1. Analyses of the relative changes in the primary and main secondary endpoints for all participants eligible for full analysis set (FAS). The primary endpoint was the relative change in body weight after 5 weeks of treatment. Results are presented as estimated marginal means with 95% confidence intervals. The between group p-values (High load vs Low load) given within the table are calculated using analysis of covariance (ANCOVA) adjusted for age, sex, baseline BMI, Vest exposure (h) and standing % with vest. Within group comparisons (five weeks vs baseline) were made using Wilcoxon signed-rank test. *p<0.05, **p< 0.01, ***p<0.001. Statistically significant differences are highlighted in **bold**. BMC, Bone Mineral Content; BMI, body mass index; CT, Computed Tomography; DXA, Dual-energy X-ray absorptiometry; HU, Hounsfield unit; Liver fat, estimated as liver attenuation; LPA, light physical activity; MVPA, Moderate to vigorous physical activity; SAT, subcutaneous adipose tissue in the abdominal region; VAT, visceral adipose tissue in the abdominal region.

Table S2 – Results: absolute changes (Full Analysis Set [FAS])

	**Low load (n=30)**	**High load (n=28)**	**Difference between groups**	**p-value (ANCOVA)**
**Anthropometrics**				
Body Weight (kg)	0.01 (-0.5, 0.5)	-0.01 (-0.51, 0.49)	-0.02 (-0.72, 0.68)	0.96
Waist circumference (cm)	0.3 (-0.8, 1.3)	**-2.01 (-3.10, -0.93) *** **	-2.26 (-3.78, -0.74)	**0.004**
**DXA scan**				
Fat percent (%)	0.21 (-0.04, 0.47)	**-0.44 (-0.71, -0.18) *** **	-0.65 (-1.02, -0.29)	**0.0008**
Fat mass (g)	307.03 (-17.95, 632.00)	**-386.81 (-723.33, -50.29) * **	-693.84 (-1164.52, -223.16)	**0.005**
Lean mass (g)	-115.30 (-504.27, 273.66)	**439.97 (37.18, 842.76) * **	555.27 (-8.10, 1118.64)	**0.053**
BMC (g)	0.94 (-7.24, 9.13)	-0.55 (-9.03, 7.93)	-1.49 (-13.35, 10.37)	0.802
**CT scan**				
VAT (cm^3)	-2.60 (-4.92, -0.27.)	-0.17 (-2.58, 2.24)	2.43 (-0.94, 5.80)	0.153
SAT (cm^3)	-1.85 (-4.85, 1.15)	-3.51 (-6.62, -0.40)	-1.66 (-6.01, 2.68)	0.446
Liver fat (HU)	0.13 (-1.23, 1.49)	1.27 (-0.13, 2.68)	1.14 (-0.83, 3.11)	0.251
**Physical activity**				
Mean Daily Activity (mg)	0.22 (-2.23, 2.68)	-1.62 (-4.16, 0.92)	-1.84 (-5.40, 1.71)	0.303
Sedentary time (minutes/day)	**-17.39 (-36.18, 1.39) * **	2.80 (-16.66, 22.25)	20.19 (-7.02, 47.40)	0.143
LPA (minutes/day)	**7.96 (-0.79, 16.72) * **	4.89 (-4.18, 13.95)	-3.08 (-15.76, 9.60)	0.628
MVPA (minutes/day)	-0.35 (-8.14, 8.11)	-6.59 (-15.35, 2.17)	-6.31 (-18.56, 5.94)	0.306
**Energy balance**				
Energy expenditure (MJ/day)	-0.11 (-0.45, 0.23)	-0.17 (-0.52, 0.19)	-0.05 (-0.55, 0.44)	0.831
Energy intake (MJ/day)	-0.02 (-0.85, 0.82)	0.11 (-0.75, 0.98)	0.13 (-1.08, 1.34)	0.829

Table S2. Analyses of the absolute changes in the main secondary endpoints for all participants eligible for full analysis set (FAS). Results are presented as estimated marginal means with 95% confidence intervals. The between group p-values (High load vs Low load) given within the table are calculated using analysis of covariance (ANCOVA) adjusted for age, sex, baseline BMI, Vest exposure (h) and standing % with vest. Within group comparisons (five weeks vs baseline) were made using Wilcoxon signed-rank test. *p<0.05, **p< 0.01, ***p<0.001. Statistically significant differences are highlighted in **bold**. BMC, Bone Mineral Content; BMI, body mass index; CT, Computed Tomography; DXA, Dual-energy X-ray absorptiometry; HU, Hounsfield unit; Liver fat, estimated as liver attenuation; LPA, light physical activity; Mg, milli-g (acceleration); MJ, Megajoules; MVPA, Moderate to vigorous physical activity; SAT, subcutaneous adipose tissue in the abdominal region; VAT, visceral adipose tissue in the abdominal region.

Table S3 – Results: relative changes (Per protocol analysis)

	Low load (n=26)	High load (n=25)	Difference between groups	p-value (ANCOVA)
**Anthropometrics**				
Body weight (%)	0.3 (-0.2, 0.8)	0.1 (-0.5, 0.6)	-0.2 (-0.9, 0.5)	0.569
Waist circumference (%)	0.4 (-0.7, 1.5)	**-1.8 (-2.9, -0.8) ** **	-2.3 (-3.8, -0.7)	**0.005**
**DXA scan**				
Fat percent (%)	**1.0 (0.3, 1.6) * **	**-1.4 (-2.1, -0.7) *** **	-2.4 (-3.3, -1.4)	**1.6E-5**
Fat mass (%)	**1.4 (0.5, 2.2) ** **	**-1.2 (-2.1, -0.4) ** **	-2.6 (-3.8, -1.4)	**6.5E-5**
Lean mass (%)	-0.2 (-0.9, 0.5)	**1.2 (0.5, 1.9) ** **	1.4 (0.4, 2.4)	**0.009**
BMC (%)	0.1 (-0.2, 0.5)	-0.03 (-0.4, 0.3)	-0.2 (-0.6, 0.3)	0.471
**CT scan**				
VAT (%)	-2.3 (-6.2, 1.5)	-1.0 (-4.9, 3.0)	1.4 (-4.2, 6.9)	0.621
SAT (%)	-0.3 (-2.2, 1.5)	**-1.7 (-3.6, 0.1) * **	-1.4 (-4.0, 1.3)	0.298
Liver fat (%)	0.8 (-1.8, 3.4)	2.5 (-0.1, 5.2)	1.7 (-2.1, 5.4)	0.370
**Physical activity**				
Mean Daily Activity (%)	8.1 (-5.3, 21.5)	-2.8 (-16.5, 10.9)	-10.9 (-30.2, 8.4)	0.261
Sedentary time (%)	**-3.4 (-6.0, -0.9) ** **	1.3 (-1.4, 3.9)	4.7 (1.0, 8.4)	**0.014**
LPA (%)	**12.7 (1.5, 23.8) ** **	7.8 (-3.6, 19.2)	-4.9 (-21.0, 11.2)	0.543
MVPA (%)	7.8 (-6.9, 22.5)	-3.2 (-18.1, 11.8)	-11.0 (-32.2, 10.2)	0.302
**Energy balance**				
Energy expenditure (%)	-0.8 (-3.8, 2.1)	-1.5 (-4.5, 1.5)	-0.7 (-4.9, 3.6)	0.746
Energy intake (%)	5.7 (-4.7, 16.1)	0.2 (-10.4, 10.9)	-5.5 (-20.5, 9.6)	0.468

Table S3. Analyses of the relative changes in the primary and main secondary endpoints for all participants eligible for per protocol analysis. The primary endpoint was the relative change in body weight after 5 weeks of treatment. Results are presented as estimated marginal means with 95% confidence intervals The between group p-values (High load vs Low load) given within the table are calculated using analysis of covariance (ANCOVA) adjusted for age, sex, baseline BMI, Vest exposure (h) and standing % with vest. Within group comparisons (five weeks vs baseline) were made using Wilcoxon signed-rank test. *p<0.05, **p< 0.01, ***p<0.001. Statistically significant differences are highlighted in **bold**. BMC, Bone Mineral Content; BMI, body mass index; CT, Computed Tomography; DXA, Dual-energy X-ray absorptiometry; HU, Hounsfield unit; Liver fat, estimated as liver attenuation; LPA, light physical activity; MVPA, moderate to vigorous physical activity; NS, non-significant; SAT, subcutaneous adipose tissue in the abdominal region; VAT, visceral adipose tissue in the abdominal region.

Table S4 – Results: absolute regional body composition changes (Per protocol analysis)

	Low load (n=26)	High load (n=25)	Difference between groups	p-value (ANCOVA)
**Absolute changes**				
**Fat mass**				
Total (g)	**526.8 (213.4, 840.2) ****	**-500.8 (-820.5, -181.1) ****	-1027.6 (-1480.0, -575.3)	**3.8E-5**
Arms (g)	28.0 (-70.3, 126.3)	17.9 (-82.4, 118,2)	-10.1 (-152.0, 131.8)	0.887
Legs (g)	**107.8 (33.4, 182.3) ***	-48.5 (-124.5, 27.4)	-156.4 (-263.8, -48.9)	**0.005**
Trunk (g)	201.8 (-74.1, 477.8)	**-385.4 (-666.9, -103.9) ***	-587.2 (-985.4, -189.0)	**0.005**
Android (g)	71.3 (9.6, 132.9)	**-85.5 (-148.4, -22.6) ****	-156.8 (-245.8, -67.8)	**0.00093**
Gynoid (g)	**104.7 (38.0, 171.4) ***	**-71.0 (-139.0, -2.9) ***	-175.7 (-272.0, -79.5)	**0.00064**
**Lean mass**				
Total (g)	-119.4 (-538.3, 299.5)	**588.8 (161.4, 1016.1) ****	708.2 (103.7, 1312.7)	**0.023**
Arms (g)	-17.3 (-138.3, 103.8)	14.1 (-109.4, 137.6)	31.4 (-143.3, 206.1)	0.719
Legs (g)	-40.1 (-258.2, 178.0)	70.0 (-152.5, 292.5)	110.1 (-204.6, 424.8)	0.484
Trunk (g)	-15.5 (-301.4, 270.4)	**451.0 (159.3, 742.6) ****	466.5 (53.9, 879.0)	**0.028**
Android (g)	-32.5 (-102.3, 37.3)	**106.3 (35.1, 177.4) ****	138.7 (38.1, 239.4)	**0.008**
Gynoid (g)	-46.5 (-156.0, 62.9)	**151.8 (40.1, 263.4) ***	198.3 (40.4, 356.2)	**0.015**
**Relative changes**				
**Fat mass**				
Fat percent (%)	**1.0 (0.3, 1.6) ***	**-1.4 (-2.1, -0.7) *****	-2.4 (-3.3, -1.4)	**1.6E-5**
Total (%)	**1.4 (0.5, 2.2) ****	**-1.2 (-2.1, -0.4) ****	-2.6 (-3.8, -1.4)	**6.5E-5**
Arm (%)	0.3 (-2.3, 3.0)	0.9 (-1.8, 3.6)	0.5 (-3.3, 4.3)	0.781
Legs (%)	**1.9 (0.5, 3.2) ***	-0.6 (-2.0, 0.8)	-2.5 (-4.5, -0.5)	**0.010**
Trunk (%)	1.0 (-0.3, 2.2)	**-1.6 (-2.9, -0.4) ***	-2.6 (-4.4, -0.8)	**0.005**
Android (%)	1.7 (0.3, 3.2)	**-2.1 (-3.6, -0.6) ****	-3.9 (-6.0, -1.7)	**0.00069**
Gynoid (%)	**1.7 (0.7, 2.8) ***	**-1.1 (-2.2, -0.02) ***	-2.8 (-4.3, -1.3)	**0.00059**
**Lean mass**				
Total (%)	-0.2 (-0.9, 0.5)	**1.2 (0.5, 1.9) ****	1.4 (0.4, 2.4)	**0.009**
Arms (%)	0.03 (-1.9, 1.9)	0.4 (-1.5, 2.4)	0.4 (-2.4, 3.2)	0.769
Legs (%)	-0.2 (-1.3, 0.9)	0.6 (-0.6, 1.7)	0.8 (-0.8, 2.4)	0.310
Trunk (%)	0.03 (-1.1, 1.1)	**2.0 (0.9, 3.2) ****	2.0 (0.4, 3.6)	**0.014**
Android (%)	-0.6 (-2.3, 1.2)	**2.9 (1.1, 4.7) ****	3.5 (1.0, 6.0)	**0.008**
Gynoid (%)	-0.5 (-1.7, 0.7)	**1.8 (0.6, 3.0) ***	2.3 (0.6, 4.0)	**0.010**

Table S4. Analyses of the absolute and relative regional changes in body composition for all participants eligible for per protocol analysis. Analyses of body composition in different regions of interests from Dual-energy X-ray absorptiometry scan. Results are presented as estimated marginal means with 95% confidence intervals. The between group p-values (High load vs Low load) given within the table are calculated using analysis of covariance (ANCOVA) adjusted for age, sex, baseline BMI, Vest exposure (h) and standing % with vest. Within group comparisons (five weeks vs baseline) were made using Wilcoxon signed-rank test. *p<0.05, **p< 0.01, *p< 0.001. Statistically significant differences are highlighted in **bold**.

Table S5 – Results: serum/plasma markers (Per protocol analysis)

	Low load (n=26)	High load (n=25)	Difference between groups	p-value (ANCOVA)
Serum/plasma				
Total cholesterol (%)	0.2 (-4.7, 5.1)	3.3 (-1.7, 8.3)	3.1 (-4.0, 10.2)	0.383
LDL cholesterol (%)	-0.03 (-5.8, 5.7)	3.9 (-2.0, 9.7)	3.9 (-4.4, 12.2)	0.351
HDL cholesterol (%)	-0.1 (-4.6, 4.3)	2.4 (-2.1, 7.0)	2.6 (-3.9, 9.0)	0.426
Triglycerides (%)	5.3 (-7.1, 17.6)	5.5 (-7.1, 18.1)	0.2 (-17.6, 18.1)	0.981
Glycerol (%)	12.5 (-9.8, 34.7)	25.2 (3.0, 47.5)	12.7 (-19.0, 44.5)	0.423
Fasting Plasma Glucose (%)	0.5 (-2.9, 3.7)	-1.7 (-5.1, 1.7)	-2.1 (-6.8, 2.7)	0.385
Insulin (%)	-0.9 (-11.4, 9.6)	-1.2 (-11.9, 9.6)	-0.3 (-15.4, 14.9)	0.970
HOMA-IR index (%)	-0.9 (-12.6, 10.8)	-1.7 (-0.1, 10.2)	-0.8 (-17.7, 16.1)	0.923
Leptin (%)	9.9 (-7.2, 27.0)	-3.8 (-21.2, 13.6)	-13.7 (-38.3, 11.0)	0.269

Table S5. Analyses of the relative changes in metabolic markers for all participants eligible for per protocol analysis. The between group p-values (High load vs Low load) given within the table are calculated using analysis of covariance (ANCOVA) adjusted for age, sex, baseline BMI, Vest exposure (h) and standing % with vest. Within group comparison (five weeks vs baseline) made using Wilcoxon signed-rank test. *p<0.05, **p< 0.01, ***p<0.001. HDL, high-density lipoprotein; HOMA-IR, homeostatic model assessment for insulin resistance; LDL, low-density lipoprotein.

Table S6 – Reported adverse events and serious adverse events

**Adverse events, n (%)**		**Low load (n=30)**	**High load (n=29)**	**p-value**
Name	***ICD-10 code***			
**Gastrointestinal disorder**		0 (0)	1 (3.4)	0.49
Functional dyspepsia	K30.9	0 (0)	1 (3.4)	0.49
**Heart and/or lung disorder**		8 (26.7)	3 (10.3)	0.44
Atrial premature depolarization	I49.1	0 (0)	1 (3.4)	0.49
Cough, unspecified	R05.9	1 (3.3)	0 (0)	1.00
Hypertension	I10.9	1 (3.3)	0 (0)	1.00
Other specified cardiac arrhythmias [idioventricular rhythm]	I49.8	0 (0)	1 (3.4)	0.49
Supraventricular tachycardia, unspecified	I47.1	1 (3.3)	0 (0)	1.00
Ventricular premature depolarization	I49.3	3 (10.0)	1 (3.4)	1.00
Ventricular tachycardia	I47.2	2 (6.7)	0 (0)	0.49
**Infections**		14 (46.7)	28 (96.6)	0.12
Acute gastroenteropathy, viral	A08.1	1 (3.3)	0 (0)	1.00
Acute nasopharyngitis [common cold]	J00.9	8 (26.7)	11 (37.9)	0.31
Acute pharyngitis	J02.9	0 (0)	1 (3.4)	0.49
Acute upper respiratory infection	J06.9	1 (3.3)	9 (31.0)	**0.014**
Bacterial foodborne intoxication, unspecified	A05.9	1 (3.3)	0 (0)	1.00
COVID-19	U07.1	1 (3.3)	4 (13.8)	0.20
Fever, unspecified	R50.9	1 (3.3)	1 (3.4)	1.00
Herpes viral infection, oral	B00.9	0 (0)	1 (3.4)	0.49
Infectious gastroenteritis and colitis	A09.9	1 (3.3)	0 (0)	1.00
Tinea cruris	B35.6	0 (0)	1 (3.4)	0.49
**Injury**		5 (16.7)	0 (0)	0.36
Contusion of hip	S70.0	1 (3.3)	0 (0)	1.00
Contusion of shoulder	S40.01	1 (3.3)	0 (0)	1.00
Contusion of thorax	S20.2	2 (6.7)	0 (0)	0.49
Laceration without foreign body of thigh	S71.1	1 (3.3)	0 (0)	1.00
**Metabolic disorders**		0 (0)	1 (3.4)	0.49
Diabetes, type 2	E11.9	0 (0)	1 (3.4)	0.49
**Musculoskeletal disorders**		10 (33.3)	28 (96.6)	**0.027**
Achilles tendinitis	M76.6	1 (3.3)	1 (3.4)	1.00
Backache (postural)	M54.9	2 (6.7)	5 (17.2)	0.25
Lateral epicondylitis	M77.1	1 (3.3)	0 (0)	1.00
Lesion of sciatic nerve, piriformis syndrome	G57.0	1 (3.3)	0 (0)	1.00
Lumbago	M54.5	0 (0)	1 (3.4)	0.49
Myalgia, abdominal muscles	M79.1E	0 (0)	1 (3.4)	0.49

Myalgia, hip/femoral region	M79.1F	0 (0)	4 (13.8)	0.052
Myalgia, shoulder	M79.1	2 (6.7)	5 (17.2)	0.51
Plantar fascial fibromatosis	M72.2	0 (0)	2 (6.9)	0.24
Other synovitis and tenosynovitis, forearm	M65.8	1 (3.3)	0 (0)	1.00
Pain, foot	M79.6H	1 (3.3)	2 (6.9)	0.61
Pain, knee	M25.5G	0 (0)	5 (17.2)	**0.024**
Strain of adductor muscle, fascia and tendon of thigh	S76.2	0 (0)	1 (3.4)	0.49
Strain of muscle, fascia and tendon at neck level	S16.1	0 (0)	1 (3.4)	0.49
Strain of muscle, fascia and tendon of lower back	S39.0	1 (3.3)	0 (0)	1.00
**Neurological disorders**		3 (10.0)	1 (3.4)	0.61
Headache	R51.9	3 (10.0)	1 (3.4)	0.61
**Other**		4 (13.3)	7 (24.1)	0.51
Follicular disorder, abdomen	L73.9	1 (3.3)	0 (0)	1.00
Hypopituitarism	E23.0D	1 (3.3)	0 (0)	1.00
Ingrowing nail	L60.0	0 (0)	1 (3.4)	0.49
Mouth Blister	S00.522	0 (0)	1 (3.4)	0.49
Other malaise and fatigue	R53.9	0 (0)	1 (3.4)	0.49
Other symptoms and signs involving emotional state [Psychological discomfort from vest]	R45.8	0 (0)	1 (3.4)	0.49
Pruritus, unspecified, thorax	L29.8	1 (3.3)	0 (0)	1.00
Rash and other nonspecific skin eruption	R21.9	0 (0)	2 (6.9)	0.24
State of emotional shock and stress	R45.7	1 (3.3)	0 (0)	1.00
**Severe adverse events, n (%)**				
Cellulitis and acute lymphangitis of finger	L03.0C	0 (0)	1 (3.4)	0.49
**Relation to treatment**				
Participants with any adverse events, n (%)		23 (76.7)	26 (89.7)	0.30
Participants with any treatment-related adverse event, n (%)		5 (16.7)	21 (72.4)	**0.00002**
Number of treatment-related adverse events		6 (20.0)	29 (100)	**0.00006**
Any serious adverse event, n (%)		0 (0)	1 (3.4)	0.49
Any treatment-related serious adverse event, n (%)		0 (0)	0 (0)	

Table S6. Reported adverse and serious adverse events among the randomised study participants. Adverse events are presented as number of events together with percentage for all subjects who were randomized. Comparisons between groups calculated with Fisher's exact test. *p<0.05, **p< 0.01, ***p<0.001. Statistically significant differences are highlighted in **bold**.

Table S7 – Medical history at baseline for all randomised participants

Medical history at baseline, n (%)		Low load (n=30)	High load (n=29)	p-value
Name	***ICD-10 code***			
Abnormal ECG	R94.3A	0 (0)	1 (3.6)	0.49
Allergy	T78.4	3 (10.0)	1 (3.6)	0.61
Anisocoria (pupil)	H57.0	1 (3.3)	0 (0)	1.00
Asthma	J45.9	2 (6.7)	1 (3.6)	1.00
Carpal tunnel syndrome	G56.0	0 (0)	1 (3.6)	0.49
Climacteric (female)	N95.1	1 (3.3)	0 (0)	1.00
Crohn's disease	K50.9	0 (0)	1 (3.6)	0.49
Displacement, intervertebral disc	M51.2	1 (3.3)	0 (0)	1.00
Dyslipidaemia	E78.5	1 (3.3)	0 (0)	1.00
Functional dyspepsia	K30.9	2 (6.7)	1 (3.6)	1.00
GERD (gastroesophageal reflux disease)	K21.9	0 (0)	1 (3.6)	0.49
Hallux valgus	M20.1	0 (0)	1 (3.6)	0.49
Hypertension	I10.9	4 (13.3)	1 (3.6)	0.35
Hypothyroidism	E03.9	0 (0)	1 (3.6)	0.49
Hysterectomy	LCD00	1 (3.3)	0 (0)	1.00
Lipedema	R60.0B	1 (3.3)	0 (0)	1.00
Lumbago	M54.5	1 (3.3)	0 (0)	1.00
Migraine	G43.1	1 (3.3)	0 (0)	1.00
Morton's metatarsalgia (neuralgia/neuroma)	G57. 6	0 (0)	1 (3.6)	0.49
Osteoarthritis, knee	M17.9	1 (3.3)	1 (3.6)	1.00
Other reactions to severe stress	F43.8A	0 (0)	1 (3.6)	0.49
Pain, foot	M79.6H	1 (3.3)	2 (7.1)	0.61
Rupture, patella tendon	M66.2G	0 (0)	1 (3.6)	0.49
Sleep apnea	G47.3	0 (0)	1 (3.6)	0.49
Strabismus (congenital)	H50.9	0 (0)	1 (3.6)	0.49

Table S7. Medical history at baseline for all randomised participants. Values presented as number of participants together with percentage. NS denotes not significant. Comparisons between groups calculated with Fisher's exact test. *p<0.05, **p< 0.01, ***p<0.001.

Table S8 – Concomitant medications

Medications during study, n (%)		Low load (n=30)	High load (n=29)	p-value
Name	***ATC-code***			
**Analgetic medication**		8 (26.7)	2 (6.9)	0.32
Acetylsalicylic acid	N02BA51	1 (3.3)	0 (0)	1.00
Diklofenac, topical use	M02AA15	1 (3.3)	1 (3.4)	1.00
Ibuprofen	M01AE01	1 (3.3)	1 (3.4)	1.00
Naproxen	M01AE02	2 (6.7)	0 (0)	0.49
Paracetamol	N02BE01	3 (10.0)	0 (0)	0.24
**Antibiotics, antifungals, anthelmintics, and antiviral medication**		1 (3.3)	6 (20.7)	0.12
Cloxacillin	J01CF02	0 (0)	1 (3.4)	0.49
Flucloxacillin	J01CF05	0 (0)	1 (3.4)	0.49
Covid-19 vaccines	J07BN01	1 (3.3)	1 (3.4)	1.00
Mebendazole	P02CA01	0 (0)	1 (3.4)	0.49
Terbinafine	D01AE15	0 (0)	1 (3.4)	0.49
Valaciclovir	J05AB11	0 (0)	1 (3.4)	0.49
**Antihistamines**		2 (6.7)	1 (3.4)	1.00
Desloratadine	R06AX27	2 (6.7)	0 (0)	0.49
Fexofenadine	R06AX26	0 (0)	1 (3.4)	0.49
**Antihypertensive medication**		5 (16.7)	1 (3.4)	0.48
Amlodipine	C08CA01	2 (6.7)	0 (0)	0.49
Candesartan	C09CA06	1 (3.3)	1 (3.4)	1.00
Losartan	C09CA01	2 (6.7)	0 (0)	0.49
**Beta-2-adrenoreceptor agonists**		1 (3.3)	2 (6.9)	1.00
Salbutamol	R03AC02	1 (3.3)	2 (6.9)	1.00
**Combinations of salt complexes**		0 (0)	1 (3.4)	0.49
Ordinary salt combinations (Novalucol)	A02AD01	0 (0)	1 (3.4)	0.49
**Hormone replacement**		1 (3.3)	1 (3.4)	1.00
Norethisterone and estrogen	G03FA01	1 (3.3)	0 (0)	1.00
Levothyroxine sodium	H03AA01	0 (0)	1 (3.4)	0.49
**Proton pump inhibitors**		3 (10.0)	2 (6.9)	1.00
Esomeprazole	A02BC05	0 (0)	1 (3.4)	0.49
Omeprazole	A02BC01	3 (10.0)	1 (3.4)	0.61
**Statins**		1 (3.3)	0 (0)	1.00
Atorvastatin	C10AA05	1 (3.3)	0 (0)	1.00

Table S8. Medications during the study presented as number of participants together with percentage for all randomised subjects. Comparisons between groups calculated with Fisher's exact test. *p<0.05, **p< 0.01, ***p<0.001.
